# Multimodal diagnostic models and subtype analysis for neoadjuvant therapy in breast cancer

**DOI:** 10.3389/fimmu.2025.1559200

**Published:** 2025-03-18

**Authors:** Zheng Ye, Jiaqi Yuan, Deqing Hong, Peng Xu, Wenbin Liu

**Affiliations:** ^1^ Institute of Computational Science and Technology, Guangzhou University, Guangzhou, China; ^2^ School of Computer Science of Information Technology, Qiannan Normal University for Nationalities, Duyun, Guizhou, China

**Keywords:** BRCA, multimodal diagnostic model, neoadjuvant therapy, heterogeneous disease, breast cancer subtypes, tumor microenvironment

## Abstract

**Background:**

Breast cancer, a heterogeneous malignancy, comprises multiple subtypes and poses a substantial threat to women's health globally. Neoadjuvant therapy (NAT), administered prior to surgery, is integral to breast cancer treatment strategies. It aims to downsize tumors, optimize surgical outcomes, and evaluate tumor responsiveness to treatment. However, accurately predicting NAT efficacy remains challenging due to the disease's complexity and the diverse responses across different molecular subtypes.

**Methods:**

In this study, we harnessed multimodal data, including proteomic, genomic, MRI imaging, and clinical information, sourced from multiple cohorts such as I-SPY2, TCGA-BRCA, GSE161529, and METABRIC. Post data preprocessing, Lasso regression was utilized for feature extraction and selection. Five machine learning algorithms were employed to construct diagnostic models, with pathological complete response (pCR) as the predictive endpoint.

**Results:**

Our results revealed that the multi-omics Ridge regression model achieved the optimal performance in predicting pCR, with an AUC of 0.917. Through unsupervised clustering using the R package MOVICS and nine clustering algorithms, we identified four distinct multimodal breast cancer subtypes associated with NAT. These subtypes exhibited significant differences in proteomic profiles, hallmark cancer gene sets, pathway activities, tumor immune microenvironments, transcription factor activities, and clinical characteristics. For instance, CS1 subtype, predominantly ER-positive, had a low pCR rate and poor response to chemotherapy drugs, while CS4 subtype, characterized by high immune infiltration, showed a better response to immunotherapy. At the single-cell level, we detected significant heterogeneity in the tumor microenvironment among the four subtypes. Malignant cells in different subtypes displayed distinct copy number variations, differentiation levels, and evolutionary trajectories. Cell-cell communication analysis further highlighted differential interaction patterns among the subtypes, with implications for tumor progression and treatment response.

**Conclusion:**

Our multimodal diagnostic model and subtype analysis provide novel insights into predicting NAT efficacy in breast cancer. These findings hold promise for guiding personalized treatment strategies. Future research should focus on experimental validation, in-depth exploration of the underlying mechanisms, and extension of these methods to other cancers and treatment modalities.

## 1 Introduction

Breast cancer (BC) is a prevalent malignant tumor among women globally, posing a significant threat to female health (1, 2). Despite advancements in medical technology and the continuous innovation of diagnostic and therapeutic methods leading to a decrease in mortality rates, the incidence of breast cancer has shown a marked increase over the past four decades (3, 4). According to the 2020 statistical data, there were approximately 2.3 million new cases and 685,000 deaths worldwide, with notable regional disparities and a higher prevalence in high-income countries (5–7). It is projected that by 2040, the annual number of new breast cancer cases could surpass 3 million, with the death toll potentially exceeding 1 million (7, 8).

Breast cancer is classified into molecular subtypes based on the expression levels of estrogen receptor (ER), progesterone receptor (PR), human epidermal growth factor receptor 2 (HER2), and the proliferation marker Ki-67 (9, 10). These subtypes include Luminal A (ER+ and/or PR+, HER2−, low Ki-67), Luminal B (ER+ and/or PR+, HER2− with high Ki-67 or HER2+ regardless of Ki-67 status), HER2-enriched (ER−, PR−, HER2+, with ERBB2 overexpression), and Triple-negative (TN) (ER−, PR−, HER2−) (11–13). These molecular subtypes serve as critical prognostic indicators and guide the selection of pre- and post-operative systemic therapies, which often target these receptors (14). Luminal A breast cancer has the best prognosis and is typically treated with endocrine therapy (15); Luminal B has a good prognosis and can be treated with endocrine therapy, cytotoxic chemotherapy, or targeted therapy (16); HER2-enriched breast cancer is now commonly treated with a combination of targeted therapy and cytotoxic chemotherapy, significantly improving prognosis (17, 18); TN breast cancer remains the most aggressive subtype, primarily treated with cytotoxic (neoadjuvant) chemotherapy (19, 20). However, these classifications may not accurately reflect the heterogeneity of breast cancer, as genomic characteristics and expression patterns can vary significantly among individuals (21). Consequently, there has been a growing interest in understanding the genomic landscape of breast cancer to identify novel molecular markers and therapeutic targets (22–24). For instance, mutations in the BRCA1 and BRCA2 genes have garnered considerable attention due to their association with familial inheritance risk of breast cancer and their potential impact on molecular characteristics and treatment response (25, 26).

Neoadjuvant therapy (NAT) is a strategic approach to treating breast cancer, administered prior to surgery with the intent of reducing tumor size and the extent of lymph node involvement, thereby enhancing the success rate of surgical resection (27–29). NAT encompasses a combination of chemotherapy, endocrine therapy, and targeted therapy (30). Chemotherapy aims to kill tumor cells using cytotoxic agents, which target rapidly dividing cells. While this is effective against many cancer cells, it also affects non-cancerous, highly proliferative cells in the body, such as those in the bone marrow, hair follicles, and gastrointestinal tract, leading to common side effects (31, 32). The goal of NAT is to reduce the tumor burden preoperatively, improve surgical outcomes, and decrease the risk of postoperative recurrence and metastasis (33). Pathologic complete response (pCR), defined as the absence of invasive tumor in the breast and lymph nodes (ypT0/is; ypN0), is a crucial prognostic factor in breast cancer management (34, 35). This treatment outcome is associated with improved disease-free and overall survival, underscoring the importance of accurately predicting the response to neoadjuvant therapy (NAT) (36). The ability to accurately forecast NAT response is a critical component in clinical decision-making for breast cancer patients. By identifying patients likely to achieve a pCR, clinicians can optimize treatment strategies, potentially sparing patients from unnecessary toxicity while maximizing the likelihood of favorable long-term outcomes (37).

However, predicting the efficacy of NAT in breast cancer is complex due to the heterogeneous nature of the disease and the varying responses to treatment across different molecular subtypes (38). The efficacy of NAT varies significantly depending on the molecular subtype of breast cancer. HER2-positive and triple-negative breast cancers (TNBC) generally show higher rates of pCR to chemotherapy-based NAT compared to Luminal A tumors, which are often less responsive to chemotherapy (37, 39). Multimodal breast cancer data, which often reflect the molecular and pathological diversity of breast cancer, can be leveraged to improve the accuracy of NAT response prediction (40–42). Despite this, there is a paucity of multimodal predictive models for breast cancer NAT efficacy, and no studies have yet explored multimodal molecular subtypes in this context.

In this study, we integrated proteomic, genomic, and MRI imaging data from breast cancer to construct a predictive model for NAT response. We also employed nine unsupervised clustering methods to establish novel multimodal molecular subtypes based on three modal features related to NAT. By comparing these molecular subtypes at the tissue sequencing level and single-cell sequencing level, we revealed unique characteristics in the tumor microenvironment features of these subtypes. Our multimodal predictive model and molecular subtypes for breast cancer NAT offer a novel approach to assist clinicians in making informed diagnostic and therapeutic decisions and provide new insights into the progression of breast cancer.

## 2 Methods

### 2.1 Data source and preprocessing

The discovery cohort is sourced from the I-SPY2 trial (43, 44) (The Cancer Imaging Archive, https://www.cancerimagingarchive.net/collection/ispy2/), while the validation cohorts originate from TCGA-BRCA (45) (cBioPortal (46, 47), https://www.cbioportal.org/), GSE161529 (48) (TISCH2 (49), tisch.comp-genomics.org/gallery/), and Breast Cancer (METABRIC (50, 51), www.cbioportal.org). Our primary focus is on comparing and analyzing the clinical and tissue microenvironment differences among breast cancer multimodal subtypes using the validation cohorts.

The I-SPY2 cohort comprises data from 719 patients, including MRI scans, proteomic data, expression profiles, and clinical information. After thorough curation, we retained 678 samples with complete multimodal information. Expression profile data from the TCGA-BRCA cohort were standardized using log2(TPM+1). Mutation data underwent processing with maftools, while methylation data were processed using CHAMP (52). Proteomic and microbiome information were standardized using data from cbioportal. mRNA microarray data from the METABRIC cohort were log transformed. CIBERSORT (53) was employed to infer the composition of 22 immune cell types based on the gene expression profiles of the samples. The algorithm uses support vector regression to deconvolute the gene expression matrix, providing an estimate of the relative proportions of each immune cell type in the samples. Progeny (54) was utilized to infer differential activity across 13 major signaling pathways. Progeny computes pathway activity scores by integrating the expression of predefined sets of target genes that respond to the activity of 13 major signaling pathways, including pathways such as MAPK, NF-κB, and PI3K. These scores provide a quantitative measure of the differential activity of these pathways across samples. The xCell algorithm is used to evaluate the infiltration scores of 64 common cell types based on transcriptome data. This computational method provides a comprehensive assessment of cellular composition within a given sample by deconvolving the transcriptional profiles (55). Seurat V4 (56) was used for processing and analyzing the GSE161529 cohort’s 53 breast cancer samples from the 10x Genomics platform. Summation of expression profile data across samples yielded bulk RNAseq data for the 53 samples (57, 58). The R package SCP was employed for data preprocessing, including linear dimensionality reduction (PCA), unsupervised clustering (Louvain), and nonlinear dimensionality reduction (UMAP).

The I-SPY2 multicenter trial provided a comprehensive, highly curated imaging dataset, including pre-NAC MRI scans and corresponding Regions of Interest (ROIs) for direct utilization. However, due to the multicenter nature of the trial, voxel sizes of each patient’s MRI were resampled to 1 x 1 x 1 mm^3^. Additionally, given the wide distribution of MRI intensity values, z-score normalization was applied to render image intensities with standard normal distribution characteristics. Following MRI preprocessing, the Pyradiomics (59) module (https://github.com/Radiomics/pyradiomics) was utilized to extract features from tumor ROIs using various filters (Gaussian, Laplacian, high-pass, and low-pass filters), generating additional derived images. All radiomic features were categorized into seven classes (1): 306 First Order Features (2); 14 Shape Features (3); 408 Gray Level Co-occurrence Matrix (GLCM) Features (4); 272 Gray Level Size Zone Matrix (GLSZM) Features (5); 272 Gray Level Run Length Matrix (GLRLM) Features (6); 85 Neighbouring Gray Tone Difference Matrix (NGTDM) Features (7); 238 Gray Level Dependence Matrix (GLDM) Features. A total of 1595 features were extracted from each patient’s ROI and respective MRI sequences.

Cell type annotations for the GSE161529 dataset were obtained from the TISCH2 database (http://tisch.comp-genomics.org/home/), which provides pre-computed cell type labels based on marker gene expression and, commonly, inferred copy number variations. We adopted the ‘Epithelial’ and ‘Malignant’ cell labels as provided by TISCH2, assuming their reasonable accuracy based on standard single-cell analysis practices. Detailed cell annotation and downstream Seurat analysis objects are available from Chen et al (60).

### 2.2 Feature extraction

For the radiomic features extracted using Pyradiomics, we employed a two-step normalization process to ensure the data was appropriately scaled and standardized. First, we applied Min-Max scaling to ensure that each feature value fell within the range of -1 to 1. This scaling technique linearly transforms the data to a common scale, mitigating the potential influence of differing feature value ranges.

Subsequently, we applied Z-score normalization to the scaled radiomic features. This standardization method transformed the features to have a mean of 0 and a standard deviation of 1, effectively removing the original scale and distribution of the data. The resulting standardized radiomic feature set was used for further analysis, promoting the comparability and interpretability of the features across the study population.

Lasso regression was utilized to perform feature selection on the radiomic, proteomic, transcriptomic, and clinical features extracted from MRI images (61). Through 1000 permutations, features with a weight standard deviation greater than 0 were selected for model construction. After filtering features from each modality, the information from multiple modalities was directly merged, and Lasso regression was employed again to extract features from the combined modalities. This approach enabled the construction of machine learning models using features from multiple modalities.

### 2.3 Machine learning models

In this study, Orange3 (62) was employed for constructing machine learning models. Features selected through Lasso regression were utilized, and models were built using five commonly employed algorithms: Lasso Regression, Ridge Regression, Random Forest, Gradient Boosting, and Support Vector Machines (SVM). The predictive outcome measure for the models was pathological complete response (pCR). To identify the best-performing model, we compared the performance of these algorithms across four modalities. Model evaluation was primarily based on six metrics: AUC (Area Under the Curve): AUC assesses the overall performance of a model by evaluating its ability to correctly rank instances from different classes. Higher AUC values indicate better predictive performance.CA (Classification Accuracy): CA calculates the proportion of correctly classified instances out of the total instances, providing an overall measure of the model’s accuracy; F1 Score: The F1 score, which is the harmonic mean of precision and recall, offers a balanced measure of a model’s performance. It considers both the model’s ability to identify positive instances (precision) and its ability to capture all positive instances (recall); Precision: Precision measures the proportion of correctly predicted positive instances out of the total instances predicted as positive, reflecting the model’s ability to minimize false positives; Recall: Also known as sensitivity or true positive rate, recall measures the proportion of correctly predicted positive instances out of the total actual positive instances. It reflects the model’s ability to minimize false negatives; MCC (63) (Matthews Correlation Coefficient): MCC considers true positives, true negatives, false positives, and false negatives to evaluate a model’s performance.

### 2.4 Multi-omics subtypes of neoadjuvant therapy

The R package MOVICS was employed for unsupervised clustering of multi-modal breast cancer information (64). Initially, we utilized data from three modalities: 46 protein expression profiles, 60 mRNA expression profiles, and 42 radiomic features extracted during feature selection. Subsequently, the optimal number of clusters was determined using the getClustNum function. This function utilizes two measurements, namely the clustering prediction index (CPI) and Gap-statistics, to identify the optimal number of clusters for multi-omics integrative clustering. Essentially, the peaks reached by the red (CPI) and blue (Gap-statistics) lines guide the determination of ‘N.clust’. Following the determination of the optimal cluster number, we performed clustering analysis using the getMOIC function. We employed nine clustering algorithms: SNF(Similarity Network Fusion) (65), PINSPlus (66), NEMO (67), COCA() (68), LRAcluster (69), consensusClustering (70), IntNMF (71), CIMLR(called cancer integration via multi-kernel learning) (72), MoCluster (73), and iClusterBayes (74). These algorithms offer diverse approaches to analyze and cluster multi-modal breast cancer data, enabling a comprehensive exploration of underlying patterns and structures within the dataset. Furthermore, we utilized the NTP algorithm (75) to predict breast cancer subtypes based on single-cell aggregated bulk RNA sequencing data from the TCGA-BRCA and GSE161529 datasets. Samples with an adjusted P-value < 0.05 were selected for subsequent analysis.

### 2.5 Analysis of epithelial cells and malignant cells using inferCNV

As epithelial cells constitute the primary cell type involved in breast cancer progression, we extracted epithelial cells and malignant cells from the single-cell dataset for analyzing differences among various breast cancer subtypes. Using fibroblast and endothelial cells as references, we employed inferCNV (76) to analyze copy number variations (CNVs) in epithelial cells and malignant cells. We employed two modes in inferCNV, with cluster_by_groups set to TRUE and FALSE, respectively. By disregarding group clustering, we obtained hierarchical clustering plots for epithelial cells and malignant cells. The height of each group in the clustering plot represents the clonal evolution landscape of each cell subgroup. InferCNV was used to infer large-scale chromosomal copy number variations (CNVs) in the ‘Epithelial’ and ‘Malignant’ cells, as defined by the TISCH2 annotation. Fibroblasts and endothelial cells were used as reference cells, representing cells with expected genomic stability. InferCNV compares the gene expression profile of each cell to the average expression profile of the reference cells, using a moving average across the genome to identify regions of gain or loss. We expect that malignant cells, due to genomic instability, will generally exhibit a greater extent of CNVs compared to non-malignant cells.

### 2.6 Analysis of molecular evolutionary trajectories for epithelial cells and malignant cells

To compute the evolutionary trajectories of epithelial cells and malignant cells, we utilized two algorithms: CytoTrace (77) and Monocle2 (78). Initially, we conducted evolutionary trajectory analysis for epithelial cells and malignant cells using CytoTrace. Subsequently, employing Monocle2, we performed pseudotime analysis separately for each breast cancer subtype to analyze the differentiation process of each subtype. Finally, we analyzed the branches of the evolutionary trajectories for epithelial cells and malignant cells in each breast cancer subtype, thereby exploring the key signaling pathways involved in the differentiation of each cell subtype.

### 2.7 Transcription factor enrichment analysis

For bulk RNA-seq data, transcription factor enrichment analysis was conducted using Dorothea. Regulons were selected from the Dorothea_hs (79, 80) database at confidence levels A, B, and C for analysis. The activity of each transcription factor in every sample was calculated using the run_viper function.

For single-cell RNA-seq (scRNA-seq) data, transcription factor activity was inferred using SCENIC (81). The annotation file hg19-tss-centered-10kb-7species.mc9nr.feather was utilized in the runSCENIC_2_createRegulons function, with the coexMethod parameter set to “top5perTarget” for optimal performance. The analysis was performed as previously described [21]. Initially, the gene regulatory network was constructed using the grn function in pySCENIC (82) (version 0.11.1) with standard settings. Regulons were subsequently identified using the ctx function with the –mask_dropouts parameter. The area under the curve (AUC) was computed using the aucell function with standard settings. The regulon specificity score was calculated per regulon and cell type to obtain a cell type-specific ranking of regulons. For visualization purposes (refer to FIGREF for graphs of, e.g., SOX10), the top 30 downstream targets (ranked by “importance”; see Supplementary Dataset 4 for all regulons) and the top 5 secondary targets were plotted in a directed graph.

### 2.8 Analysis of cells associated with pCR

To identify cells associated with pathological complete response (pCR) in the single-cell data queue, we utilized the Scissor (83, 84) algorithm. This algorithm employs specific criteria to filter cells that exhibit a correlation with pCR. The Scissor algorithm was configured with an alpha parameter of 0.5 and a family parameter set to “binomial,” based on empirical observations. These settings were chosen to optimize the identification of cells relevant to pCR. By applying the Scissor algorithm, we effectively isolated a subset of cells that are most pertinent to the pCR outcome. This subset can now be subjected to further analysis to explore their molecular characteristics and potential implications in breast cancer treatment response.

### 2.9 Cell communication analysis

We conducted cell-cell communication analysis using the CellChat (85) V2 software on 53 BRCA scRNAseq samples. For each pair of cell types, we identified and quantified ligand-receptor (L-R) interactions. These interactions are determined by the projection profiles of ligands and receptors, where the expression levels of L and R approximate their geometric mean within a single cell type. They represent the strength of interaction between all expressed ligands and their receptors within two given cell types, referred to as “probability” in CellChat. It is important to note that CellChat analysis, based on mRNA expression, infers the potential for cell-cell interactions. It does not directly measure protein levels, post-translational modifications, or ligand-receptor binding. Therefore, our interpretations are based on the potential for communication, and further experimental validation would be required to confirm these interactions *in vivo*.

CellChat infers biologically significant cell-cell communication by assigning a probability value to each interaction and conducting a permutation test. It integrates gene expression with prior knowledge of interactions between signaling ligands, receptors, and their cofactors using the law of mass action. The number of inferred ligand-receptor pairs depends on the method used to calculate the average gene expression per cell group. By default, CellChat employs a statistically robust mean method called “trimean,” which yields fewer interactions but excels at predicting stronger interactions, facilitating the selection of interactions for further experimental validation. It’s crucial to note that besides L-R pairs, CellChat also considers crucial signaling factors such as isoform complexes involved in each interaction. Thus, the absence of any of these components results in no interaction. We excluded genes expressed in less than 20% of cells within a cell type and only considered communications that were statistically significant (p < 0.05, permutation test). When computing ligand-receptor pairs, our focus was primarily on the overall number and strength of interactions.

### 2.10 Potential compounds detection

We searched the Connectivity Map (CMap) database (86) (https://clue.io/cmap) to identify potential chemicals that could induce opposite transcriptomic alterations to those observed in the nonresponse group compared to the response group. CMap is a comprehensive library of cellular signatures that captures responses to various chemical, genetic, and disease perturbations. By comparing the transcriptomic changes in our samples with those induced by perturbagens in the CMap library, we can predict drugs and their annotated mode of action (MoA) (87). This process involves using differentially expressed genes as input to identify corresponding target drugs and their MoA. Additionally, the Genomics of Drug Sensitivity in Cancer (88) (GDSC) database contains genomic expression profiles of numerous cell lines and their drug response data, measured by the half-maximal inhibitory concentration (IC50). The GDSC is divided into two datasets: GDSC1, which includes 958 cell lines and 367 drugs, and GDSC2, which includes 805 cell lines and 198 drugs. We utilized the data from GDSC to predict responses to various drugs using the oncoPredict (89) package in R. We also utilized the pRRophetic (90) to evaluate the half-maximal inhibitory concentration (IC50) of commonly used breast cancer drugs, including Cisplatin, Paclitaxel, Gemcitabine, and Vinorelbine. The pRRophetic method is a computational approach that leverages gene expression data to predict the sensitivity of cancer cell lines to various pharmacological agents. By applying this algorithm to our dataset, we were able to estimate the IC50 values for these chemotherapeutic drugs across the breast cancer samples.

### 2.11 Immunetherapy score estimate

The EaSIeR (91) framework calculates the following scores to assess immune infiltration and response in the tumor microenvironment: Cytolytic Activity (CYT) (92), Roh Immune Score (Roh_IS) (93), Chemokine Features (Chemokines) (94), Davoli Immune Features (Davoli_IS) (95), IFNγ Features (IFNγ) (96), Ayers Expanded Immune Signature (Ayers_expIS) (96), T Cell Inflammation Features (Tcell_inflamed) (96), Regulatory Immune Response (RIR) (97), and Tertiary Lymphoid Structure Features (TLS) (98). These scores aid researchers in better understanding the tumor immune microenvironment and predicting the potential efficacy of immunotherapy.

TIDE (99) (Tumor Immune Dysfunction and Exclusion) is a computational framework for assessing immune therapy response using tumor tissue gene expression profiles. Through the TIDE framework, researchers can obtain 12 immune scores associated with immune therapy response: TIDE, IFNG, MSI Score, CD274, CD8, CTL.flag, Dysfunction, Exclusion, MDSC, CAF, TAM, M2, and CTL.

### 2.12 Statistical analysis

All statistical analyses were conducted using R (v4.2+) and Python (v3.8+). Data visualization was performed using the ggplot2 R package (v3.4). A two-tailed P-value < 0.05 was considered statistically significant, denoted as follows: * P < 0.05, ** P < 0.01, *** P < 0.001, **** P < 0.001. Differential gene expression analysis was performed using DESeq2 (v1.38)^100^ for bulk RNA-sequencing data and the non-parametric Wilcoxon rank-sum test for single-cell RNA-sequencing (scRNA-seq) data, accounting for the non-normal distribution typically observed in scRNA-seq datasets. For the METABRIC breast cancer cohort, differential expression was assessed using the limma R package (v3.54) (100). Functional enrichment of gene sets was carried out using Metascape (v3.5) (101), and Gene Set Enrichment Analysis (GSEA) (102) was performed with the ClusterProfiler R package (v4.6). To facilitate downstream analyses and data exploration, we employed both linear and non-linear dimensionality reduction techniques. Principal Component Analysis (PCA), implemented via the R package SCP, was applied to multi-omics data (proteomics, transcriptomics, and radiomics) to reduce dimensionality while retaining maximal variance, thus aiding in unsupervised clustering and machine learning model building. Additionally, Uniform Manifold Approximation and Projection (UMAP) was used for non-linear dimensionality reduction, particularly for visualizing single-cell data and preserving both local and global data structures, thereby revealing complex, non-linear relationships within the tumor microenvironment. For comparisons across multiple groups (e.g., IC50 values of chemotherapy drugs across multimodal subtypes), the non-parametric Kruskal-Wallis test was utilized due to its robustness to deviations from normality and unequal variances. Comparisons between two groups (e.g., immune therapy-related gene set scores between pCR-positive and pCR-negative samples) were conducted using the Student’s t-test, assuming normality and homogeneity of variances. Machine learning models were implemented using the Orange3 platform (v3.32). Lasso Regression employed L1 regularization (C=1). Extreme Gradient Boosting (XGBoost) utilized 100 trees, a learning rate of 0.3, a maximum tree depth of 6, and a regularization parameter (λ) of 1. Support Vector Machine (SVM) was implemented with a cost parameter (C) of 1.0, a regression loss epsilon of 0.1, and a radial basis function (RBF) kernel. Ridge Regression used L2 regularization (C=1). Random Forest was configured with 10 trees, 5 attributes per split, and a minimum of 5 instances for splitting.

## 3 Results

### 3.1 Multi-omics-based diagnostic models for accurate prediction of neoadjuvant chemotherapy efficacy in breast cancer

We utilized features extracted from Protein, mRNA, and MRI imaging omics data, along with clinical characteristics, to construct diagnostic models with Lasso regression as the training target and AUC as the performance metric. Through 1,000 permutations, we obtained feature weight information for each modality. Employing Orange3, we built five models, including Lasso Regression, SVM, Gradient Boosting, Random Forest, and Ridge, which are commonly used machine learning models for the diagnosis of pathological complete response (pCR).

**Figure 1:**
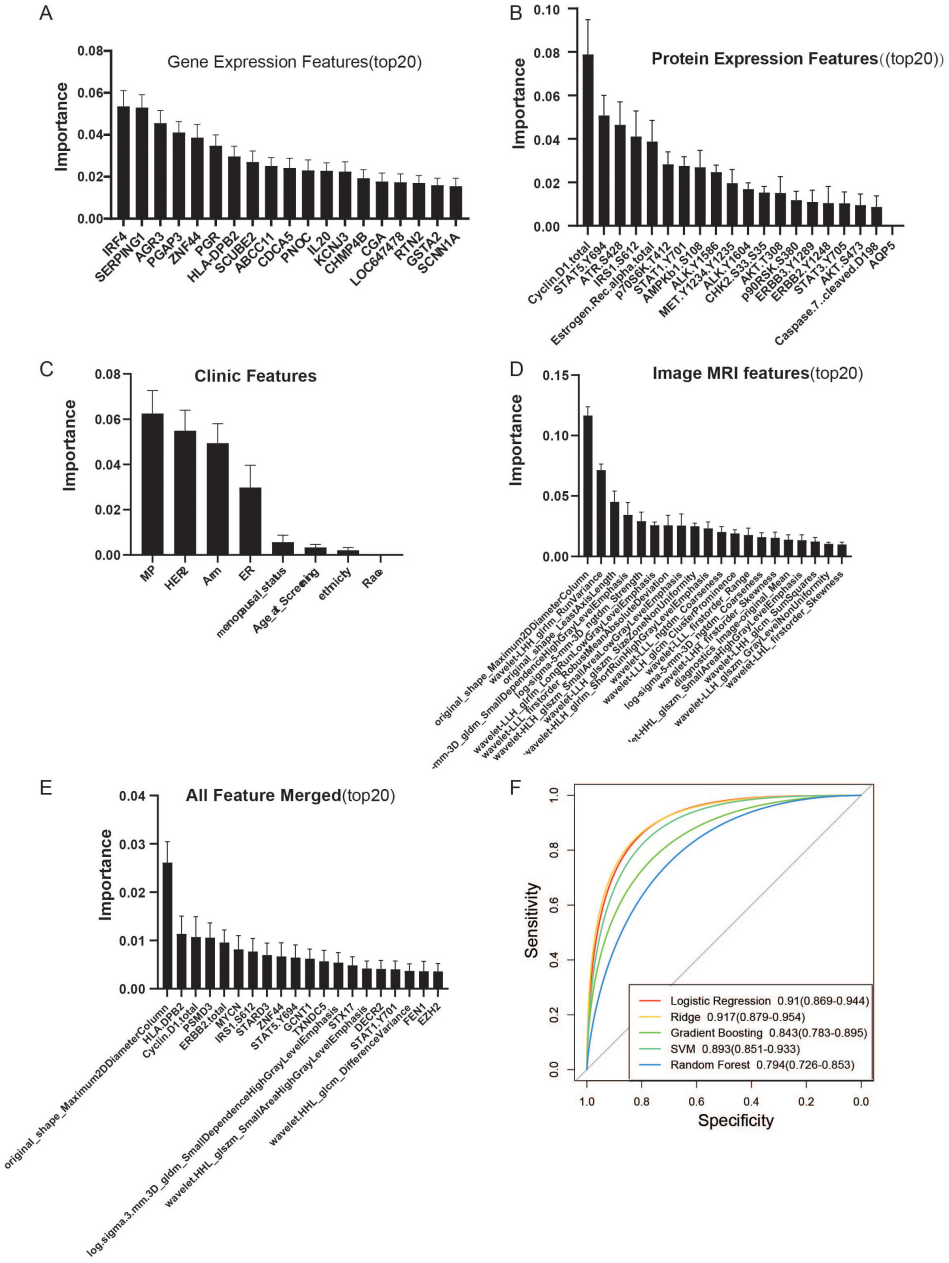
Construction of a multimodal diagnostic model for neoadjuvant therapy in breast cancer. Non-zero weight features were selected through 1000 permutations using Lasso regression for the development of the diagnostic model. **(A)** Top 20 genes by weight from gene expression data, with a total of 60 features. **(B)** Top 20 features by weight from protein expression profiles, with a total of 46 features. **(C)** Feature weights from clinical information. **(D)** Top 20 features by weight from MRI data, with a total of 42 features. **(E)** Weight information of the top 20 features after integrating the four modalities, resulting in a total of 32 features, including 1 clinical feature (ER), 6 radiomics features, 6 proteomics features, and 19 genomics features. **(F)** AUC obtained from five common machine learning models (Logistic Regression, Ridge, Gradient Boosting, SVM, Random Forest) using the constructed multimodal diagnostic model for neoadjuvant therapy in breast cancer. The Ridge model showed the best diagnostic performance with an AUC of 0.917 (0.879-0.964).

In the mRNA expression data, we obtained 60 features, including genes such as IRF4, SERPING1, AGR3, PGAP3, ZNF44, PGR, and HLA-DPB2. The model based on transcriptomics information achieved the highest AUC of 0.884 and CA of 0.808, with an F1 score of 0.805, precision of 0.806, recall of 0.808, and MCC of 0.580 in the validation set(**Figure 1A** **;** **Supplementary Figures S1A, B**). Within the proteomics data, we identified 46 features, including Cyclin.D1.total, STAT5.Y694, ATR.S428, IRS1.S612, Estrogen.Receptor.alpha.total, p70S6K.T412, and STAT1.S108. The model constructed using proteomics information achieved the highest AUC of 0.768 with Ridge, while Lasso Regression yielded the highest classification accuracy (CA) of 0.675, F1 score of 0.658, precision (Prec) of 0.661, recall (Recall) of 0.675, and Matthew’s Correlation Coefficient (MCC) of 0.263(**Figure 1B**; **Supplementary Figure S1C, D**; **Supplementary Table S1**, **S2**).Clinical features such as MP, HER2, Arm, and ER were also weighted. The best model derived from clinical information was Ridge, with an AUC of 0.725, CA of 0.680, F1 score of 0.632, precision of 0.679, recall of 0.680, and MCC of 0.256(**Figure 1C**; **Supplementary Figures S1E, F**). From the MRI radiomics information, we extracted 42 features, including original_shape_Maximum2DDiameterColumn, wavelet-LHH_glrlm_RunVariance, and original_shape_LeastAxisLength, among other geometric features. The Ridge regression model built with radiomics features achieved the best AUC of 0.753, CA of 0.685, F1 score of 0.642, precision of 0.683, recall of 0.685, and MCC of 0.271(**Figure 1D**; **Supplementary Figures S1G, H**). Integrating these features, we performed feature engineering for pCR outcomes using Lasso regression on a total of 148 features. We identified 32 features that could accurately predict the outcome events, including 1 clinical feature (ER), 6 radiomics features, 6 proteomics features, and 19 genomics features. Among them, original_shape_Maximum2DDiameterColumn had the highest feature weight (**Figures 1E, F**; **Supplementary Figures S2A, B**). By constructing a machine learning model with the combined information, we found that Ridge regression provided the best predictive performance, with an AUC of 0.917, CA of 0.823, F1 score of 0.818, precision of 0.822, recall of 0.823, and MCC of 0.611. This result outperformed any single-omics approach and utilized the fewest number of features. In summary, our Ridge regression model, which integrates data from three omics domains and clinical information, achieved the best prediction of pathologic complete response (pCR), demonstrating the superiority of a multi-omics approach for predicting the efficacy of neoadjuvant chemotherapy in breast cancer.

The Matthews Correlation Coefficient (MCC) of 0.611 achieved by the multi-omics Ridge regression model is particularly noteworthy. While an MCC of 1 represents perfect prediction, and 0 indicates performance equivalent to random chance, a value of 0.611 signifies a moderate positive correlation between the model’s predictions and the true pCR outcomes. This is a substantial improvement over the MCC values obtained from the individual omics and clinical models (ranging from 0.256 to 0.580), highlighting the synergistic value of integrating diverse data types. This level of correlation, although not perfect, indicates a clinically relevant predictive capacity, suggesting that the multi-omics model captures important biological interactions and dependencies that are not evident when analyzing each data type in isolation. The improved MCC further validates the robustness of our multi-omics approach, especially considering the inherent complexities and potential class imbalances in predicting treatment response in breast cancer.

### 3.2 Characteristics of multimodal subtypes of breast cancer neoadjuvant therapy constructed by diagnostic model

**Figure 2:**
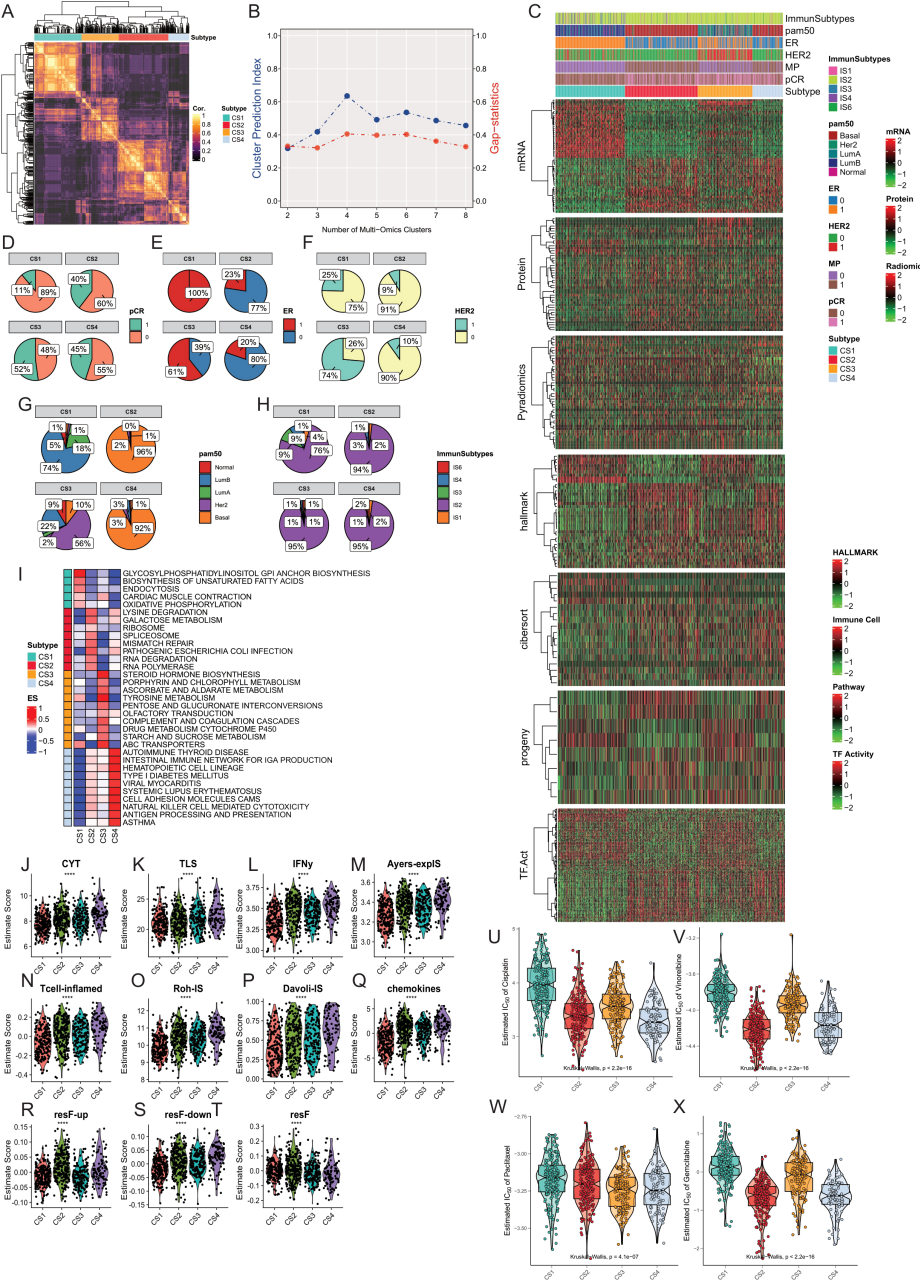
Construction and Comparison of Multimodal Subtypes in Breast Cancer. **(A, B)** The optimal number of subtypes was determined to be four using the Cluster Prediction Index and Gap-statistics. **(C)** Clinical and molecular characteristics of the four breast cancer multimodal subtypes were compared, including significantly different mRNA, protein, pyradiomics, hallmark pathways, CIBERSORT immune infiltration scores, Progeny pathway activities, and transcription factor activities. The four subtypes exhibit distinct differences in these characteristics. **(D)** Distribution of pCR across the four subtypes. **(E)** Distribution of ER status across the four subtypes. **(F)** Distribution of HER2 status across the four subtypes. **(G)** Distribution of PAM50 classifications across the four subtypes. **(H)** Distribution of TCGA-immune subtypes across the four subtypes. **(I)** Comparison of pathway activities among the four breast cancer multimodal subtypes using GSVA scores, with mean scores representing pathway activities for each subtype. **(J–T)** Comparison of ten immune therapy scores among the four breast cancer multimodal subtypes, revealing that the CS4 subtype responds better to immune therapy. **(U–X)** Evaluation of drug response (IC50) for four chemotherapeutic agents (Cisplatin, Vinorelbine, Paclitaxel, Gemcitabine) across the four subtypes, showing significant differences (Kruskal-Wallis, P < 0.001), with the CS1 subtype exhibiting higher IC50 values, indicating resistance to common breast cancer chemotherapy drugs.

We conducted unsupervised clustering analysis by integrating information from three omics layers (proteomics, transcriptomics, and radiomics) in the ISPY2 breast cancer cohort study, aiming to explore the multimodal subtypes of breast cancer. We integrated 46 features from proteomics, 60 features from transcriptomics, and 42 features from radiomics. To determine the optimal number of clusters, we utilized two metrics, Cluster Prediction Index (71) and Gap-statistics (103), searching for cluster numbers from two to eight. Through the analysis of these metrics, we found that the optimal cluster number was four, which could accurately capture the latent structure and patterns in the data. Subsequently, using this optimal cluster number, we conducted further cluster analysis to reveal the characteristics of multimodal subtypes of breast cancer and related biological information. We analyzed the robustness of module clustering of the four clusters through nine unsupervised clustering algorithms (**Figures 2A, B**). CS1 contained 207 samples, CS2 contained 217 samples, CS3 contained 163 samples, and CS4 contained 91 samples. We found that CS1 and CS3 demonstrated robust clustering, while CS2 and CS4 exhibited a low degree of inconsistency across different clustering algorithms.

We compared the differences in the four subtypes from the perspectives of proteomics, hallmark cancer gene sets, pathway activities, tumor immune microenvironment, and transcription factor activities (**Figure 2C**). At the protein level, we found that PTEN.total, Estrogen.Rec.alpha.total, ERBB4.total, Cyclin.D1.total were significantly higher in CS1 compared to the other three subtypes. ERBB2.total, EGFR.Y1068 were significantly higher in CS3, while Ki67.total and Cyclin.B1.total were significantly upregulated in CS2 and CS4 (**Supplementary Figures S3A, B**). Hallmark cancer gene set ssGSEA results showed significant enrichment of ESTROGEN-RESPONSE-EARLY, ESTROGEN-RESPONSE-LATE in CS1, and G2M-CHECKPOINT, INFLAMMATORY-RESPONSE, ALLOGRAFT-REJECTION in CS2 and CS4 (**Supplementary Figure S3C**). We found that p53 pathway activity was significantly elevated in CS1 and CS3, while MAPK pathway activity was significantly decreased. Hypoxia pathway activity was significantly lower than the other three groups. EGFR activity and TGFb activity were highest in CS3, corresponding to the results from proteomics (**Supplementary Figure S3D**).Using the CIBERSORT algorithm, we obtained relative scores of 22 immune cells in each sample based on transcriptomics data. Our results showed that Mast-cells-resting had the highest score in CS1, while most inflammation-related cells had higher scores in CS2 and CS4, including T cells follicular helper, Macrophages M0, T cells CD4 memory activated, NK cells activated, T cells gamma delta. We found that Macrophage M2 had the lowest score in CS2, indicating different characteristics of Macrophage M2 polarization. Mast cell activated had the lowest score in CS1, indicating the potential role of mast cell dormancy and activation status in CS1. Additionally, to compare the differences in other stromal cells in tumor tissue, we used xCell to evaluate 64 cell types and three comprehensive scores in 678 samples. We also found a significant increase in inflammation-related cells in CS4 compared to other tissues (**Supplementary Figures S3E, F**). In the analysis of transcription factor activity, we found two enriched transcription factor groups, with TFAP2C, RFX1, FOXA1 mainly enriched in CS1 and CS3, and E2F5, ATF4, RELA, JUN mainly enriched in CS2 and CS4 (**Supplementary Figure S3I**).

Furthermore, We compared the differences in clinical information among the four groups. Through the comparison of clinical information, we found that all samples in CS1 were ER-positive, while the majority in CS2 and CS4 were ER-negative. Over 90% of samples in CS2 and CS3 were HER2-negative, while 75% in CS1 and 74% in CS3 were HER2-negative. 74% of samples in CS1 were Luminal B subtype, 56% in CS3 were Her2 subtype, and over 90% in CS2 and CS4 were Basal subtype. 89% of samples in CS1 showed no response to neoadjuvant therapy, while the response was highest in CS3 at 52% (**Figures 2D–F**).In addition, we compared our subtypes with the PAM50 subtypes (104) and immune subtypes. We found that the CS1 subtype predominantly corresponds to the Luminal B subtype, while the CS2 and CS4 subtypes mainly correspond to the Basal subtype. According to immune subtyping, these samples primarily belong to the IS2 subtype (105). The IS2 subtype in the TCGA immune classification is characterized as the “IFN-γ dominant” subtype. This subtype is known for its high levels of immune activity, particularly involving interferon-gamma (IFN-γ) signaling, which plays a crucial role in the immune response to tumors. The IS2 subtype typically exhibits a robust immune response, which can influence the tumor microenvironment and potentially impact therapeutic outcomes. These results indicate significant differences and characteristics in clinical features and molecular subtypes among the four groups. CS2 and CS4 were more similar, while CS1 was more unique compared to the other three subtypes.

To further explore the differences in signaling pathways among the four subtypes, we conducted GSVA (106) analysis on transcriptome data of the four subtypes. We performed enrichment analysis using 186 classic signaling pathway gene sets from the KEGG database (107). The comparison results showed significant enrichment of oxidative phosphorylation, endocytosis, glycosylphosphatidylinositol (GPI) anchor biosynthesis pathways in CS1. Lysine degradation, ribosome, mismatch repair, RNA degradation pathways were significantly enriched in CS2. Drug metabolism cytochrome P450, steroid hormone biosynthesis, Tyrosine metabolism pathways were significantly enriched in CS3. Autoimmune thyroid disease, Viral myocarditis, Antigen processing and presentation pathways were significantly enriched in CS4 (**Figure 2I**).

We evaluated the scores of 11 gene sets in 678 samples using the EaSleR package, including CYT, TLS, IFNy, Ayers-explS, Tcell-inflamed, Roh-IS, DavoII-IS, chemokines, resF-up, resF-down, and resF. We found that the scores of immune therapy-related gene sets in the pCR-positive group were significantly higher than those in the pCR-negative group (**Supplementary Figures S4A, B**). Similarly, we found that the scores of immune therapy-related gene sets in the CS4 subtype were significantly higher than those in the other three subtypes (**Figures 2J-T**). We compared the scores of immune therapy-related gene sets between pCR-positive and pCR-negative samples in each subtype. The results showed that in CS2 and CS4 subtypes, the scores of CYT, IFNy, Tcell-inflamed, and Roh-IS immune gene sets were significantly higher in the pCR-positive group than in the pCR-negative group (Student-t Test, P<0.05; **Supplementary Figure S4C**). To further validate these results, we used the TIDE computational framework to assess the scores of 12 immune therapy-related indicators in 678 samples. We found that the positive indicators of immune therapy, including IFNG, CD274, CD8, CTL_flag, CTL, were significantly higher in the pCR-positive group than in the pCR-negative group (student-t Test, P<0.05). Conversely, the negative indicators of immune therapy, such as Exclusion and TAM M2, were significantly lower in the pCR-positive group (student-t Test, P<0.05). Similarly, we compared the scores of 12 immune therapy indicators between pCR-positive and pCR-negative samples in the four subtypes. The results indicated that in CS2 and CS4 subtypes, the positive indicators of immune therapy, IFNG, and CTL, were significantly higher in the pCR-positive group than in the pCR-negative group (Student-t test, P<0.05). Conversely, the negative indicator of immune therapy, CAF, was significantly lower in the pCR-positive group than in the pCR-negative group (Student-t test, P<0.05) (**Supplementary Figures S4D, E**). These results suggest that the immune therapy efficacy in the pCR-positive group may be better than that in the pCR-negative group. Moreover, as a highly immune-infiltrated subtype, CS4 may have better immune therapy efficacy than the other three subtypes, while the efficacy of immune therapy in the CS1 subtype may be the poorest. In the CS4 subtype, patients with pCR-positive response may have a better response to immune therapy.

Next, we used the pRRophetic package to evaluate the differences in commonly used chemotherapy drugs in four multimodal breast cancer subtypes. We found significant differences in the IC50 of chemotherapy drugs Cisplatin, Gemcitabine, Paclitaxel, and Vinorelbine among the four subtypes (Kruskal-Wallis Test). CS1 was the least sensitive to Cisplatin, while CS2 and CS4 were the most sensitive. We obtained similar results for the chemotherapy drugs Gemcitabine and Vinorelbine. Paclitaxel was most sensitive to CS3 subtype (**Figures 2U-X**). These results were consistent with the pCR response, indicating that CS1 had an unfavorable response to chemotherapy drugs. In addition, we also used oncoPredict to predict drug responses in the four subtypes within the GDSC1 and GDSC2 drug databases. We found that the CS1 subtype exhibited higher drug resistance to most drugs, followed by the CS2 subtype (**Supplementary Figure S4F**). To further accurately identify effective therapeutic drugs for the four breast cancer multi-modal subtypes, we utilized the cmap database (https://www.broadinstitute.org/connectivity-map-cmap) to analyze the highly expressed marker genes in each subtype. We found that for the CS1 subtype, the genes ESR1, CYP2B6, PGR, and SERPINA6 were the most commonly targeted drug targets. Among them, the drugs targeting ESR1 mainly had the mechanism of action (MOA) as Estrogen receptor agonist. The drugs targeting PGR had the MOA of Progesterone receptor agonist. The drugs targeting SERPINA6 had the MOA of Glucocorticoid receptor agonist. For the CS2 subtype, the primary drug target was the CDK1 gene, with the MOA label of CDK inhibitor. For the CS3 subtype, the main drug target was ERBB2, with the MOA label of EGFR inhibitor. For the CS4 subtype, the primary drug targets were MMP9 and LCK, with the MOA of Matrix metalloprotease inhibitor and SRC inhibitor, respectively. These results suggest that the four multi-modal subtypes have significantly different drug targets and mechanisms of action, providing important evidence for personalized treatment strategies for each subtype.

Through the study of CS1 subtype, we identified ESR1, CYP2B6, PGR, and SERPINA6 as critical drug targets. Drugs targeting ESR1, primarily estrogen receptor agonists, modulate breast cell proliferation and apoptosis by activating the estrogen receptor (108). PGR, as a progesterone receptor agonist, also plays a significant role in breast cancer treatment (109). Targeting SERPINA6, which acts as a glucocorticoid receptor agonist, may inhibit tumor progression by regulating stress responses and inflammatory processes (110). These findings provide multiple potential therapeutic options for patients with the CS1 subtype. For the CS2 subtype, CDK1 is the primary drug target. Inhibitors of CDK1 can arrest the cell cycle, thereby inhibiting tumor cell proliferation. CDK inhibitors have shown promising results in treating various cancers, particularly in patients resistant to conventional chemotherapy (111). In the CS3 subtype, there is a significant increase in the expression of ERBB2 (also known as HER2). Drugs targeting ERBB2, such as EGFR inhibitors, effectively block this signaling pathway, thus inhibiting tumor cell growth and proliferation (112). This discovery underscores the importance of selecting targeted therapies based on molecular characteristics in breast cancer treatment. Finally, for the CS4 subtype, MMP9 and LCK were identified as major drug targets. As a matrix metalloproteinase, MMP9 inhibitors can prevent tumor cell invasion and metastasis (113). LCK, a member of the SRC family kinases, can be targeted by inhibitors to disrupt cell signal transduction, thereby inhibiting tumor cell proliferation and survival (114).

In summary, our research successfully identified specific drug targets and mechanisms for each of the four breast cancer subtypes through CMAP database analysis. These results not only reveal the biological differences between the subtypes but also provide a scientific basis for personalized treatment strategies. Future studies should further validate the clinical efficacy of these targets and drugs to offer more precise and effective treatment options for breast cancer patients.

The TCGA database contains more comprehensive omics information. We used the NTP algorithm to predict the breast cancer multimodal subtypes of 1098 samples in the TCGA-BRCA cohort based on the transcriptome data constructed by the ISPY2 predictive model. By screening FDR<0.05 as the criterion, we finally obtained 1026 effectively predicted samples. CS1 subtype contained 462 samples, CS2 contained 138 samples, CS3 contained 159 samples, and CS4 contained 267 samples. Similarly, we conducted GSEA analysis on differentially expressed genes in each subtype (KEGG database 186 classic signaling pathways). We found that the differential genes in CS1 were mainly enriched in signaling pathways such as Glycosylphosphatidylinositol GPI Anchor Biosynthesis, Peroxisome, Aminoacyl TRNA Biosynthesis, and Oxidative Phosphorylation. Differential genes in CS2 were mainly enriched in Cell Cycle, Ribosome, DNA Replication, and Spliceosome pathways. Differential genes in CS3 were mainly enriched in metabolism-related signaling pathways such as Steroid Hormone Biosynthesis and Pentose And Glucuronate Interconversions. Differential genes in CS4 were mainly enriched in inflammation-related signaling pathways such as Graft Versus Host Disease, Allograft Rejection, and Cell Adhesion Molecules CAMs (**Supplementary Figures S6A, B**). This result is consistent with the functional enrichment results obtained from the ISPY2 cohort. Similarly, we compared the IC50 of four commonly used breast cancer chemotherapy drugs (Cisplatin, Gemcitabine, Paclitaxel, Vinorelbine) among the four breast cancer multimodal subtypes. We found that CS1 was the least sensitive to all chemotherapy drugs, while CS2 and CS4 remained the most sensitive subtypes to these four chemotherapy drugs (Kruskal-Wallis, P<0.05) (**Supplementary Figures S6C–F**). This result is consistent with the results obtained from the ISPY2 cohort. Consistent results were observed in the Metabrick BRCA cohort (**Supplementary Figures S6G–L**). This indicates that the marker-based NTP algorithm we used can correctly classify breast cancer samples in the TCGA-BRCA and Metabrick-BRCA cohorts into breast cancer subtypes.

**Figure 3:**
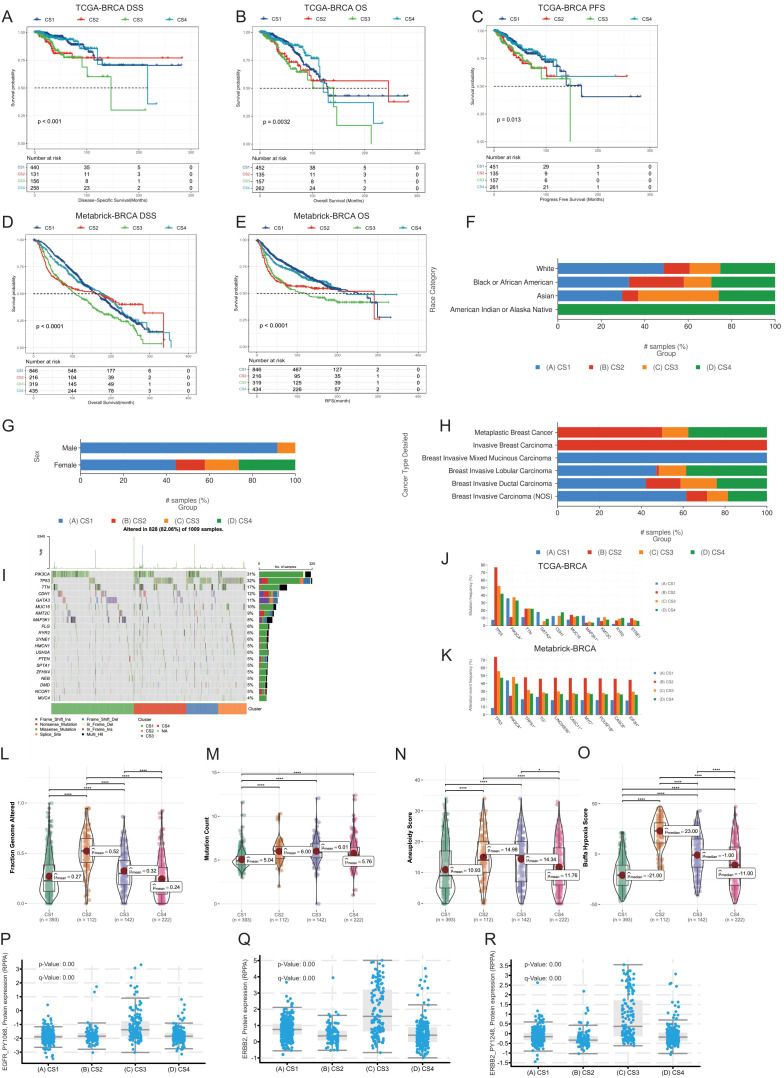
Comparative analysis of four breast cancer multimodal subtypes from multiple perspectives. **(A–E)** Prognostic information comparisons in the TCGA-BRCA and Metabric-BRCA cohorts, examining Disease-Specific Survival (DSS), Overall Survival (OS), and Progression-Free Survival (PFS). Significant prognostic differences were observed among the four breast cancer multimodal subtypes (P<0.001). **(F)** Distribution of population-specific characteristics demographics across the four subtypes in the TCGA-BRCA cohort. **(G)** Comparison of sex distribution within the four subtypes in the TCGA-BRCA cohort. **(H)** Distribution of cancer pathological subtypes within the four subtypes in the TCGA-BRCA cohort. **(I, J)** Genetic mutation comparisons among the four subtypes in the TCGA-BRCA cohort. **(K)** Genetic mutation comparison in the Metabric-BRCA cohort validating findings from the TCGA-BRCA cohort, with similar patterns observed in PIK3CA and TP53 mutation frequencies. **(L–O)** Comparative analysis of Fraction Genome Altered, Mutation Count, Aneuploidy Score, and Buffa Hypoxia Score across the four breast cancer multimodal subtypes in the TCGA-BRCA cohort. **(P–R)** Protein expression differences in RPPA data for EGFR_PY1068, ERBB2, and ERBB2_PY1248 in the TCGA-BRCA cohort. The highest expression of these proteins was noted in the CS3 subtype, consistent with findings from the ISPY2 cohort, further supporting the robustness of the multimodal subtype construction.

Next, we utilized the comprehensive multi-omics data from the TCGA-BRCA and Metabrick-BRCA cohort to validate and compare the clinical and pathological information of the breast cancer multimodal subtypes we established, thereby obtaining richer information. Through the cbioportal platform, we compared the prognosis of the four subtypes. We found that the disease-specific survival, overall survival, and Progress-Free Survival of the CS4 and CS1 subtypes were significantly better than those of the CS2 and CS3 subtypes, with significant differences in prognosis among the four subtypes (Logrank Test, P<0.05) (**Figures 3A-C**). We obtained the same results in the Metabrick-BRCA cohort as well (**Figures 3D, E**). We found that almost all samples of the CS4 subtype were from the American Indian or Alaska Native population, while the CS3 subtype had a higher proportion in the Asian population, and the CS1 subtype had a higher proportion in the Caucasian population (**Figure 3F**). Additionally, Invasive Breast Carcinoma predominantly belonged to the CS2 subtype, while Breast Invasive Mixed Mucinous Carcinoma predominantly belonged to the CS1 subtype (**Figure 3G**). In terms of sex, we found that over 90% of male breast cancers belonged to the CS1 subtype, followed by the CS3 subtype (**Figure 3H**). These findings reveal associations between reported race/ethnicity and the breast cancer multimodal subtypes, as well as an observed trend towards an association between male sex and the CS1 subtype. However, the TCGA-BRCA cohort included only 12 male breast cancer cases, limiting the statistical power to draw definitive conclusions about sex-specific differences. Subsequently, we compared the differences in genomic mutations among the four breast cancer multimodal sequencing data. We found that PIK3CA had the highest mutation rate, occurring in 31% of all subtypes, with a notably lower mutation rate in the CS2 subtype. The TP53 gene mutation, present in 32% of cases, was the second most frequent and primarily occurred in the CS2 subtype, while its mutation rate was very low in the CS1 subtype. Other significant mutations included TTN (17%), CDH1 (12%), and GATA3 (11%)(**Figure 3I**). We then compared the different mutated genes in the four groups. The mutation characteristics of the CS1 subtype were low mutations in TP53 and TTN but high mutations in GATA3 and MAP3K1. The mutation characteristics of the CS2 subtype were high mutations in TP53, with low mutation rates in PIK3CA, GATA3, and CDH1. The CS2 subtype had a higher hypoxia score and a poorer prognosis, which may be related to these factors (**Figure 3J**). We obtained similar results in the Metabrick-BRCA cohort (**Figure 3K**). Additionally, we validated the clinical characteristics comparison across the four multimodal subtypes within this cohort (**Supplementary Figure S7D–K**). Through comparison on cbioportal, we found that TP53 mutations were mainly enriched in the CS2 subtype, GATA3 and MAP3K1 were mainly enriched in the CS1 subtype, while PIK3CA and RICTOR were mainly enriched in the CS3 subtype (**Supplementary Figure S7A**).We compared the Fraction Genome Altered and Mutation Count. We found that the CS2 subtype had the highest genomic mutation score, while the CS1 and CS4 subtypes had the lowest. The CS1 subtype had the lowest mutation burden, which may be related to the lower TP53 mutation rate in the CS1 subtype (**Figures 3L, M**). Furthermore, the Aneuploidy Score and Buffa Hypoxia Score were significantly lower in the CS1 and CS4 subtypes than in the CS2 and CS3 subtypes (t-test, P<0.05) (**Figure 3N, O**). The hypoxia score of the CS1 subtype was the lowest among all subtypes, while that of the CS2 subtype was the highest. Considering the active oxidative phosphorylation signaling pathway in the CS1 subtype and the active inflammation-related signaling pathway in the CS4 subtype, we speculate that the tumor mutational burden is low in the CS1 subtype, and the oxidative phosphorylation activity is good, with low activity in hypoxia-related signaling pathways, which may be related to the state of tumor cells. The tumor mutational burden is low in the CS4 subtype, with high activity in inflammation-related signaling pathways and low activity in hypoxia-related signaling pathways, which may be related to the transportation of immune cells and oxygen in newly formed blood vessels within the tumor tissue. Although the prognosis of the CS1 and CS4 subtypes is better, the mechanisms behind them are completely different. The CS2 subtype has a high tumor mutation rate, while the activity of hypoxia-related signaling pathways is low, resulting in a poorer prognosis. The CS3 subtype may be related to other factors.

We compared the expression levels of EGFR_PY1068, ERBB2, and ERBB2_PY1248 in the four multimodal subtypes using TCGA-BRCA RPPA protein data (**Figures 3P–R**). Our analysis revealed that these three proteins are predominantly expressed in the CS3 subtype, validating the protein data results obtained from the ISPY2-BRCA cohort. This further confirms the reliability of our subtype classification. Furthermore, we compared the characteristics of these subtypes across multiple modalities in the TCGA-BRCA cohort. We compared the gene-level methylation characteristics of the four breast cancer multimodal subtypes. In the CS1 subtype, genes highly methylated included SLC43A3, MMP7, and MIA. In the CS2 subtype, genes highly methylated included NAV1, MUC1, PRR18, and TBX19. In the CS3 subtype, genes highly methylated included RUSC2, CYBA, and SOCS3. In the CS4 subtype, genes highly methylated included AGR2, TGFB3, NOR2, and ANKRD9 (**Supplementary Figure S8A**). Highly methylated genes in the CS1 subtype were mainly enriched in signaling pathways such as Cytokine-cytokine receptor interaction, Neuroactive ligand-receptor interaction, Calcium signaling pathway, and cAMP signaling pathway. On the other hand, lowly methylated genes in the CS1 subtype were mainly enriched in signaling pathways such as Tight junction, Hippo signaling pathway, and Primary bile acid biosynthesis. Highly methylated genes in the CS2 subtype were mainly enriched in signaling pathways such as Allograft rejection, Autoimmune thyroid disease, and Graft-versus-host disease. This result indicates that inflammation-related signaling pathways in the CS2 subtype are inhibited by methylation at the DNA level. Lowly methylated genes in the CS2 subtype were mainly enriched in signaling pathways such as PI3K-Akt signaling pathway and MAPK signaling pathway. Considering the mutations in PIK3CA and MAP3K1 in the CS2 subtype, these cell proliferation and differentiation-related signaling pathways are activated at the DNA level (115). Highly methylated genes in the CS3 subtype were mainly enriched in signaling pathways such as cAMP signaling pathway, Human T-cell leukemia virus 1 infection, and Cellular senescence, indicating that energy metabolism and inflammation-related signaling pathways are inactivated in the CS3 subtype, which is completely different from the CS2 and CS4 subtypes. In the CS4 subtype, genes related to the PI3K-Akt signaling pathway and MAPK signaling pathway were in a highly methylated inactive state, while lowly methylated genes were mainly enriched in signaling pathways such as Cytokine-cytokine receptor interaction, Calcium signaling pathway, cAMP signaling pathway, and Human T-cell leukemia virus 1 infection. These results indicate that energy metabolism and inflammation-related signaling pathways in CS4 are in a methylated activated state (**Supplementary Figure S8B**). These results demonstrate significant differences in methylation levels among the breast cancer multimodal subtypes we proposed, and these differences can be mutually reflected by other omics information. Additionally, we compared the protein-level differences among the four multi-modal subtypes. In the CS1 subtype, the highly expressed proteins included PGR (progesterone receptor), LYRM9, and TCEAL1. The high expression of PGR may indicate the subtype’s specificity in hormonal responses (116). In the CS2 subtype, the highly expressed proteins were mainly TRIM29, TSPYL5, and S100A1. TRIM29 is a protein involved in cell proliferation and DNA damage repair (117), TSPYL5 plays an important role in cell cycle regulation (118) in the CS2 subtype. In the CS3 subtype, the highly expressed proteins included ERBB2 (also known as HER2), HMGCS2, and PRODH. ERBB2 is a known oncogene that is often overexpressed in breast cancer, and its high expression may indicate that the CS3 subtype has a higher proliferative potential (119). HMGCS2 is involved in ketone body formation (120), and PRODH is related to amino acid metabolism (121), suggesting that the CS3 subtype may have unique metabolic characteristics. In the CS4 subtype, the enriched proteins were FBP2, HLA-F, and CORO1A. FBP2 is a glycolytic enzyme involved in the regulation of glucose metabolism (122). HLA-F participates in the regulation of immune responses (123), and CORO1A affects the reorganization of the cytoskeleton (124), indicating that the CS4 subtype may have unique biological characteristics in metabolism and immune response. These proteins are consistent with the protein results from the ISPY-2 BRCA cohort (**Supplementary Figure S8D, E**). We found that the majority of methylated genes were under methylation control. For example, the expression level of MMP7 was significantly negatively correlated with the methylation level of MMP7 (Spearman cor.=-0.44, P<0.001), and the expression level of MUC1 was also significantly negatively correlated with its methylation level (Spearman cor.=-0.4, P<0.001) (**Supplementary Figure S8F, G, H**). Finally, we found that in the comparison of intratumoral microbiota, the four multi-modal subtypes also exhibited significant differences. The CS1 subtype was enriched with microbes such as Bafinivirus, Psychrilyobacter, and Simplexvirus, the CS2 subtype was enriched with Bacteriovorax, Hypovirus, and Methanocella, the CS3 subtype was enriched with Candidatus-Microthrix, Phenylobacterium, and Rubrobacterium, and the CS4 subtype was enriched with Carboxydothemus, Anoxybacillus, and Anaeromusa (**Supplementary Figure S8H**). These results all suggest the unique biological characteristics of the four subtypes.

### 3.3 At single-cell resolution, breast cancer neoadjuvant therapy subtypes show distinct tumor microenvironment characteristics

**Figure 4:**
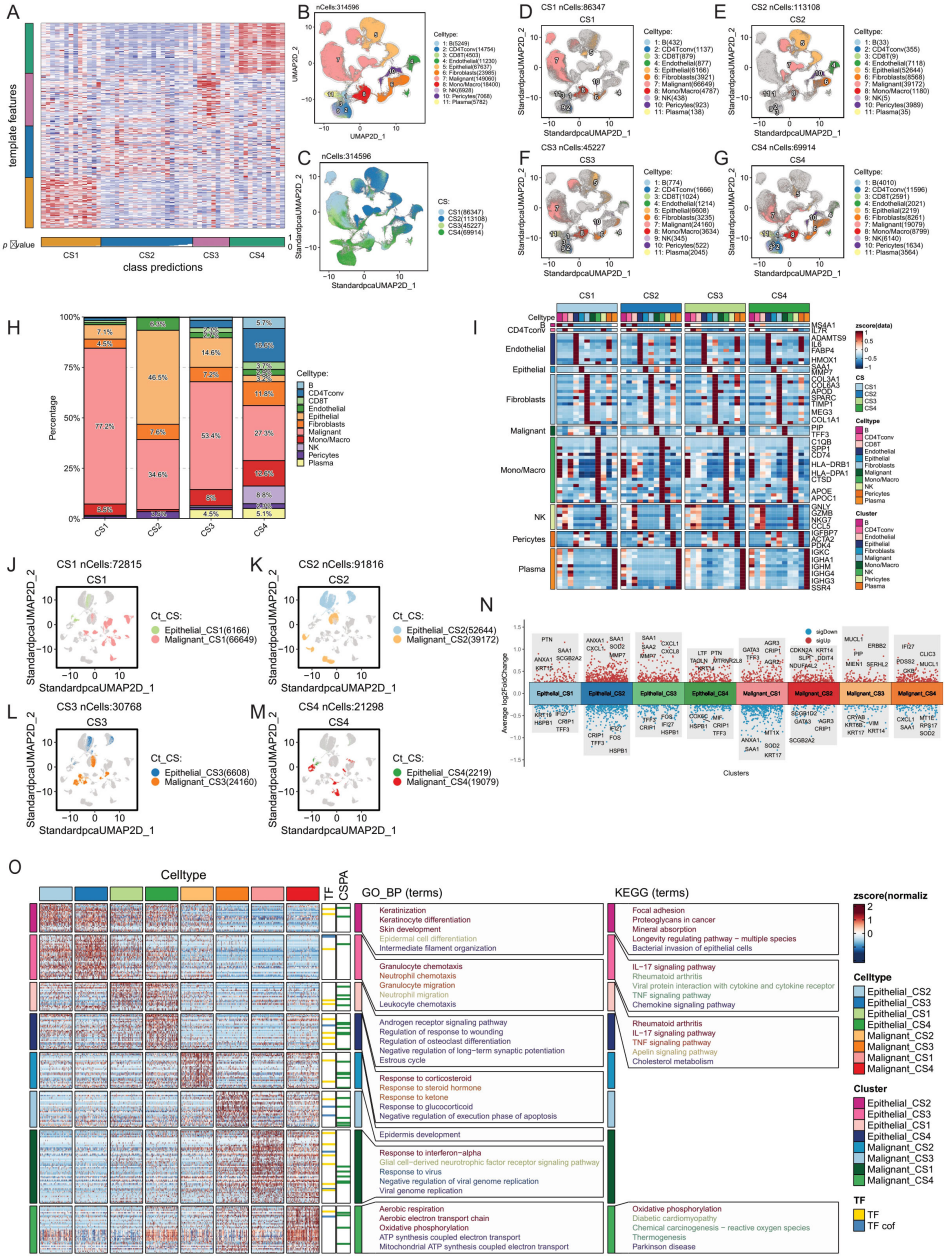
Tumor microenvironment comparison of four breast cancer multimodal subtypes at the single-cell level. **(A)** The NTP algorithm was applied to infer the breast cancer multimodal subtypes from 53 single-cell sequencing datasets of breast cancer. **(B)** A total of 314,596 cells were classified into 11 cell types: 5,249 B cells, 14,754 CD4 T conv cells, 4,503 CD8 T cells, 11,230 endothelial cells, 67,637 epithelial cells, 23,985 fibroblasts, 149,060 malignant cells, 18,400 monocytes/macrophages, 6,928 NK cells, 7,068 pericytes, and 5,782 plasma cells. **(C)** UMAP visualization displaying the distribution of cells from the four breast cancer multimodal subtypes. **(D)** CS1 subtype contained 86,347 cells, including 432 B cells, 1,137 CD4 T conv cells, 879 CD8 T cells, 877 endothelial cells, 6,166 epithelial cells, 3,921 fibroblasts, 66,649 malignant cells, 4,787 monocytes/macrophages, 438 NK cells, 923 pericytes, and 138 plasma cells. **(E)** CS2 subtype comprised 113,108 cells, including 33 B cells, 355 CD4 T conv cells, 9 CD8 T cells, 7,118 endothelial cells, 52,644 epithelial cells, 8,568 fibroblasts, 39,172 malignant cells, 1,180 monocytes/macrophages, 5 NK cells, 3,989 pericytes, and 35 plasma cells. This subtype showed a marked reduction in immune cells compared to the others. **(F)** CS3 subtype consisted of 45,227 cells, including 774 B cells, 1,666 CD4 T conv cells, 1,024 CD8 T cells, 1,214 endothelial cells, 6,608 epithelial cells, 3,235 fibroblasts, 24,160 malignant cells, 3,634 monocytes/macrophages, 345 NK cells, 522 pericytes, and 2,045 plasma cells. **(G)** CS4 subtype included 69,914 cells, with 4,010 B cells, 11,596 CD4 T conv cells, 2,591 CD8 T cells, 2,021 endothelial cells, 2,219 epithelial cells, 8,261 fibroblasts, 19,079 malignant cells, 8,799 monocytes/macrophages, 6,140 NK cells, 1,634 pericytes, and 3,564 plasma cells. This subtype had the highest abundance of immune cells. **(H)** Percentage distribution of 11 cell types within the tumor microenvironment across the four breast cancer multimodal subtypes. **(I)** Gene markers for 11 cell types within the four multimodal subtypes. The markers were consistent across subtypes, with B cell markers including MS4A1, CD4 T conv cell markers such as IL7R, and endothelial cell markers including ADAMTS9, IL6, FABP4, and HMOX1. Epithelial cell markers included SAA1 and MMP7, while fibroblast markers included COL3A1, COL6A3, APOD, and COL1A1. Malignant cell markers were PIP and TFF3, monocyte/macrophage markers included C1QB, APOE, CTSD, and CD74. NK cell markers were GNLY, NKG7, and GZMB, pericyte markers were IGFBP7, ACTA2, and PDK4, and plasma cell markers were IGKC, IGHA1, and IGHM. **(J–M)** UMAP plots showing distinct distribution patterns of the four multimodal subtypes, highlighting clear differences in cell composition. Cells were classified into eight groups based on multimodal subtype and cell type: Epithelial_CS1, Epithelial_CS2, Epithelial_CS3, Epithelial_CS4, Malignant_CS1, Malignant_CS2, Malignant_CS3, Malignant_CS4. **(N)** Volcano plots depicting differential expression analysis of the eight cell groups (Wilcox-Test). **(O)** Heatmap of differential expression and the results of transcription factor (TF), transcriptional cofactor (CSPA), GO_BP pathway, and KEGG pathway enrichment analyses for the eight cell groups.These analyses provide an in-depth view of the tumor microenvironment at the single-cell level, revealing significant heterogeneity in cellular composition, gene expression, and pathway activation among the four breast cancer multimodal subtypes.

We aggregated pseudo-bulk RNA information from 53 single-cell sequencing samples of the GSE161529 dataset. Using the NTP algorithm on the signature gene matrix of the four subtypes from the ISPY2 dataset, we screened samples with FDR < 0.05 as statistically significant subtype samples, ultimately obtaining subtype information for 49 samples (**Figure 4A**). Among these, there were 13 samples in the CS1 subtype, 16 in CS2, 8 in CS3, and 12 in CS4. After proper subtyping, we proceeded to compare and analyze the single-cell sequencing data of these subtypes.

Firstly, leveraging cell annotation information from the TISCH2 database, we obtained the quantities of 11 major cell types (B, CD4Tconv, CD8T, Endothelial, Epithelial, Fibroblasts, Malignant, Mono/Macro, NK, Pericytes, Plasma) in the four breast cancer multimodal subtypes. CS1 had 86,347 cells, CS2 had 113,108 cells, CS3 had 45,227 cells, and CS4 had 69,914 cells (**Figures 4B, C**). We compared the distribution of the 11 major cell types in the 4 subtypes and found that malignant cells accounted for 77.2% in CS1, 34.6% in CS2, 53.4% in CS3, and 27.3% in CS4. Furthermore, we observed notable differences in other cell types among the subtypes, such as the high proportion of NK cells in CS4 and the presence of Plasma cells in CS3 and CS4, indicating enriched immune activation responses in these subtypes. Additionally, B cells and CD4Tconv were highly represented in CS4, suggesting the formation of relatively more tertiary lymphoid structures, which contribute to the anti-tumor immune response (**Figures 4D–G, H**) (125, 126). We performed differential analysis and functional enrichment analysis on these 11 major cell types, and found that the marker genes and functions of these cell types were consistent with previous studies (**Figure 4I**). The results highlight significant heterogeneity in the tumor microenvironment among the four neoadjuvant therapy subtypes, consistent with bulk-RNAseq analysis findings.

Next, we focused on exploring the differences in Malignant and Epithelial cells among the four subtypes. Firstly, we observed distinct distributions of Malignant and Epithelial cells from the four breast cancer multimodal subtypes in different regions of the UMAP scatter plot, accompanied by significant differences in transcription factors and gene functions (**Figures 4J-M**). Specifically, Epithelial cells in each subtype showed enrichment in different signaling pathways, reflecting distinct biological characteristics. Notably, Epithelial cells in CS4 exhibited pronounced inflammatory features, with significant enrichment in the Antigen processing and presentation, Allograft rejection, and Viral myocarditis signaling pathways. Furthermore, IL-17 signaling pathway was significantly enriched in Epithelial cells across all four subtypes. However, differences in Malignant cells among the four subtypes were relatively minor (**Figures 4N-R**).

**Figure 5:**
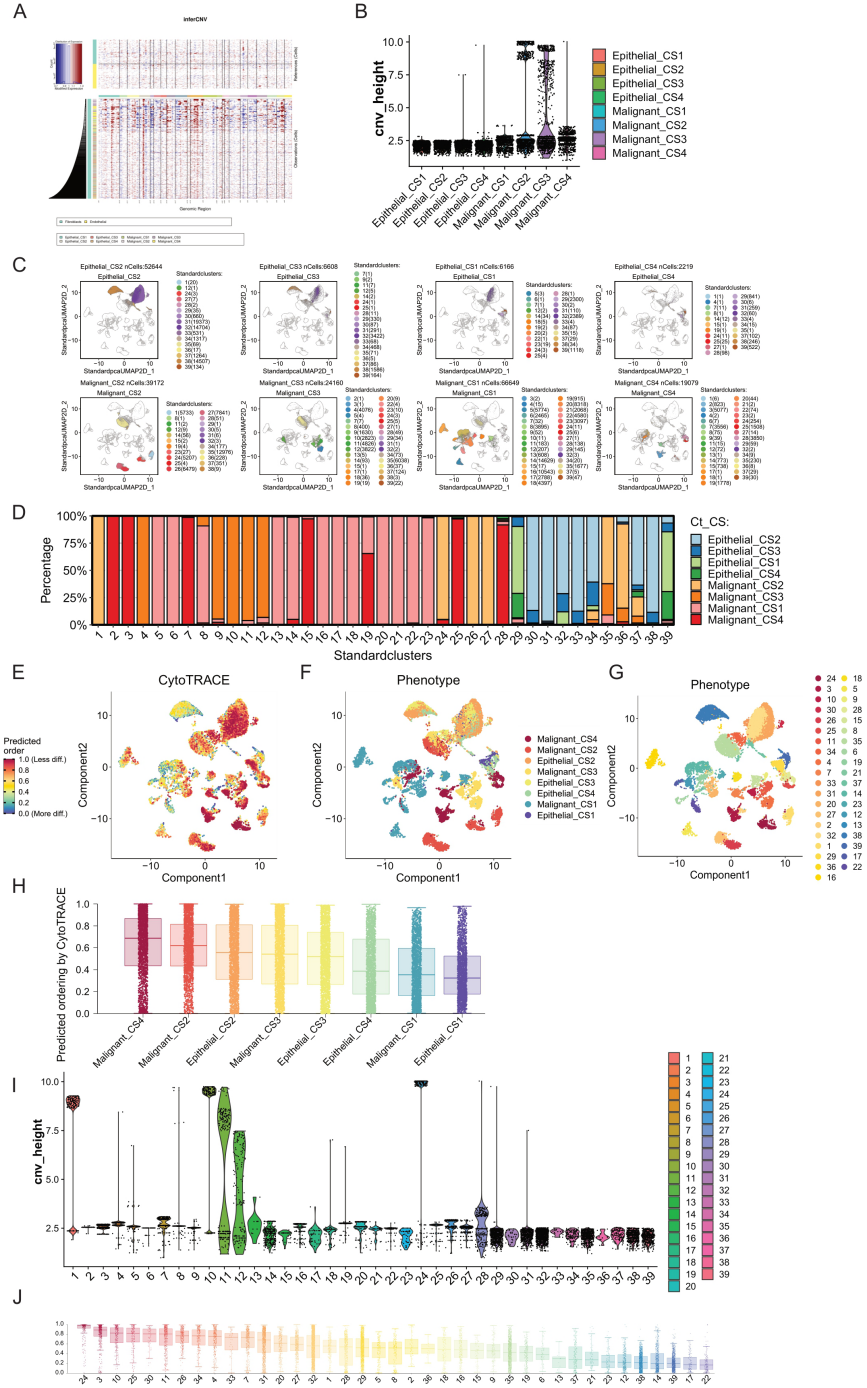
Comparison of tumor progression trajectories in breast cancer multimodal subtypes. **(A)** Copy number variation (CNV) inference in epithelial and malignant cells using inferCNV, with fibroblasts and endothelial cells serving as reference cells. **(B)** Clustering of CNVs to obtain hierarchy height (cnv_height) for each cell type. By comparing the cnv_height of the eight cell subgroups (Epithelial_CS1, Epithelial_CS2, Epithelial_CS3, Epithelial_CS4, Malignant_CS1, Malignant_CS2, Malignant_CS3, and Malignant_CS4), we assessed the evolutionary degree of each subgroup. Malignant_CS2 and Malignant_CS3 exhibited higher tumor heterogeneity and progression. **(C)** Unsupervised clustering using the Louvain algorithm identified 39 cell clusters from epithelial and malignant cells, revealing distinct distribution patterns across the eight cell subgroups. **(D)** Distribution of the 39 cell clusters within the eight cell subgroups. **(E)** Differentiation degree of epithelial and malignant cells assessed using CytoTRACE, visualized in a differentiation heatmap. Higher differentiation levels indicate more mature tumor cell differentiation trajectories. **(F)** Distribution of the eight cell subgroups in the differentiation heatmap. **(G)** Distribution of the 39 cell clusters in the differentiation heatmap. **(H)** Ranking of the differentiation degrees of the eight cell subgroups predicted by CytoTRACE, showing that Malignant_CS4 had the highest differentiation, while Epithelial_CS1 had the lowest. **(I)** Comparison of cnv_height across the 39 cell clusters, highlighting clusters 1, 10, and 24 as having high differentiation degrees. **(J)** Ranking of the differentiation degrees of the 39 cell clusters predicted by CytoTRACE, indicating that cluster 24 (mainly from Malignant_CS2) had the highest differentiation degree, followed by cluster 3 (from Malignant_CS4), despite cluster 3 not having particularly high cnv_height. The lowest differentiation levels were observed in clusters 17 and 22, both from Malignant_CS1, suggesting that Malignant_CS1 tumor cells are in a low differentiation state, potentially related to drug resistance. These analyses offer insights into the tumor progression and differentiation trajectories of breast cancer multimodal subtypes, emphasizing the heterogeneity and distinct evolutionary pathways within and between subtypes. The findings highlight the variability in tumor cell differentiation states, with potential implications for understanding drug resistance and therapeutic responses.

To further explore the differences between Epithelial cells and Malignant cells in these subtypes, we conducted copy number variation (CNV) analysis on these two cell types across the four subtypes. Using infercnv, we performed analysis in two modes (with the parameter cluster_by_groups set to TRUE and FALSE, with Fibroblasts and Endothelial cells as references). We compared the CNVs of Epithelial cells and Malignant cells across the four subtypes and found that regardless of the subtyping method, the CNVs of Malignant cells were significantly higher than those of Epithelial cells (**Figure 5A** **;** **Supplementary Figure S10A**). This indicates that our cell annotation was correct and suggests that Malignant cells have a higher degree of genomic variation.

By conducting hierarchical clustering on all cells, with the height of each cell in the clustering tree indicating its differentiation level, we observed that Malignant cells exhibited a much higher degree of differentiation compared to Epithelial cells(**Figure 5B**). Additionally, Malignant cells from CS2 and CS3 exhibited the highest differentiation levels, followed by those from CS4, while Malignant cells from CS4 showed the lowest differentiation level. Furthermore, Malignant cells from CS2 and CS3 formed distinct clusters with significant differentiation.

Therefore, to further understand the heterogeneity of these cell subtypes, we performed unsupervised clustering (louvain cluster) on Epithelial cells and Malignant cells, reclassifying them into 39 subgroups(**Figure 5C**). We found that highly differentiated clusters, such as cluster 1 and cluster 24, primarily comprised subgroups of Malignant_CS2, while cluster 10 mainly consisted of subgroups of Malignant_CS3. Conversely, relatively less differentiated cluster 28 predominantly belonged to Malignant_CS4 subgroups (**Figure 5D**). We successfully identified highly differentiated subgroups of Malignant cells within the four breast cancer multimodal subtypes. These subgroups did not overlap, indicating significant tumor evolutionary differences among these subtypes.

Breast cancer malignant cells originate from epithelial cell lesions. Therefore, we further explored the evolution of Epithelial and Malignant cells across these four multimodal subtypes of breast cancer. Initially, we assessed the differentiation process of these cells using Cytotrace (**Figures 5E-G**). Firstly, we observed that Epithelial_CS1 exhibited the lowest degree of differentiation, followed by Malignant_CS1. This suggests that the malignant cells in CS1 breast cancer exhibit a state close to that of normal breast epithelial cells. Additionally, we found that Malignant_CS4 displayed the highest degree of differentiation, followed by Malignant_CS. This finding aligns with the results obtained from copy number variation analysis. We also observed that the differentiation level of Epithelial_CS2 was higher than that of Malignant_CS3, possibly due to the high mutation characteristics of CS2 tumors (**Figure 5H**).

Furthermore, we sorted and compared the 39 cell subgroups of Epithelial and Malignant cells based on the pseudotime constructed by Cytotrace (**Figures 5I, J**). We found that cluster 24, belonging to Malignant_CS2, exhibited the highest differentiation level. Next was cluster 3, belonging to Malignant_CS4, although its position on the dendrogram was not highly ranked, indicating a potential difference in differentiation direction from malignant cells. Moreover, cluster 10, primarily composed of Malignant_CS3, ranked third in terms of differentiation status, with relatively high levels of copy variation. The differentiation direction of malignant tumor cells is not fixed. The high immune infiltration in CS4 may exert strong selective pressure, leading to the elimination of the most highly malignant cells and a relative enrichment of cells with lower genomic instability or with adaptations for immune evasion. The high differentiation level observed in CS4 malignant cells, despite the intense immune pressure, is consistent with the superior prognosis observed for this subtype. This suggests that the immune system may be effectively controlling or eliminating the most aggressive cells, or that the remaining malignant cells have adapted to the immune pressure in a way that reduces their aggressiveness.

**Figure 6:**
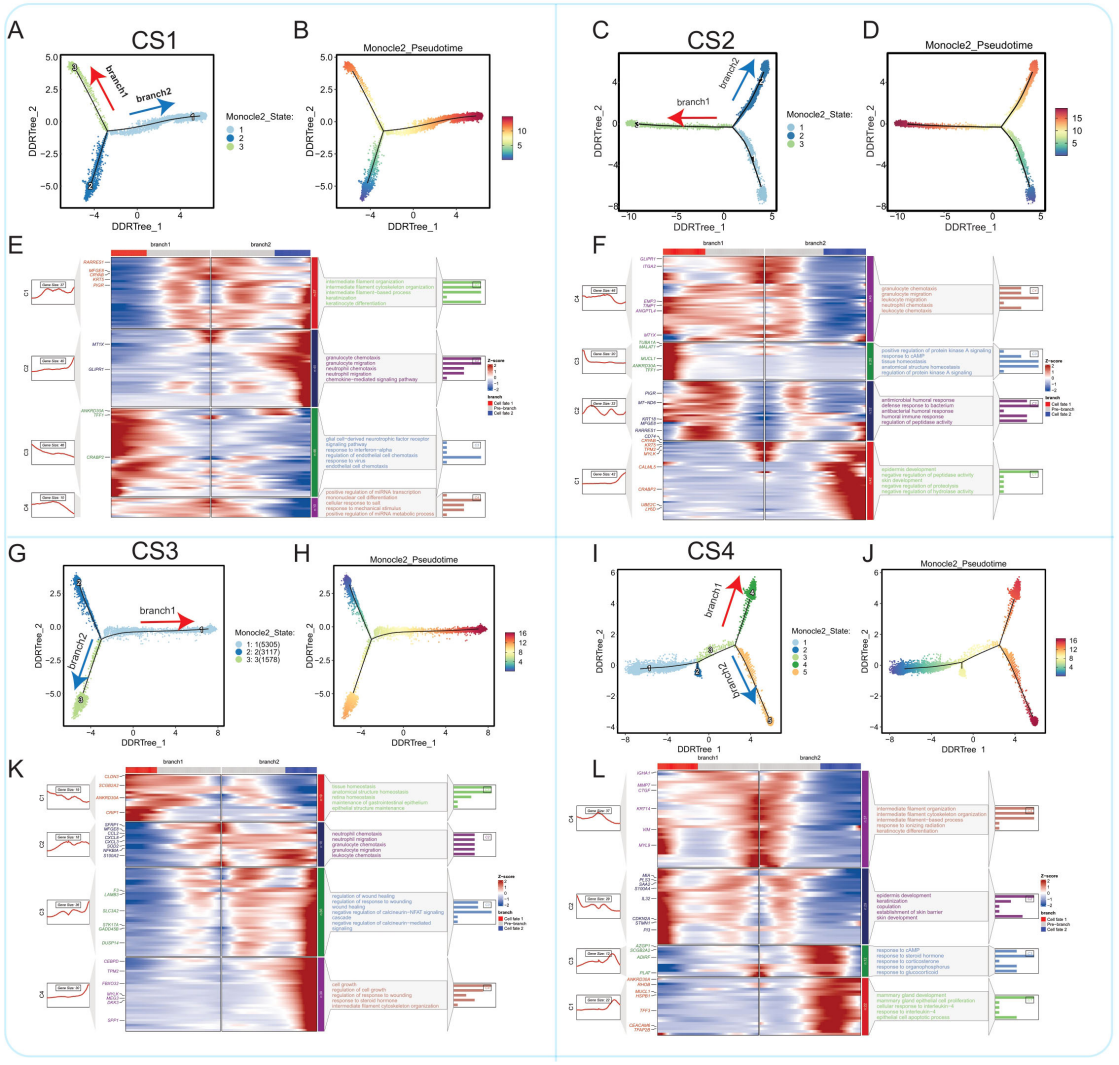
Comparison of epithelial cell malignant progression trajectories in four breast cancer multimodal subtypes. **(A)** The progression pathway of epithelial cells to malignant cells in the CS1 subtype. **(B)** In the CS1 subtype, two differentiation branches (branch1 and branch2) were identified, with branch2 showing a higher degree of differentiation compared to branch1. **(C)** The progression pathway of epithelial cells to malignant cells in the CS2 subtype. **(D)** In the CS2 subtype, two differentiation branches (branch1 and branch2) were identified, with branch1 exhibiting a higher degree of differentiation compared to branch2. **(E)** In the CS1 subtype, branch1 differentiation is predominantly regulated by genes such as ANKRD30A, TFF1, and CRABP2, and is enriched in pathways like response to interferon-alpha and endothelial cell chemotaxis. Branch2 is mainly regulated by genes like MT1X and GLIPR1, influencing pathways such as granulocyte chemotaxis, neutrophil migration, and neutrophil chemotaxis. **(F)** In the CS2 subtype, branch1 differentiation is largely governed by inflammation-related signaling pathways. Branch2 is primarily regulated by genes such as CRYAB, KRT5, and CRABP2, focusing on pathways related to epidermis development and skin development. **(G)** The malignant differentiation trajectory in the CS3 subtype. **(H)** In the CS3 subtype, branch1 displays a higher degree of differentiation compared to branch2. **(I)** The differentiation along branch1 in the CS3 subtype is regulated by genes like CLDN3, ANKRD30A, and SCGB2A2, which are enriched in pathways such as tissue homeostasis and anatomical structure homeostasis. Branch2 is primarily influenced by inflammation-related pathways. **(J)** The progression pathway of epithelial cells to malignant cells in the CS4 subtype. **(K)** In the CS4 subtype, two differentiation branches (branch1 and branch2) were identified, with branch2 showing a higher degree of differentiation. **(L)** In the CS4 subtype, branch1 differentiation is regulated by genes such as MIA, PLS3, S100A4, CDKN2A, and STMN1, and is enriched in pathways related to epidermis development and keratinization. Branch2 is primarily regulated by pathways such as response to cAMP and response to steroid hormone.

To further elucidate the evolutionary processes of malignant cells within each subtype, we performed pseudotime analysis using Monocle2 on the four multimodal breast cancer subtypes individually (**Figure 6**). CS1 Subtype: The CS1 subtype exhibited a relatively low differentiation level, consistent with its lower mutation burden and minimal differences between ‘Epithelial’ and ‘Malignant’ cells as defined by the TISCH2 annotations (**Figures 6A, B, E** **;** **Supplementary Figures S10C–E**). Two main differentiation trajectories were observed, with branch 2 showing a slightly higher degree of differentiation. Key gene modules activated during this transition were associated with fundamental biological processes such as cell signaling and cell cycle regulation, suggesting potential therapeutic targets. The limited differentiation and relative homogeneity of CS1 malignant cells may be influenced by the lower immune infiltration and predominance of signals associated with a less aggressive phenotype, as observed in our previous analyses. This suggests that CS1 tumors may maintain malignancy through relatively conserved genetic mechanisms rather than extensive genomic alterations. CS2 Subtype: In contrast to CS1, the CS2 subtype displayed a higher degree of differentiation and two distinct trajectories, with branch 1 showing greater differentiation and enrichment for pathways related to endothelial cell chemotaxis and angiogenesis (**Figures 6C, D, F** **;** **Supplementary Figures S10F–H**). This, coupled with the previously observed HGF signaling from fibroblasts in this subtype, suggests that the CS2 TME actively promotes an invasive and angiogenic phenotype. The distinct differentiation trajectories likely reflect adaptation to different microenvironmental pressures, leading to increased tumor adaptability and invasiveness. The CytoTRACE analysis indicated a higher differentiation score, suggesting a trajectory towards a more dedifferentiated and aggressive phenotype. This is consistent with the high genomic instability (high mutation rate and CNVs) observed in this subtype. While some features of epithelial differentiation may be retained, the overall trajectory, coupled with weakened cell-cell communication, likely contributes to the poorer prognosis observed for CS2. CS3 Subtype: The CS3 subtype also exhibited two main differentiation trajectories, with branch 1 showing higher differentiation and enrichment for pathways involved in tissue homeostasis and maintenance of tissue structure (**Figures 6G, H, K**; **Supplementary Figures S10I–K**). This, combined with the observed intermediate levels of immune infiltration and distinct metabolic characteristics, suggests that CS3 malignant cells may maintain their malignant phenotype through mechanisms related to cell adhesion, polarity, and cell cycle regulation. The previously noted high mutation characteristics in CS3 may be associated with its copy number variation burden, contributing to intratumoral heterogeneity and potential therapeutic resistance. The Monocle2 analysis revealed differentiation trajectories enriched for pathways involved in tissue homeostasis. However, this does not necessarily imply a less aggressive phenotype. The combination of these differentiation pathways with the observed high mutation characteristics and intermediate immune infiltration likely contributes to a complex phenotype with a poorer prognosis compared to CS1 or CS4. CS4 Subtype: The CS4 subtype, characterized by high immune infiltration, displayed the highest degree of malignant cell differentiation and two main trajectories, with branch 1 exhibiting greater differentiation (**Figures 6I, J, L**; **Supplementary Figures S10L–N**). Key gene modules were associated with epidermal development, keratinization, and cell apoptosis. This suggests that the strong selective pressure exerted by the immune system drives the evolution of malignant cells with specialized mechanisms for both maintaining malignancy and evading immune surveillance.

The pseudotime analysis reveals distinct evolutionary trajectories of malignant cells across the four multimodal breast cancer subtypes, alongside evidence of shared pathways like cell cycle regulation; these differences, driven by the interplay between intrinsic cellular programs and the selective pressures of the tumor microenvironment, highlight potential targets for both subtype-specific and broader therapeutic interventions.

Next, we analyzed the differences in transcription factor activity between epithelial cells and malignant cells of the four subtypes using SCENIC (**Supplementary Figure S10B**). By screening differentially activated transcription factors, we found similarities in the spectrum of differentially activated transcription factors between Epithelial_CS1 and Epithelial_CS4, as well as between Epithelial_CS2 and Epithelial_CS3. Commonly activated transcription factors included CD59 and FOSL1 in epithelial cells and RAD21-extended(57g) in Malignant_CS4, indicating extensive differences in transcription factor activity between the four multimodal subtypes. To further understand the role of these differentially activated transcription factors in cell malignant transformation, we performed functional analysis of key transcription factors such as CD59, FOSL1, RAD21-extended(57g), TFDP1(33g), and TFDP1-extended(64g). The activation status of these transcription factors in different subtypes may directly affect cell growth, differentiation, and malignant transformation processes. CD59 is an immune inhibitor that protects cells from complement attack. Its activation in both normal and malignant cells may regulate immune evasion and facilitate transformation (127). FOSL1 is an AP-1 transcription factor that regulates proliferation, apoptosis, and differentiation. FOSL1 activation in epithelial cells across subtypes may maintain normal epithelial cell function (128)s. RAD21 is a key component of the cohesin complex, which plays a critical role in maintaining chromosomal structural stability (129). The significant activation of RAD21-extended(57g) in Malignant_CS4 may reflect increased chromosomal instability, a common feature of many cancers. TFDP1 (transcription factor Dp-1) is a member of the transcription factor family (130). As an auxiliary factor of E2F transcription factors, TFDP1 regulates the cell cycle process. The significant activation of TFDP1(33g) and TFDP1-extended(64g) in Malignant_CS2 may indicate issues in cell cycle regulation, thereby promoting malignant transformation.

In summary, our study reveals differences in transcription factor activity between epithelial cells and malignant cells of different subtypes, which may directly impact the processes of cell growth, differentiation, and malignant transformation. Further research will help deepen our understanding of the roles of these transcription factors in cellular malignant transformation, providing new insights for cancer treatment and prevention.

### 3.4 The cell-cell communication shows significant differences among the four breast cancer molecular subtypes

**Figure 7:**
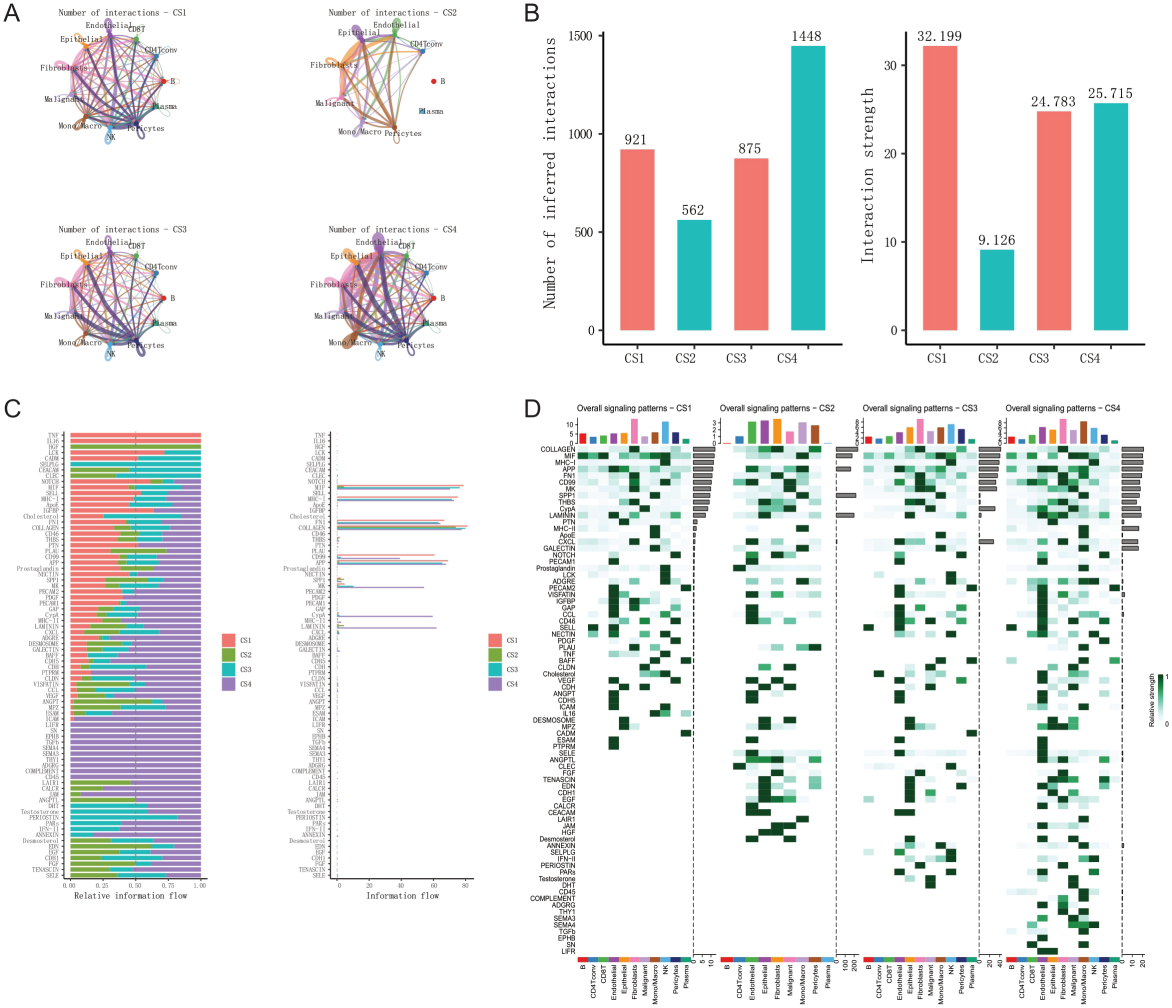
Comparison of cell-cell communication in the tumor microenvironment across four breast cancer multimodal subtypes. **(A)** Comparison of the number of interactions among 11 different cell types in each of the four breast cancer multimodal subtypes. **(B)** Comparison of the total number and strength of communications in the four breast cancer multimodal subtypes. **(C)** Comparison of the relative (left) and absolute (right) information flow of receptor-ligand pairs that show statistically significant differences among the four breast cancer multimodal subtypes. **(D)** Comparison of overall signaling patterns among the 11 cell types across the four breast cancer multimodal subtypes. The results indicate that the CS1 and CS3 subtypes exhibit the fewest cell-cell communications, whereas the CS4 subtype shows the highest level of cell-cell communications. These analyses provide a comprehensive overview of the cellular communication landscapes in the tumor microenvironments of different breast cancer multimodal subtypes. The comparative analysis highlights significant variations in the number and intensity of cell-cell interactions, as well as in the signaling pathways mediated by receptor-ligand pairs. These differences underscore the unique microenvironmental dynamics and potential intercellular regulatory mechanisms that may influence tumor progression and therapeutic response in each subtype. Understanding these communication networks could offer novel insights into targeted therapies and strategies for disrupting key signaling pathways in breast cancer treatment.

Through CellChatV2.1.2 analysis of single-cell sequencing data from four molecular subtypes, we annotated all cells into different cell types, including Endothelial, Epithelial, Fibroblasts, Malignant, Mono/Macro, NK, Pericytes, Plasma, B, CD4Tconv, and CD8T cells. First, we compared the differences in the number of cell-cell interactions among the four molecular subtypes (**Figure 7A**). Interestingly, we found that in the CS2 subtype, NK cells, B cells, and Plasma cells had almost no interactions with other cell types, indicating their communication activity was remarkably low in this subtype compared to the other subtypes. In the CS4 subtype, Mono/Macro cells and Pericytes showed significantly higher numbers of interactions with other cell types, suggesting these cells may play a more active role in this subtype. In terms of the overall cell-cell interaction counts, we also found that the CS1 subtype had the highest interaction strength, potentially indicating a relatively more active cell communication in the tumor microenvironment of this subtype. The CS4 subtype had the highest number of cell-cell interactions, revealing a higher frequency of cell-cell communication in this subtype. In contrast, the CS2 subtype exhibited the lowest cell-cell interaction counts and interaction strength, which is consistent with its poorer prognosis.

The interaction number and intensity between cells of the CS3 subtype are also relatively low, leading to a similarly poor prognosis. We observed a significant reduction in the amount and strength of cell-cell communication in the CS2 and CS3 subtypes (**Figure 7B**). We refer to this as a “weakening of intercellular communication.” Biologically, this could reflect several scenarios: fewer physical interactions between cells due to altered tumor architecture or reduced immune cell infiltration; lower expression of genes encoding key ligands or receptors; or the presence of factors that inhibit ligand-receptor binding. Given the poor prognosis associated with these two subtypes, we hypothesize that the weakening of intercellular communication may be an important factor contributing to the poor outcomes in these cancer patients.The complexity and activity of cell-cell communication within the tumor microenvironment may have a significant impact on cancer progression and prognosis. Specifically, the lack of effective intercellular communication could lead to increased immune evasion, tumor proliferation, and metastasis - the hallmarks of malignant behavior - within the tumor microenvironment. The impaired cellular crosstalk observed in the CS2 and CS3 subtypes suggests that the integrity and robustness of the communication network within the tumor milieu is a critical determinant of clinical outcomes. When this intercellular communication is disrupted, the tumor may be able to more readily evade immune surveillance, proliferate uncontrollably, and spread to distant sites. Next, we analyzed the relative and absolute information flow among the four multimodal subtypes. Relative information flow represents the relative importance of a specific communication pathway across all subtypes (**Figure 7C**). Across multiple communication pathways, the CS1 subtype exhibited higher relative information flow, such as in the TNF, IL16, and Notch signaling pathways, indicating that these pathways have greater relative importance in the CS1 subtype. The CS2 subtype exhibited lower relative information flow across multiple communication pathways, which is consistent with the previously observed reduction in the number and strength of cell-cell communication in this subtype. However, the HGF (Hepatocyte Growth Factor) signaling pathway was found to be aberrantly activated in the CS2 subtype. HGF primarily binds to its receptor c-Met, and this pathway is known to play a crucial role in regulating various cellular behaviors, such as proliferation, migration, invasion, and apoptosis (131, 132). The CS3 subtype has relatively higher information flow in some specific communication pathways, such as SELPLG and CEACAM, but overall exhibits relatively lower information flow. The CS4 subtype has relatively higher information flow across multiple pathways, such as ICAM, LIFR, SN, EPHB, TGFb, COMPLEMENT, and CD45, indicating that these pathways are more important in the CS4 subtype. Absolute information flow represents the actual information flow quantity of a specific communication pathway in each subtype. Pathways such as MK, CypA, MHC-II, and LAMININ exhibit higher absolute information flow in the CS4 subtype, indicating that these pathways are more active in these subtypes. Furthermore, we analyzed the overall differences in signal pattern across the four multi-modal subtypes (**Figure 7D**; **Supplementary Figure S11**).In the CS1 subtype, we found that Fibroblasts and NK cells are the primary information sources, with Fibroblasts being the dominant information senders (communication signals such as COLLAGEN, FN1, MK, etc.), while NK cells are the primary information receivers (signals such as MHC-I, CXCL, GALECTIN, LCK, etc.). The elevated signal pathway activity of MIF in B cells and CD8 T cells is a key feature of the CS1 subtype. This suggests that in the tumor microenvironment of the CS1 subtype, Fibroblasts and NK cells may play critical roles in regulating tumor cell behavior and immune status. The aberrant activation of the MIF signaling pathway may be an important biological characteristic of the CS1 subtype, potentially impacting tumor progression and immune status. In the CS2 subtype, the overall communication signal activity is relatively low. The LAIR1 (Leukocyte-Associated Immunoglobulin-Like Receptor 1) communication signal is responsible for signal transduction between Mono/Macro (Monocyte and Macrophage) cells. While overall cell-cell communication in CS2 was markedly reduced (as shown in **Figures 7A, B**), the interactions that did occur were disproportionately enriched for immune inhibitory pathways, notably LAIR1 signaling between monocytes/macrophages. This suggests a microenvironment where, despite limited cellular crosstalk, immunosuppression predominates. LAIR1, an inhibitory receptor expressed on immune cells, binds to collagen and can suppress immune cell activation (133). The observed LAIR1 signaling between Mono/Macro cells in the CS2 subtype suggests significant immune regulatory and inhibitory signaling within this cell population. This signal transmission may lead to reduced immune cell activity, thereby impacting the immune response in the tumor microenvironment and allowing tumor cells to evade immune surveillance. The JAM (Junctional Adhesion Molecule) communication signal acts as a sender in Fibroblasts and a primary receiver in Malignant cells. JAM family members participate in tight cell-cell junctions and signal transduction, typically expressed in epithelial and endothelial cells (134). The JAM signaling pathway plays a crucial role in cell adhesion, migration, and immune cell traversal (135). This communication could promote the adaptation and progression of malignant cells within the tumor microenvironment, particularly in the interaction between tumor cells and stromal cells. Furthermore, the Hepatocyte Growth Factor (HGF) communication signal acts as a sender in Fibroblasts and a primary receiver in Epithelial cells in the CS2 subtype. This suggests that Fibroblasts may exert a significant impact on Epithelial cells through the secretion of HGF, potentially promoting epithelial cell proliferation and migration. The aberrant activation of the HGF signaling pathway may drive the malignant transformation of Epithelial cells and tumor progression in the CS2 subtype, enhancing their invasive and metastatic capabilities (136). In the CS3 subtype, the SELPLG (Selectin P Ligand), also known as PSGL-1 (P-selectin glycoprotein ligand-1), communication signal primarily originates from NK (natural killer) cells, while the endothelial cells act as the signal receivers. SELPLG is an adhesion molecule expressed on the surface of leukocytes, which can bind to selectins (such as P-selectin, E-selectin, and L-selectin), playing a crucial role in the processes of inflammation, immune cell rolling, adhesion, and migration (137). Through the SELPLG signaling, NK cells interact with endothelial cells, which may enhance the adhesion and migratory capabilities of endothelial cells in the context of inflammation and immune responses. This interaction between NK cells and endothelial cells may facilitate the localization and infiltration of NK cells within the tumor microenvironment, while also potentially modulating the tumor vascular system, thereby enabling more effective immune surveillance and tumor cell killing. In the CS4 subtype, the cell types with the strongest communication signals are Fibroblasts and Mono/Macro cells. We have found that the CD45, COMPLEMENT, and TGFb communication signals predominantly occur within the Mono/Macro cell population. The CD45 signal is sent by NK cells and received by Mono/Macro cells. The COMPLEMENT signal is sent by Fibroblasts and received by Mono/Macro cells. The SEMA4 communication signal is sent by NK cells and received by Malignant and Mono/Macro cells. The Prostaglandin communication signal is primarily observed within Mono/Macro cells, with the senders being CD8 T cells and Malignant cells, and the receivers being Mono/Macro cells. These findings reflect the important role of Mono/Macro cells in regulating the inflammatory response and tumor progression within the tumor microenvironment of the CS4 subtype. The intricate communication networks involving multiple cell types, including Fibroblasts, NK cells, CD8 T cells, and Malignant cells, converge on the Mono/Macro cell population, highlighting their central role in shaping the tumor ecosystem in this particular subtype.

### 3.5 Comparison of drug response-associated cells in multimodal subtypes of breast cancer neoadjuvant therapy

**Figure 8:**
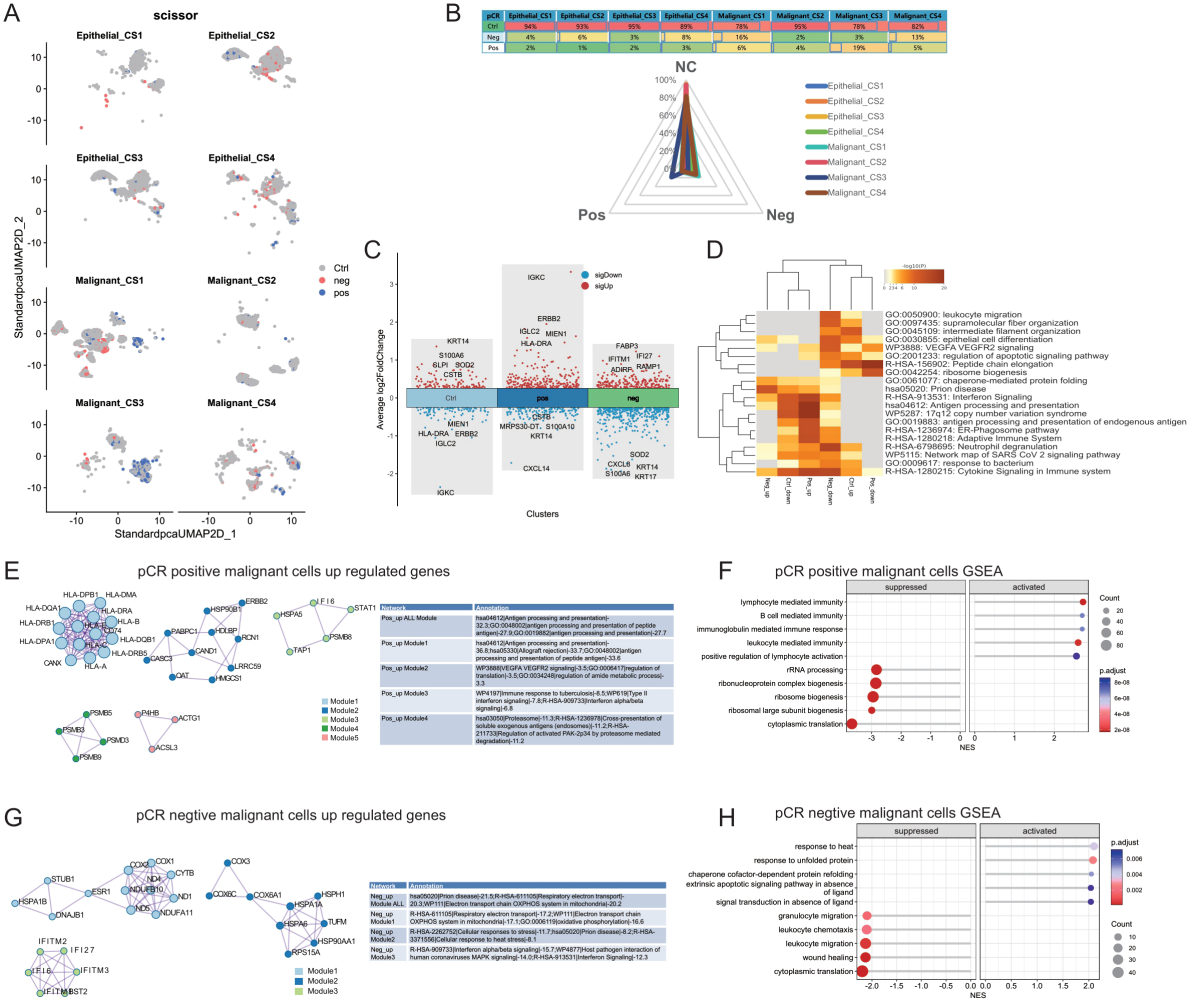
Comparison of Drug Response-Related Cells in Four Breast Cancer Multimodal Subtypes. **(A)** Distribution of positive (pCR positive) and negative (pCR negative) response cells across eight cell subtypes (Epithelial_CS1, Epithelial_CS2, Epithelial_CS3, Epithelial_CS4, Malignant_CS1, Malignant_CS2, Malignant_CS3, Malignant_CS4) in a dimensionality reduction scatter plot. **(B)** Distribution of control (Ctrl), pCR positive (Pos), and pCR negative (Neg) cells within the eight cell subtypes. **(C)** Comparison of differentially expressed genes between pCR positive cells, pCR negative cells, and control cells, focusing exclusively on malignant cells. **(D)** Heatmap of functional enrichment analysis for differentially expressed genes. **(E)** Protein-protein interaction (PPI) network and functional enrichment analysis of upregulated genes in pCR positive malignant cells. **(F)** Gene set enrichment analysis (GSEA) results for pCR positive malignant cells. “Activated” denotes pathways enriched with genes highly expressed in pCR positive malignant cells, while “Suppressed” indicates pathways enriched with genes lowly expressed in these cells. **(G)** PPI network and functional enrichment analysis of upregulated genes in pCR negative malignant cells. **(H)** GSEA results for pCR negative malignant cells. “Activated” denotes pathways enriched with genes highly expressed in pCR negative malignant cells, while “Suppressed” indicates pathways enriched with genes lowly expressed in these cells.

To further explore the relationship between disease and drug response, we utilized gene expression data from the ISPY2 cohort and subsets of epithelial and malignant cells from the GSE161529 cohort. Using the Scissor computational framework, we investigated cells associated with pathological complete response (pCR) outcomes. We conducted assessments within each of the four multimodal subtypes. We found that in the CS1 subtype, 10% of cells were non-Response, and 4% were Response; in the CS2 subtype, 4% were non-Response, and 3% were Response; in the CS3 subtype, 3% were non-Response, and 10% were Response; and in the CS4 subtype, 11% were non-Response, and 4% were Response(**Figures 8A, B**). Furthermore, we performed analyses separately for epithelial and malignant cells across the four different multimodal subtypes. We observed that in the CS1 subtype, 4% of epithelial cells were non-Response, and 2% were Response, while 16% of malignant cells were non-Response, and 6% were Response; in the CS2 subtype, 6% of epithelial cells were non-Response, and 1% were Response, while 2% of malignant cells were non-Response, and 4% were Response; in the CS3 subtype, 3% of epithelial cells were non-Response, and 2% were Response, while 3% of malignant cells were non-Response, and 19% were Response; in the CS4 subtype, 8% of epithelial cells were non-Response, and 3% were Response, while 13% of malignant cells were non-Response, and 5% were Response. We found a higher proportion of non-Response in epithelial cells, whereas in malignant cells, non-Response rates in CS1 and CS4 subtypes were higher compared to all epithelial and malignant cell subgroups, at 16% and 13% respectively. Conversely, the Response rate was highest in CS3 subtype malignant cells (19%). This result aligns with the earlier analysis, indicating lower pCR rates in CS1 subtype and higher pCR rates in CS3 subtype. However, the lower response rate in the CS4 subtype is somewhat inconsistent, possibly due to its intense immune activation status. From the preceding conclusions, we observed that malignant cells exhibit a more pronounced effect on pCR response compared to epithelial cells. Next, we performed differential expression analysis on Pos (Response), Neg (non-Response), and Control (not effective) groups within malignant cells. We employed the Wilcoxon test for differential analysis using a one-vs-others strategy.Through differential expression analysis (|LogFC| > 0.5, P.adj > 0.05), we identified notable gene expression changes across various groups. In the Control group, 21 genes were found to be overexpressed, including KRT14, S100A6, SLPI, and SOD2, while 14 genes were underexpressed, such as IGKC, IGLC2, ERBB2, and HLA-DRA. The Pos group exhibited upregulation of 63 genes, including IGKC, ERBB2, IGLC2, MIEN1, and HLA-DRA, and downregulation of 15 genes, including CXCL14, S100A10, CSTB, and PDSS2. In the Neg group, 44 genes were upregulated, including FABP3, IFI27, IFITM1, and ADIRF, whereas 98 genes were downregulated, including KRT17, KRT14, S100A6, CXCL8, and SOD2(**Figure 8C**). Based on the differentially expressed genes, we conducted functional enrichment analysis using the multi-genelist mode of Metascape, constructed a PPI network, and identified core modules using the MCODE algorithm (**Figure 8D** **;** **Supplementary Figures S12A, B**). We found that genes upregulated in the Pos group were mainly enriched in Antigen processing and presentation, 17q12 copy number variation syndrome, while downregulated genes were mainly enriched in Peptide chain elongation, ribosome biogenesis pathways. Genes upregulated in the Neg group were predominantly enriched in Prion disease, Interferon Signaling, while downregulated genes were mainly enriched in leukocyte migration, supramolecular fiber organization, epithelial cell differentiation pathways. Control group upregulated genes were also enriched in Peptide chain elongation, intermediate filament organization pathways, while downregulated genes exhibited enrichment similar to the pathways enriched by upregulated genes in the Pos group. Furthermore, we explored the PPI networks of upregulated and downregulated genes in the Pos group, and upregulated and downregulated genes in the Neg group separately (**Figure 8E, G**; **Supplementary Figures S12C–F**). We found five core regulatory modules in the PPI network of upregulated genes in the Pos group. Module1 primarily participated in Antigen processing and presentation; Module2 regulated VEGFA VEGFR2 signaling; Module3 was enriched in immune response to tuberculosis; Module4 regulated Proteasome. There was only one core module identified in the PPI network of downregulated genes in the Pos group, mainly involved in Peptide chain elongation, Viral mRNA Translation. The PPI module of upregulated genes in the Neg group mainly regulated the Electron transport chain OXPHOS system in mitochondria and Oxidative phosphorylation pathways. It comprised three core modules: Module1 mainly participated in Respiratory electron transport, oxidative phosphorylation; Module2 was involved in Cellular response to stress, Prion disease; Module3 was associated with Interferon alpha/beta signaling, Interferon Signaling pathways.

Furthermore, we utilized the GSEA algorithm to assess the enriched signaling pathways of the differentially expressed genes in both the Pos and Neg groups (**Figures 8F, H**). Utilizing ClusterProfiler’s gseKEGG, we conducted functional enrichment analysis on the gene lists of differentially expressed genes from the Pos and Neg groups (sorted by logFC). In the Pos group, we observed significant activation of pathways such as lymphocyte mediated immunity, B cell mediated immunity, immunoglobulin mediated immune response, leukocyte mediated immunity, while pathways including cytoplasmic translation, ribosomal large subunit biogenesis, ribosome biogenesis were significantly activated. In the Neg group, we found activation of pathways including response to heat, response to unfolded protein, chaperone cofactor-dependent protein refolding, while pathways such as cytoplasmic translation, wound healing, leukocyte migration were significantly suppressed. These results are consistent with the findings from the earlier enrichment analysis.

## 4 Discussion

Neoadjuvant therapy, encompassing chemotherapy, radiotherapy, endocrine therapy, and targeted agents, is increasingly utilized in breast cancer management prior to surgery (138). In recent years, the application of neoadjuvant therapy in breast cancer has become increasingly widespread, playing a significant role in improving the survival rates and quality of life for breast cancer patients (139). Despite the growing application of neoadjuvant therapy in breast cancer, there are relatively few multimodal prediction models specifically tailored for neoadjuvant therapy (140). These prediction models are mainly based on clinical and pathological features, molecular biomarkers, and imaging examinations, aiming to predict the efficacy and prognosis of neoadjuvant therapy (141, 142). Pathological complete response (pCR) is considered one of the indicators of successful neoadjuvant therapy and is closely related to long-term survival rates in patients (143). However, there are relatively few studies on molecular subtyping based on pCR after neoadjuvant therapy. In this project, we constructed a multimodal model to predict breast cancer pCR based on the ISPY2 cohort, which includes transcriptomics, MRI, and proteomics data. Feature extraction was performed using lasso regression, followed by the construction of predictive models for breast cancer neoadjuvant therapy pCR based on five machine learning models. The results showed that our ridge regression model, integrating transcriptomics, imagingomics, and proteomics, achieved the best predictive performance on the validation set (AUC=0.917, CA=0.827, F1 = 0.818, Prec=0.822, Recall=0.823, MCC=0.611). By integrating features from multiple modalities, we obtained the weights of these features(46 features from proteomics, 60 features from transcriptomics, and 42 features from radiomics). We found that features such as original_shape_Maximum2DDiameterColumn, HLA.DPB2, Cyclin.D1.total, PSMD3, ERBB2.total, MYCN had higher weights. Original_shape_Maximum2DDiameterColumn is an imaging feature used to describe the maximum diameter of tumors in the coronal plane, which is an important parameter for describing tumor size and shape. This indicator has been used in various cancer studies, including but not limited to lung cancer, brain cancer, and liver cancer. Cyclin D1 is a cell cycle protein that binds to cyclin-dependent kinases (CDKs), particularly CDK4 and CDK6, promoting cell entry from G1 phase to S phase, thereby driving cell division and proliferation (144). Overexpression of Cyclin D1 is associated with the development of various cancers, including breast cancer, prostate cancer, and pancreatic cancer (145, 146). Therefore, the level of Cyclin D1 can serve as a biomarker for cancer diagnosis and prognosis. ERBB2.total (Erb-B2 Receptor Tyrosine Kinase 2), also known as HER2 in certain contexts, is a receptor tyrosine kinase involved in signaling pathways regulating cell growth and differentiation (147). ERBB2 is overexpressed in certain cancers, particularly in breast cancer, and is associated with disease invasiveness and poor prognosis (148). MYCN is a member of the MYC gene family, acting as a transcription factor involved in regulating cell proliferation, differentiation, and apoptosis (149). Aberrant expression of MYCN is associated with the development of various cancers, including neuroblastoma, small cell lung cancer, and other types of tumors (150). Thus, the features selected by our machine learning models for predicting breast cancer neoadjuvant therapy pCR have significant clinical and biological implications. These features can aid researchers in the development of breast cancer-related drug therapies.

Next, we performed unsupervised clustering analysis on multimodal data related to pCR obtained from transcriptomics, proteomics, and MRI. Through nine unsupervised clustering algorithms, we identified four multimodal subtypes relevant to neoadjuvant therapy in breast cancer. We found significant differences in transcriptomic, proteomic, and MRI features among these four multimodal subtypes. Comparing clinical features, we observed that the pCR rate was lowest in CS1, at only 11%, while CS3 exhibited the highest rate at 52%. CS1 subtype consisted entirely of ER+ patients, whereas CS2 and CS4 comprised 77% and 80% ER- patients, respectively. Additionally, we found that over 90% of patients in CS2 and CS4 were HER2-. From the perspective of PAM50 subtyping, 96% of CS2 and 92% of CS4 belonged to the Basal subtype, while 74% of CS1 belonged to the Luminal B subtype. These results were consistent with previous pathological findings.

Through GSVA analysis, we compared HALLMARK scores among the four subtypes and found that estrogen response-related signaling pathways were significantly activated in the CS1 subtype, consistent with the results of protein expression. Considering that CS1 mainly comprised ER+ patients, this result suggests that the efficacy of neoadjuvant therapy in ER+ patients may be suboptimal. KEGG pathway enrichment analysis using GSEA revealed that lipid metabolism and oxidative phosphorylation pathways were more active in CS1, DNA repair-related pathways were more active in CS2, energy metabolism activities were more active in CS3, and inflammation-related signaling pathways were more active in CS4.

Next, we explored the response of these four multimodal subtypes to immunotherapy. We compared Response and Non-Response groups in each subtype using 11 published immunotherapy response scores. We found that the pCR response group exhibited better immunotherapy outcomes, which is closely related to the mechanism of immunotherapy. Immunotherapy works by activating or enhancing the patient’s immune system to attack tumor cells. Therefore, the degree of immune cell infiltration in the tumor microenvironment is one of the key factors affecting treatment outcomes. Among the four multimodal subtypes, CS1 showed the worst immunotherapy response, while CS4 demonstrated the best response. We obtained similar results in the TCGA-BRCA cohort. Due to the high immune cell infiltration in CS4, it is more likely to respond to immunotherapy.

Furthermore, we evaluated the differences in drug response among the four multimodal subtypes. We predicted the sensitivity of commonly used breast cancer chemotherapy drugs in the ISPY2, TCGA-BRCA and Metabrick-BRCA cohorts using transcriptomics. The results indicated that CS1 exhibited resistance to Cisplatin, Gemcitabine, Paclitaxel, and Vinorelbine, while CS2 and CS4 showed better sensitivity to chemotherapy drugs. We conducted a comparative analysis of multimodal information among the four multimodal subtypes in the TCGA-BRCA cohort. Firstly, we found that the prognosis of CS1 and CS4 subtypes was the most favorable (Overall survival, Disease-specific survival, Progression survival) in the TCGA-BRCA cohort. By comparing three hypoxia scores, we observed that the hypoxia scores of CS1 and CS4 were relatively low, while CS2 had the highest hypoxia score. Hypoxia scores typically reflect insufficient oxygen supply in the tumor microenvironment, which is related to tumor growth, invasiveness, and treatment response. Lower hypoxia scores suggest that tumors in CS1 and CS4 subtypes may have better oxygen supply. Considering the earlier analysis, the high oxidative phosphorylation activity in the CS1 subtype and the lowest Hypoxia score inferred by the Progeny algorithm among all groups suggest a better prognosis for the CS1 subtype, possibly due to adequate oxygen supply. Conversely, the CS4 subtype is associated with high immune cell infiltration. Numerous studies have shown that immune cell infiltration in tumor cells contributes to the prognosis of solid tumors such as lung cancer, pancreatic cancer, and breast cancer (151). Surprisingly, we also found that male breast cancer patients mainly belonged to the CS1 subtype, while the CS4 subtype was predominant in the American Indian or Alaska Native population. This finding warrants further exploration and analysis.

Through comparative analysis of TCGA-BRCA exome sequencing data, we found that among the four multimodal subtypes, the CS2 subtype had the highest mutation rate, while the CS1 subtype had the lowest mutation rate. Mutation rates are usually associated with tumor differentiation and malignancy. Highly differentiated tumors typically have lower mutation rates, whereas poorly differentiated or undifferentiated tumors tend to have higher mutation rates. In this study, the low mutation rate in the CS1 subtype may indicate a higher degree of tumor cell differentiation, which may be associated with its better prognosis. Conversely, the high mutation rate in the CS2 subtype may reflect its lower degree of differentiation and higher malignant potential. We also observed that the mutation rate of MAP3K1 in the CS1 subtype was significantly higher than in the other three subtypes, while TP53 mutations were significantly lower than in the other three subtypes. The significantly higher mutation rate of MAP3K1 in the CS1 subtype than in other subtypes may have specific effects on the biological behavior of tumors. MAP3K1 is a component of the mitogen-activated protein kinase (MAPK) pathway, which plays a crucial role in cell proliferation and differentiation (152). Mutations in MAP3K1 may affect the activity of the MAPK pathway, thereby influencing the behavior of tumor cells (152). TP53 is a tumor suppressor gene, and its mutations are commonly found in various cancers, closely associated with tumor development and progression (153). Loss-of-function mutations in TP53 typically lead to cell cycle dysregulation and defects in DNA repair mechanisms, thereby increasing the malignancy of tumors (154). The lower mutation rate of TP53 in the CS1 subtype may indicate that tumor cells in this subtype retain certain tumor suppressor functions, which may be associated with its lower malignancy and better prognosis.

Based on the previous analysis, we identified that the differences in tumor microenvironments among the four multimodal subtypes are the main reasons for the differential efficacy of neoadjuvant therapy. To further elucidate the tissue microenvironment differences among the four multimodal subtypes, we utilized single-cell sequencing data to analyze the tumor microenvironments of each subtype individually. Our study indicated that the CS1 subtype has the highest proportion of malignant cells (77.2%), while the CS4 subtype has the highest proportion of immune cells (B 5.7%, CD4Tconv 16.6%, Mono/Macro 12.6%, NK 8.8%, Plasma 5.1%). The CS2 subtype exhibits the highest proportion of epithelial cells (46.5%). These findings are consistent with the results obtained through bulk RNA sequencing analysis.

A key finding of our study is the profound heterogeneity in the cellular origins and evolutionary trajectories of malignant cells across the four identified multimodal breast cancer subtypes. By integrating single-cell RNA-sequencing data with our multi-omics classification, we were able to dissect the complex interplay between genomic instability, differentiation status, and the tumor microenvironment (TME) in shaping these trajectories. The CS1 subtype, characterized by ER-positivity, low genomic instability, and a relative lack of immune infiltration, presented a comparatively “quiescent” picture at the single-cell level. Both Epithelial and Malignant cells exhibited low CNV burdens and low CytoTRACE differentiation scores, suggesting a closer resemblance between these cell populations and a potentially less aggressive phenotype. This is consistent with the known biology of Luminal B breast cancers, which often exhibit lower mutation rates and a better prognosis than other subtypes (16). The relative insensitivity of CS1 to neoadjuvant chemotherapy, observed both in our predictive model and in the drug response analysis, may be linked to this lower degree of genomic alteration and the predominance of cells in a less proliferative state. In stark contrast, the CS2 subtype, predominantly ER-negative and Basal-like, displayed a highly unstable genomic landscape, with malignant cells exhibiting the highest CNV burden. The elevated CytoTRACE differentiation scores in both ‘Epithelial’ and ‘Malignant’ cells, coupled with the prevalence of immunosuppressive signals in the TME, suggest a trajectory driven by rapid genomic diversification and active immune evasion. This aligns with the known aggressive behavior and poorer prognosis of many Basal-like breast cancers (155). The enrichment of pathways related to angiogenesis and endothelial cell chemotaxis in the differentiation trajectories further supports the notion of a highly invasive phenotype. The CS3 subtype presented a distinct evolutionary profile. While Monocle2 analysis revealed differentiation trajectories enriched for tissue homeostasis pathways, the high mutation burden and intermediate immune infiltration suggest a complex interplay of factors. This subtype may represent a distinct evolutionary path where, despite attempts at maintaining tissue architecture, genomic instability ultimately drives aggressive behavior. Further investigation is needed to fully elucidate the specific mechanisms driving tumorigenesis in CS3. Perhaps the most intriguing findings relate to the CS4 subtype. Despite exhibiting a high CNV burden in malignant cells, similar to CS2, CS4 is characterized by a robust immune infiltrate and, crucially, the best prognosis among the four subtypes. The high CytoTRACE differentiation scores in CS4 malignant cells, seemingly paradoxical given the intense immune pressure, likely reflect a process of immune selection. We hypothesize that the strong immune presence in the CS4 TME eliminates the most highly malignant and immunogenic cells, leading to a relative enrichment of cells that have either adapted to evade immune detection or exhibit lower genomic instability, and lower inherent aggressiveness. This “immune editing” process (156) may contribute significantly to the favorable clinical outcomes observed in this subtype. This also aligns with previous reports regarding tumor infiltrating lymphocytes in breast cancer (157).

The distinct differentiation trajectories identified by Monocle2, with their subtype-specific pathway enrichments, provide valuable insights into the potential mechanisms driving malignant progression in each subtype. While all subtypes show some modulation of shared pathways like cell cycle regulation, the unique combinations of activated pathways and TME interactions likely dictate the specific therapeutic vulnerabilities of each subtype. For example, the prominence of angiogenesis-related pathways in CS2 suggests a potential sensitivity to anti-angiogenic therapies, while the immune evasion mechanisms in CS4 highlight the potential for immune checkpoint blockade. It is important to acknowledge that our interpretations of ‘differentiation’ are based on transcriptomic data and do not directly reflect histological grading. Further studies, ideally incorporating spatial transcriptomics and matched histological analyses, are needed to fully validate these findings and to correlate the inferred differentiation states with established pathological features. However, our integrated multi-omics and single-cell approach provides a powerful framework for dissecting the complex cellular dynamics within breast tumors and for identifying potential targets for more precise and effective therapeutic interventions.

Finally, we utilized the Scissor computational framework to jointly analyze single-cell sequencing data and bulk RNA-seq data to investigate the drug response of neoadjuvant therapy. We found that the CS1 and CS4 subtypes had the highest proportion of non-responsive malignant cells, while the CS3 subtype had the highest proportion of responsive cells to neoadjuvant therapy. Considering the lower degree of malignancy of cells in the CS1 and CS4 subtypes, this may be one of the main reasons. Conversely, the higher responsiveness of cells in the CS3 subtype may be due to the higher malignancy of its tumor cells. The CS2 subtype exhibited the fewest responsive cells to neoadjuvant therapy, possibly due to its higher genomic instability.

Furthermore, we compared the differences between the pCR response group (Pos group) and the non-response group (Neg group) among malignant cells. We found that highly expressed genes in the Pos group were mainly enriched in inflammation-related signaling pathways, especially the 17q12 copy number variation syndrome. Inflammatory response is a key component of the tumor microenvironment and can influence tumor development and treatment response through various mechanisms. The 17q12 copy number variation syndrome refers to copy number variations (CNV) occurring in the chromosome 17q12 region, which may affect the expression of multiple genes in this region, including ERBB2 (HER2), a known therapeutic target for breast cancer (158). CNV can alter the expression levels of these genes, thereby affecting the biological behavior of tumors and their sensitivity to treatment (159). Highly expressed genes enriched in the Neg group were mainly involved in oxidative phosphorylation and respiratory electron transport pathways. This result is consistent with the signaling pathways enriched in the CS1 subtype identified in bulk RNA-seq analysis, indicating that energy metabolism generated by aerobic respiration negatively affects the efficacy of neoadjuvant therapy.

In the analysis of low-expressed genes in the non-response to neoadjuvant therapy (Neg group), protein-protein interaction (PPI) regulatory networks revealed that the core modules were mainly enriched in neutrophil degranulation and cytokine signaling in the immune system. Neutrophils are a type of white blood cell and play a crucial role in the body’s defense against infection. Degranulation is the release of granules containing antimicrobial proteins and enzymes by neutrophils, which play a key role in inflammation and immune defense (160). Tumor-associated neutrophils (TANs) have diverse roles in the tumor microenvironment, including anti-tumor (N1 phenotype) and pro-tumor (N2 phenotype) effects (161). Existing research suggests that chemotherapy resistance may be primarily due to the involvement of neutrophils in promoting a pro-tumor phenotype (162).

In summary, we have constructed a multi-modal predictive model for the efficacy of neoadjuvant therapy in breast cancer, analyzed multi-modal subtypes based on predictive features of neoadjuvant therapy, and delved into the cellular heterogeneity of response to neoadjuvant therapy using single-cell sequencing data. We identified four distinct multi-modal subtypes exhibiting significant differences in prognosis, response to neoadjuvant therapy, tumor immune microenvironment, cellular differentiation, malignancy, among other aspects. These findings not only deepen our understanding of the pathogenesis of breast cancer but also provide novel insights and methodologies for precision treatment of breast cancer.

Our study, while offering valuable insights, is subject to limitations warranting careful consideration, including reliance on publicly available data and open-source computational models, necessitating experimental corroboration to address potential in silico biases and confirm findings. Crucially, the demographic composition of the datasets, particularly ISPY2’s strong bias towards White, Non-Hispanic/Latino patients (and similar likely limitations in TCGA-BRCA and METABRIC), significantly restricts generalizability regarding population-specific characteristics and ethnic disparities in breast cancer subtypes; furthermore, insufficient treatment data granularity (omitted dosages/schedules in ISPY2, incomplete data and “Not Available” responses in TCGA-BRCA, and lacking chemotherapy regimen specifics in METABRIC) precludes a comprehensive assessment of treatment efficacy across subtypes. Variability in laboratory methodologies across datasets (gene expression, protein detection) without standardized protocols introduces potential confounding factors, while the potential omission of emerging biomarkers limits full characterization of underlying biological mechanisms; finally, the exclusive focus on neoadjuvant therapy response in breast cancer necessitates further investigation to determine relevance to other malignancies and therapeutic strategies.

In the future, we plan to experimentally validate our findings and further investigate the specific mechanisms of action of these multi-modal subtypes in the occurrence and development of breast cancer. Additionally, we aim to apply these methods to research on other types of cancer and treatment modalities to provide more valuable information for precision treatment of cancer. Overall, our study provides a new perspective and approach for predicting the response to neoadjuvant therapy and analyzing subtypes in breast cancer, opening up new possibilities for precision treatment. We look forward to making greater contributions to the treatment and prevention of cancer through further research.

## Data Availability

The original contributions presented in the study are included in the article/**Supplementary Material**. Further inquiries can be directed to the corresponding authors.

## Supplementary material

Supplementary Figure 1 Predictive performance of models constructed using gene expression data, protein expression data, clinical data, and MRI radiomics features across four common machine learning algorithms (Lasso Regression, Random Forest, Gradient Boosting, and Support Vector Machines). The performance metrics include AUC, Calibration curve, Precision-Recall, Lift curve, and Cumulative Gains for both training and testing datasets. **(A, B)** Models constructed using gene expression data. **(C, D)** Models constructed using protein expression data. **(E, F)** Models constructed using clinical data. **(G, H)** Models constructed using MRI radiomics features.

Supplementary Figure 2 Performance of the model constructed using features selected via Lasso regression from three modalities (46 proteomics features, 60 transcriptomics features, and 42 radiomics features) combined with clinical features. The final model, obtained through another round of Lasso regression with 1000 permutations, includes 32 features. The performance metrics include AUC, Calibration curve, Precision-Recall, Lift curve, and Cumulative Gains. **(A)** Performance in the training set. **(B)** Performance in the validation set.

Supplementary Figure 3 Comparison of Four Breast Cancer Multimodal Subtypes. **(A, B)** Heatmap of protein expression profiles from the ISPY2 cohort across the four breast cancer multimodal subtypes, highlighting the top 20 most significantly different proteins. **(C)** Significantly different HALLMARKER scores. **(D)** Significantly different Progeny scores. **(E)** Significantly different xCell infiltration scores. **(F)** Significantly different CIBERSORT immune infiltration scores. **(G)** Significantly different transcription factor activity scores. **(H)** Significantly different MRI radiomic feature scores. Significance levels are indicated as follows: P<0.05 *, P<0.01 **, P<0.001 ***, P<0.0001 ****, NS (Not Significant).

Supplementary Figure 4 Comparison of immunotherapy and drug response across breast cancer multimodal subtypes. **(A)** Expression of marker genes in the ISPY2 cohort’s four multimodal subtypes, identified using the NTP algorithm. **(B)** Comparison of 10 immunotherapy scores between pCR-positive and pCR-negative groups in the ISPY2 cohort, indicating a potentially better immunotherapy response in the pCR-positive group. **(C)** Comparison of 12 TIDE-derived immunotherapy-related scores between pCR-positive and pCR-negative groups across the four multimodal subtypes in the ISPY2 cohort. **(D)** Comparison of 12 immunotherapy-related scores among the four multimodal subtypes in the TCGA BRCA cohort, showing similar results to the ISPY2 cohort, with the CS4 subtype exhibiting a better immunotherapy response. **(E)** Comparison of 12 immunotherapy scores between pCR-positive (1) and pCR-negative (0) groups in the TCGA BRCA cohort. **(F)** Comparison of drug response scores among breast cancer multimodal subtypes in the ISPY2 cohort. Drug scores were obtained from GDSC1 (up) and GDSC2 (down), visualizing the top 20 drugs with significant differences among the four subtypes. Results indicate that the CS1 subtype is generally resistant to most drugs, consistent with our findings.

Supplementary Figure 5 Potential drug predictions for the four breast cancer multimodal subtypes. Using the Connectivity Map (cmap) platform, genes highly expressed in each of the four subtypes were input to identify potential therapeutic drugs and their mechanisms of action (MOA) specific to each subtype. **(A)** Predicted drugs and MOA for the CS1 subtype. (B) Predicted drugs and MOA for the CS2 subtype. **(C)** Predicted drugs and MOA for the CS3 subtype. **(D)** Predicted drugs and MOA for the CS4 subtype. This analysis highlights the unique molecular profiles of each subtype and suggests potential targeted therapies tailored to these profiles, offering insights into personalized treatment strategies for breast cancer patients.

Supplementary Figure 6 Validation of molecular and drug response characteristics across breast cancer multimodal subtypes. **(A–F)** Validation of pathway activity and IC50 scores for four common chemotherapeutic drugs in the four subtypes within the TCGA BRCA cohort. **(G–L)** Validation of pathway activity and IC50 scores for the same chemotherapeutic drugs in the four subtypes within the Metabrick BRCA cohort. The analyses demonstrate consistent findings across both validation cohorts, underscoring the robustness of the molecular and drug response differences observed among the breast cancer multimodal subtypes. These results provide further evidence for the distinct biological and therapeutic profiles of each subtype, reinforcing the potential for subtype-specific treatment strategies in breast cancer.

Supplementary Figure 7 Comparative analysis of four breast cancer multimodal subtypes in the TCGA-BRCA and Metabric-BRCA cohorts. **(A)** Mutation frequency comparison of five significantly different genes (TP53, GATA3, PIK3CA, RICTOR, MAP3K1) within the TCGA-BRCA cohort. **(B)** Comparison of EBV virus scores across the four multimodal subtypes in the TCGA-BRCA cohort, showing statistically significant differences. **(C)** Comparison of HPV virus scores across the four multimodal subtypes in the TCGA-BRCA cohort, with no statistically significant differences observed. **(D–K)** Comparative analysis of PR Status, Pam50+Claudin-low subtype, Inferred Menopausal State, Chemotherapy response, 3-Gene classifier subtype, Hormone Therapy response, ER Status measured by IHC, and overall ER Status across the four multimodal subtypes within the Metabric-BRCA cohort. These comparisons collectively provide a comprehensive understanding of the distinct molecular and clinical characteristics associated with each breast cancer multimodal subtype, reinforcing the robustness and clinical relevance of the proposed subtype classification.

Supplementary Figure 8 Differential analysis of gene methylation, proteomics, and microbiomics across four breast cancer multimodal subtypes. The TCGA-BRCA cohort, possessing comprehensive multimodal data, was utilized to compare the four breast cancer multimodal subtypes across multiple molecular modalities. **(A)** Differential gene methylation analysis across the four breast cancer multimodal subtypes within the TCGA-BRCA cohort. The top 20 genes exhibiting significant methylation differences were selected, with the average beta value of all methylation sites for each gene used to represent its methylation level. **(B)** Functional enrichment analysis of differentially methylated genes. Highly methylated genes in the CS1 subtype were predominantly enriched in inflammation-related signaling pathways, suggesting suppression of these pathways in CS1. Conversely, the CS2 and CS4 subtypes showed high methylation in genes associated with inflammation-related pathways, indicating activation of these pathways. **(C, D)** Differential protein expression analysis and functional enrichment of differentially expressed proteins within the TCGA-BRCA cohort. **(E, F)** Negative correlation between gene expression and methylation levels of MMP7 and MUC1, respectively, illustrating the regulatory effect of methylation on the expression of these genes. **(G)** Significant negative correlation between gene expression and high methylation levels within the TCGA-BRCA cohort. **(H)** Differences in intratumoral microbiome composition across the four breast cancer multimodal subtypes within the TCGA-BRCA cohort (P<0.05 *, P<0.01 **, P<0.001 ***, P<0.0001 ****).

Supplementary Figure 9 Comparative analysis of the tumor microenvironment at the single-cell level across four breast cancer multimodal subtypes. **(A)** Volcano plots of differential expression analyses for Epithelial_CS1, Epithelial_CS2, Epithelial_CS3, Epithelial_CS4, Malignant_CS1, Malignant_CS2, Malignant_CS3, and Malignant_CS4. **(B, C)** Functional enrichment analysis results of highly expressed genes in the above eight cell subgroups, utilizing GO_BP and KEGG databases.

Supplementary Figure 10 Copy number variation, transcription factor analysis, and tumor evolution analysis in four breast cancer multimodal subtypes. **(A)** Copy number variation (CNV) analysis grouped by the four breast cancer multimodal subtypes, illustrating the distinct CNV profiles among the subtypes. **(B)** Transcription factor (TF) enrichment analysis for the eight cell subgroups (Epithelial_CS1, Epithelial_CS2, Epithelial_CS3, Epithelial_CS4, Malignant_CS1, Malignant_CS2, Malignant_CS3, and Malignant_CS4), highlighting key TFs driving the molecular characteristics of each subgroup. **(C–E)** Cell differentiation trajectory from epithelial cells to malignant cells in the CS1 subtype, depicting the stepwise evolution and key transitional states. **(F–H)** Cell differentiation trajectory from epithelial cells to malignant cells in the CS2 subtype, demonstrating the progression and cellular changes characteristic of this subtype. **(I–K)** Cell differentiation trajectory from epithelial cells to malignant cells in the CS3 subtype, showing the specific evolutionary pathway and intermediate stages. **(L–N)** Cell differentiation trajectory from epithelial cells to malignant cells in the CS4 subtype, outlining the transformation process and unique features of this subtype. These analyses provide comprehensive insights into the genetic alterations, transcriptional regulation, and evolutionary dynamics of breast cancer multimodal subtypes. The CNV analysis reveals subtype-specific genomic changes, while the transcription factor enrichment highlights the regulatory networks underlying each cell subgroup. The differentiation trajectories map the complex pathways through which epithelial cells evolve into malignant cells, offering a detailed view of tumor progression and potential targets for therapeutic intervention.

Supplementary Figure 11 Incoming and outgoing signaling patterns in four breast cancer multimodal subtypes. **(A)** Incoming signaling patterns of the four breast cancer multimodal subtypes, detailing the signals received by each of the 11 cell types within the tumor microenvironment. **(B)** Outgoing signaling patterns of the four breast cancer multimodal subtypes, illustrating the signals sent by each of the 11 cell types within the tumor microenvironment. These figures provide a detailed analysis of the directional flow of cellular communications in the tumor microenvironment of different breast cancer multimodal subtypes. The incoming signaling patterns **(A)** highlight which cell types are primarily receiving signals and how these patterns vary across subtypes. The outgoing signaling patterns **(B)** reveal the key signaling cell types and the nature of the signals they emit in each subtype. This comprehensive view of intercellular communication dynamics can help elucidate the complex interactions that drive tumor behavior and may identify critical nodes for therapeutic intervention in breast cancer.

Supplementary Figure 12 Functional Enrichment Analysis of Differentially Expressed Genes in Malignant Cells using Metascape. **(A, B)** Comparative distribution of six differential gene sets: Pos up, Pos down, Neg up, Neg down, Ctrl up, and Ctrl down. **(C)** Functional enrichment analysis of the six differential gene sets mentioned above, highlighting key biological pathways and processes. **(D)** Construction of a functional enrichment network based on the differential gene sets, illustrating the interconnected pathways and their roles in cellular functions. **(E)** Protein-protein interaction (PPI) network constructed from the differential gene sets, showcasing the interactions between proteins encoded by the differentially expressed genes. **(F)** Core hub gene regulatory network identified using the MCODE algorithm on the PPI network of differentially expressed genes. These figures provide a detailed overview of the functional enrichment analysis performed on differentially expressed genes in malignant cells, specifically comparing pCR positive cells, pCR negative cells, and control cells. The comparative distribution of differential gene sets **(A, B)** reveals distinct patterns of gene expression associated with different drug response statuses. The functional enrichment analysis **(C)** highlights the key biological processes and pathways that are differentially regulated in these gene sets, offering insights into the underlying mechanisms of drug response. The functional enrichment network **(D)** and PPI network **(E)** provide a comprehensive view of the interconnected pathways and protein interactions, respectively, that are involved in the drug response. The identification of core hub gene regulatory networks using the MCODE algorithm **(F)** further pinpoints critical genes and interactions that may play pivotal roles in determining the drug response in malignant cells. These analyses are crucial for understanding the molecular basis of drug response variability in breast cancer and could potentially guide the development of targeted therapeutic strategies.
