# Supplementary figures and images for: Multimodal diagnostic models and subtype analysis for neoadjuvant therapy in breast cancer
Source: Front Immunol. 2025 Mar 18;16:1559200. doi: 10.3389/fimmu.2025.1559200 (PMC11958217; doi:10.3389/fimmu.2025.1559200)

Gene Expression Data MachineLearning Building

Training Data 5x fold cross-validation

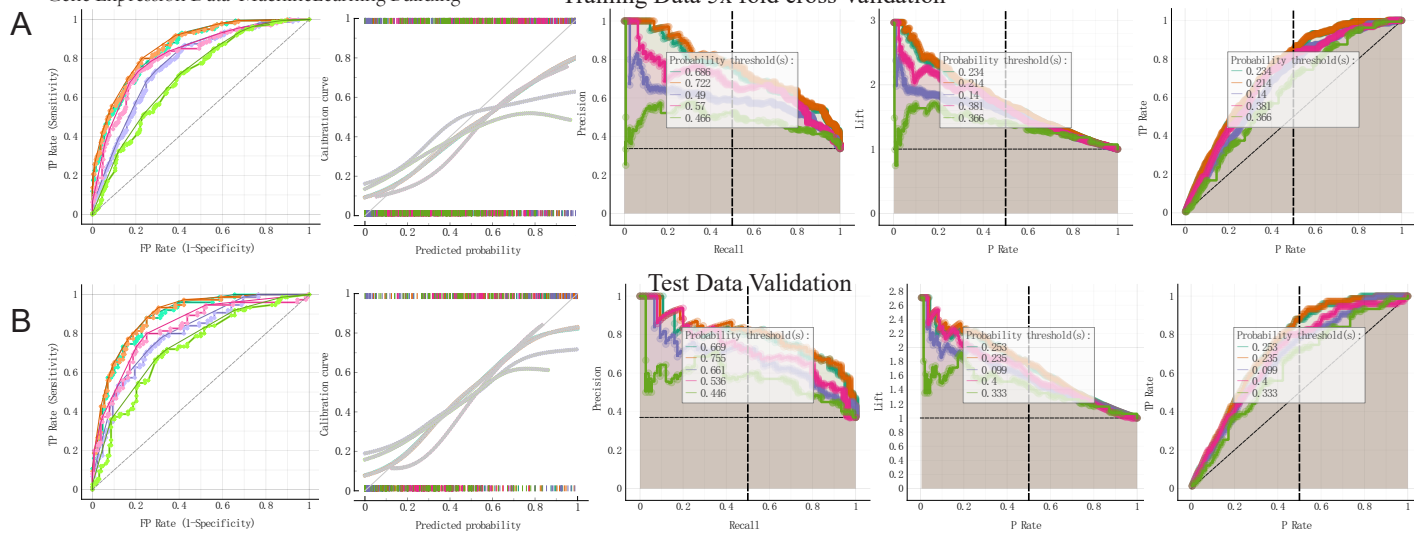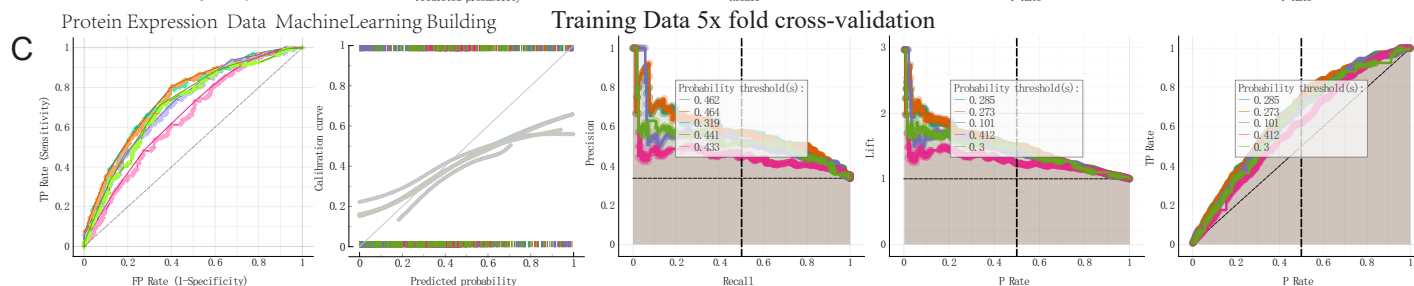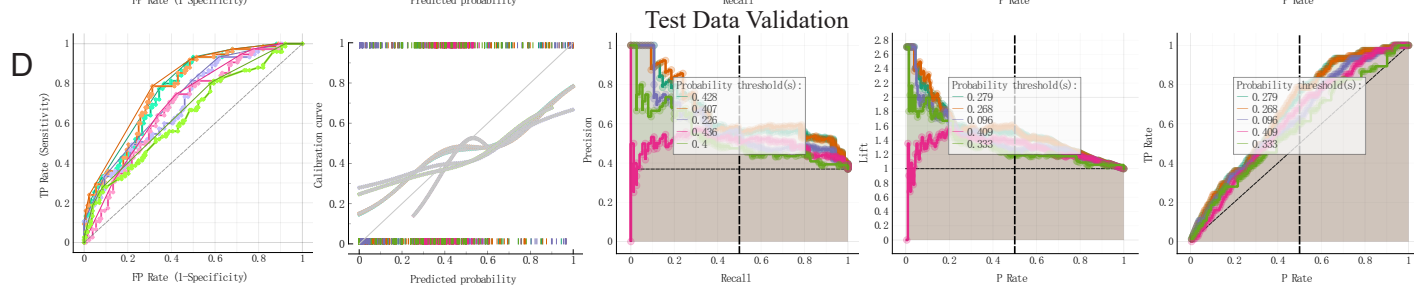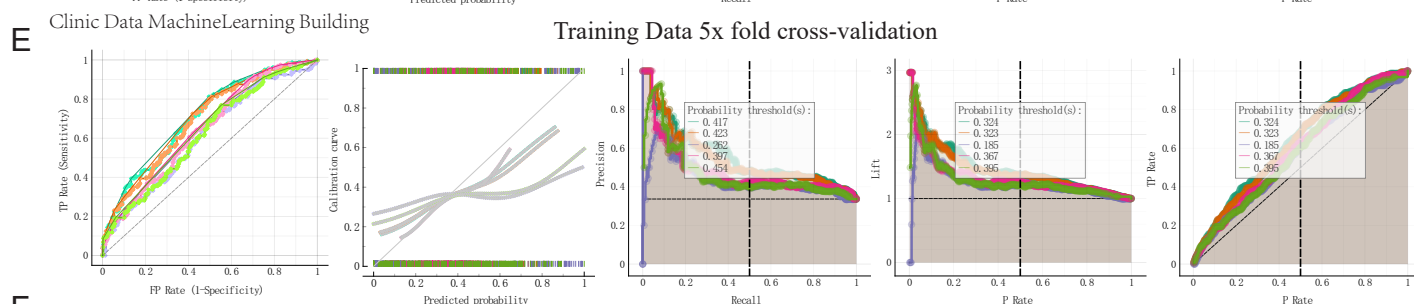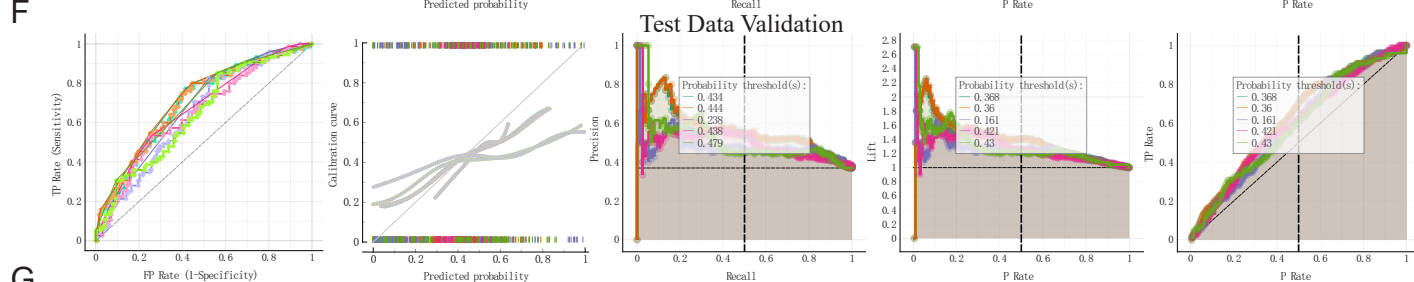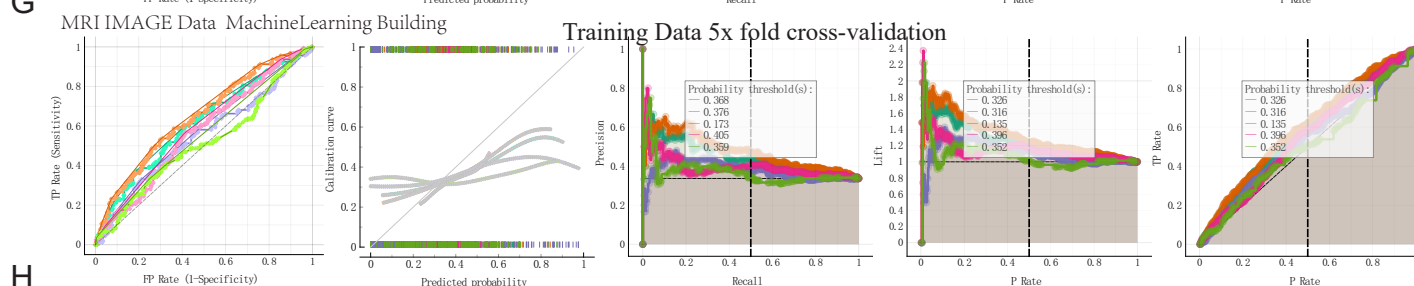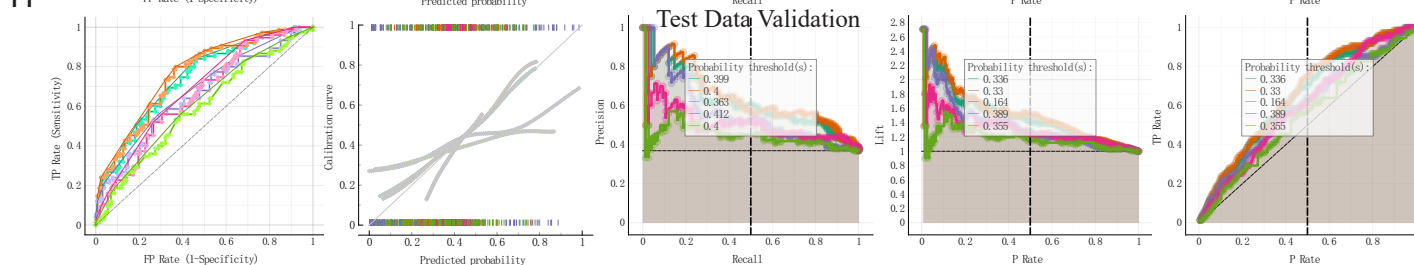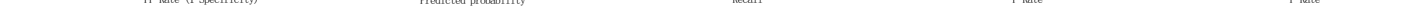

Supplement: Supplementary Figure 1 — Predictive performance of models constructed using gene expression data, protein expression data, clinical data, and MRI radiomics features across four common machine learning algorithms (Lasso Regression, Random Forest, Gradient Boosting, and Support Vector Machines). The performance metrics include AUC, Calibration curve, Precision-Recall, Lift curve, and Cumulative Gains for both training and testing datasets. (A, B) Models constructed using gene expression data. (C, D) Models constructed using protein expression data. (E, F) Models constructed using clinical data. (G, H) Models constructed using MRI radiomics features. [file DataSheet1.pdf]

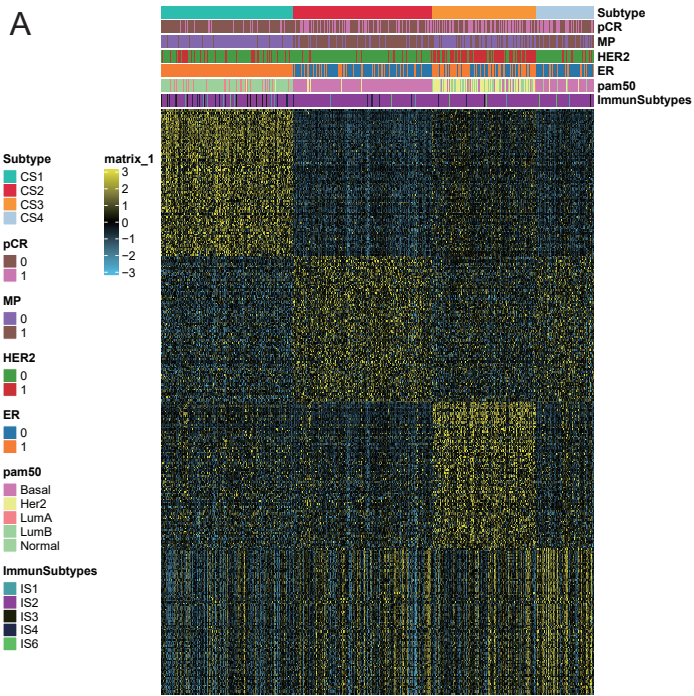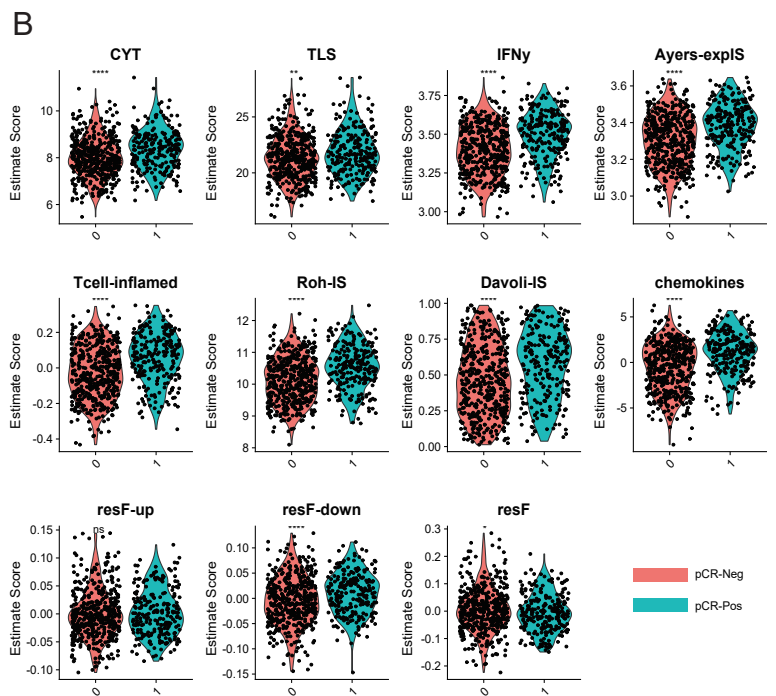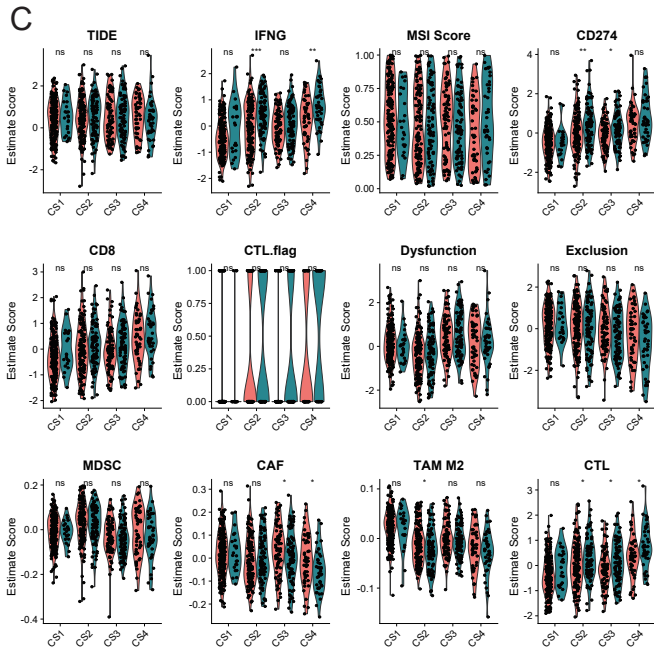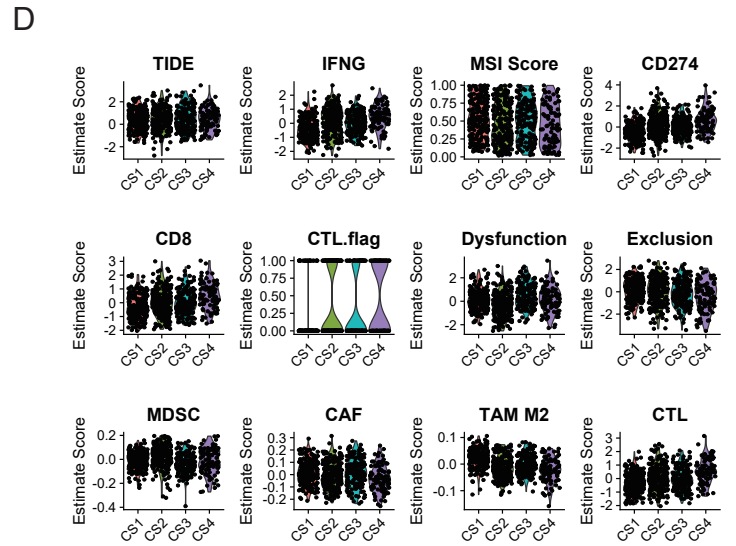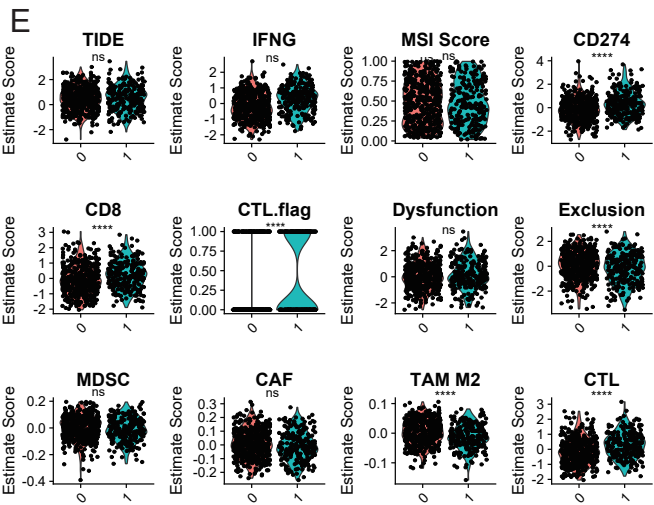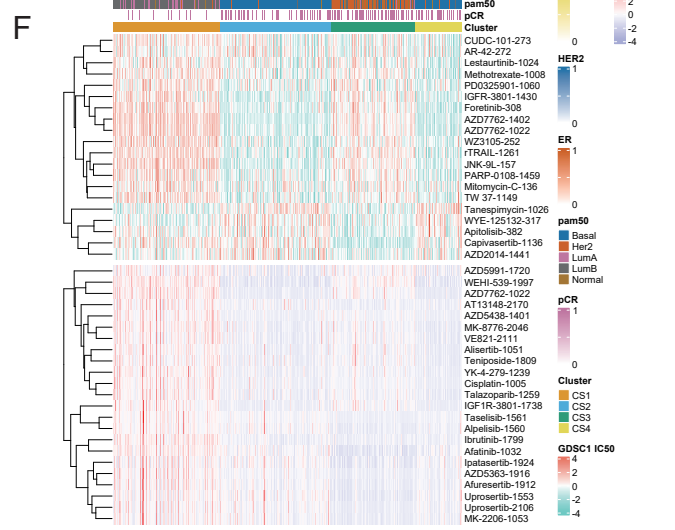

Supplement: Supplementary Figure 4 — Comparison of immunotherapy and drug response across breast cancer multimodal subtypes. (A) Expression of marker genes in the ISPY2 cohort’s four multimodal subtypes, identified using the NTP algorithm. (B) Comparison of 10 immunotherapy scores between pCR-positive and pCR-negative groups in the ISPY2 cohort, indicating a potentially better immunotherapy response in the pCR-positive group. (C) Comparison of 12 TIDE-derived immunotherapy-related scores between pCR-positive and pCR-negative groups across the four multimodal subtypes in the ISPY2 cohort. (D) Comparison of 12 immunotherapy-related scores among the four multimodal subtypes in the TCGA BRCA cohort, showing similar results to the ISPY2 cohort, with the CS4 subtype exhibiting a better immunotherapy response. (E) Comparison of 12 immunotherapy scores between pCR-positive (1) and pCR-negative (0) groups in the TCGA BRCA cohort. (F) Comparison of drug response scores among breast cancer multimodal subtypes in the ISPY2 cohort. Drug scores were obtained from GDSC1 (up) and GDSC2 (down), visualizing the top 20 drugs with significant differences among the four subtypes. Results indicate that the CS1 subtype is generally resistant to most drugs, consistent with our findings. [file DataSheet4.pdf]

# A

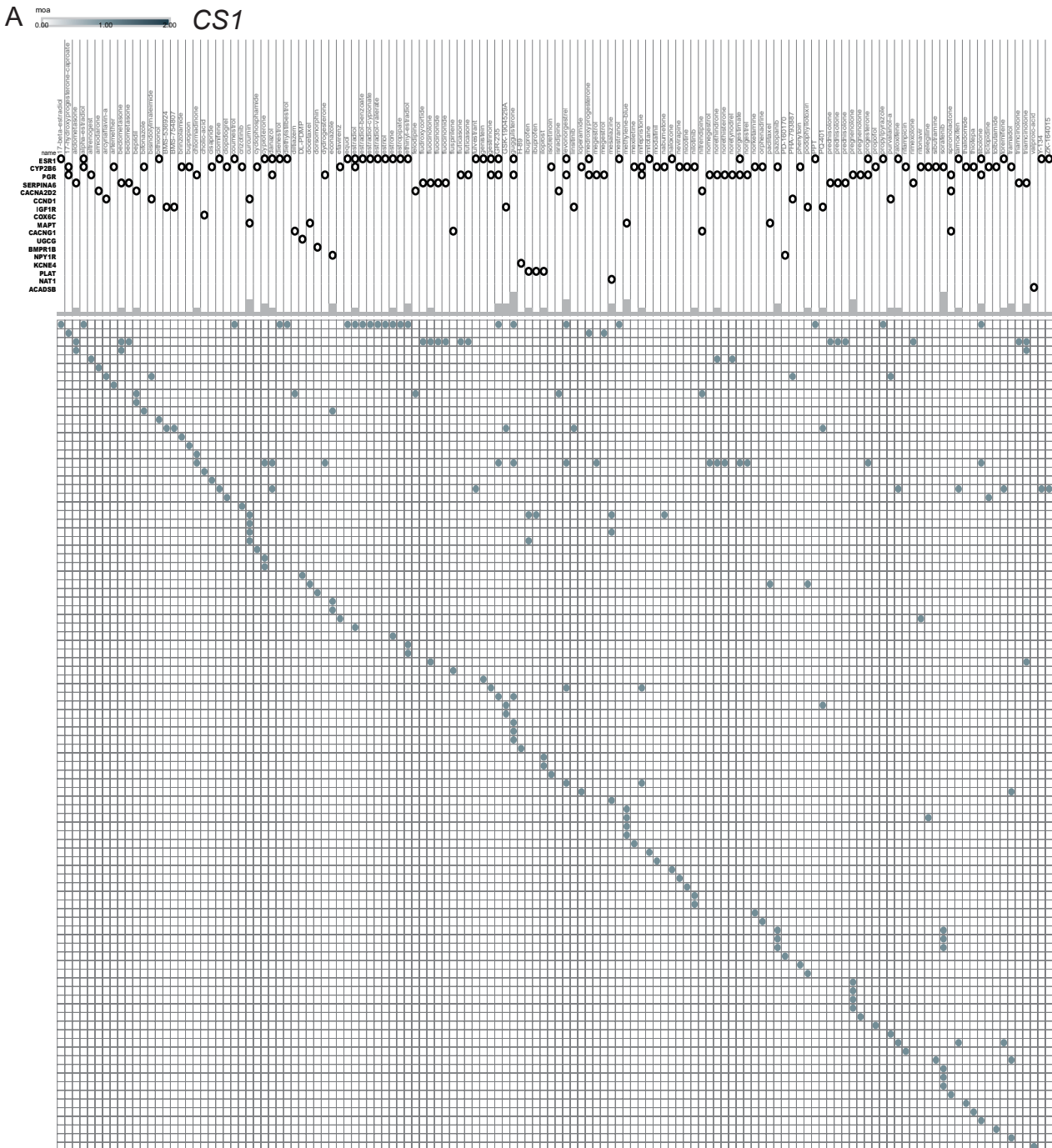

# B

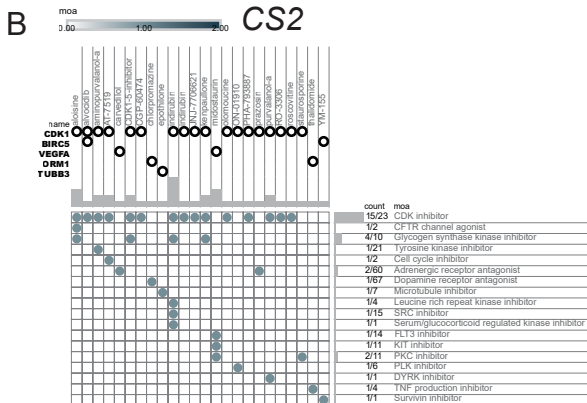

# C

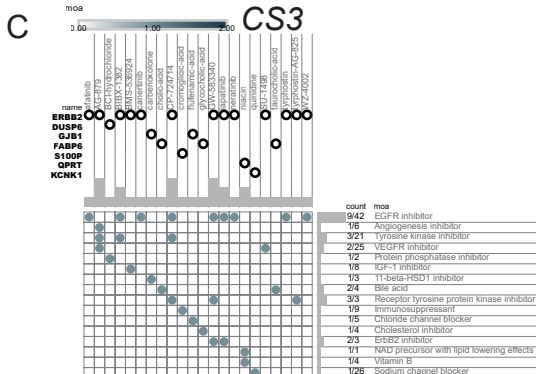

# D

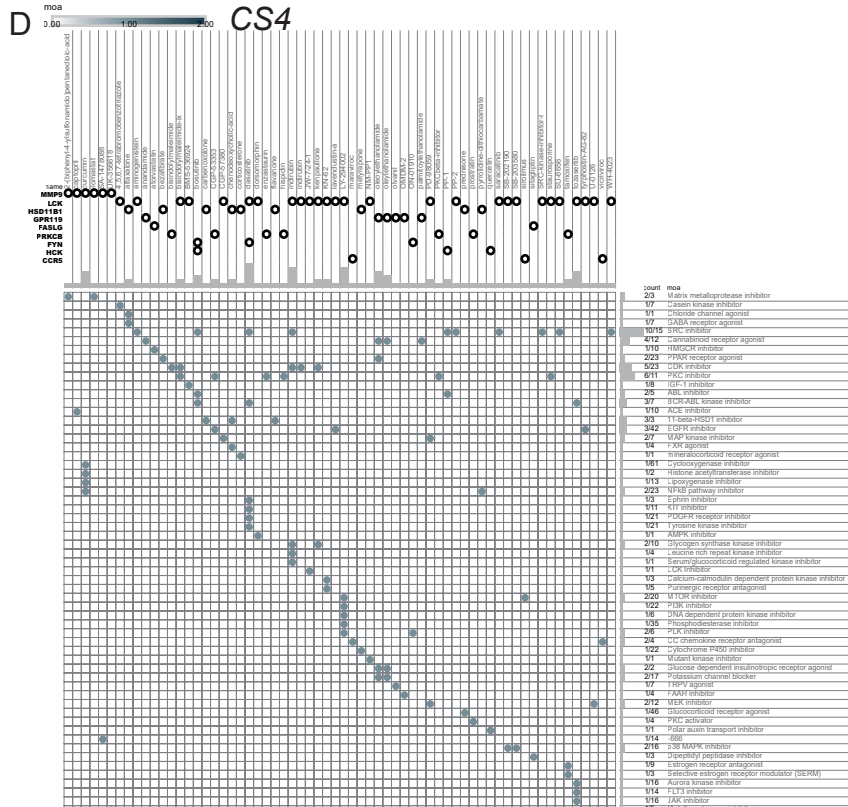

Supplement: Supplementary Figure 5 — Potential drug predictions for the four breast cancer multimodal subtypes. Using the Connectivity Map (cmap) platform, genes highly expressed in each of the four subtypes were input to identify potential therapeutic drugs and their mechanisms of action (MOA) specific to each subtype. (A) Predicted drugs and MOA for the CS1 subtype. (B) Predicted drugs and MOA for the CS2 subtype. (C) Predicted drugs and MOA for the CS3 subtype. (D) Predicted drugs and MOA for the CS4 subtype. This analysis highlights the unique molecular profiles of each subtype and suggests potential targeted therapies tailored to these profiles, offering insights into personalized treatment strategies for breast cancer patients. [file DataSheet5.pdf]

## Validation: TCGA BRCA cohort

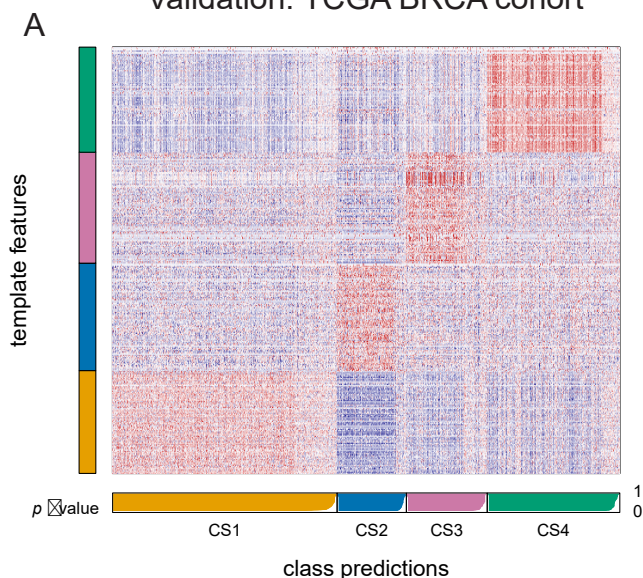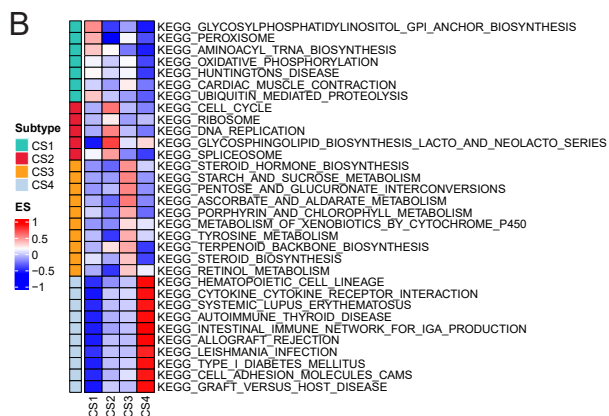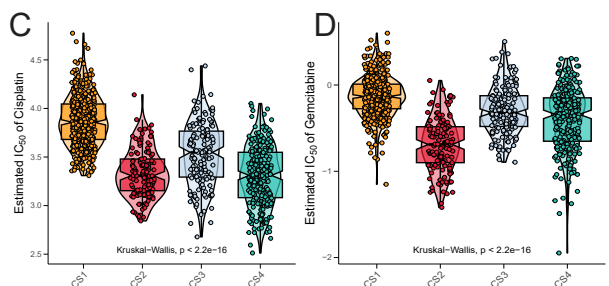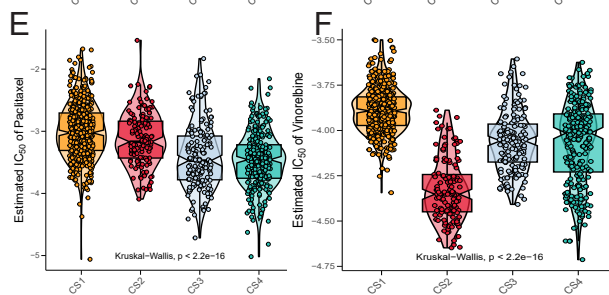

## Validation: Metabrick BRCA cohort

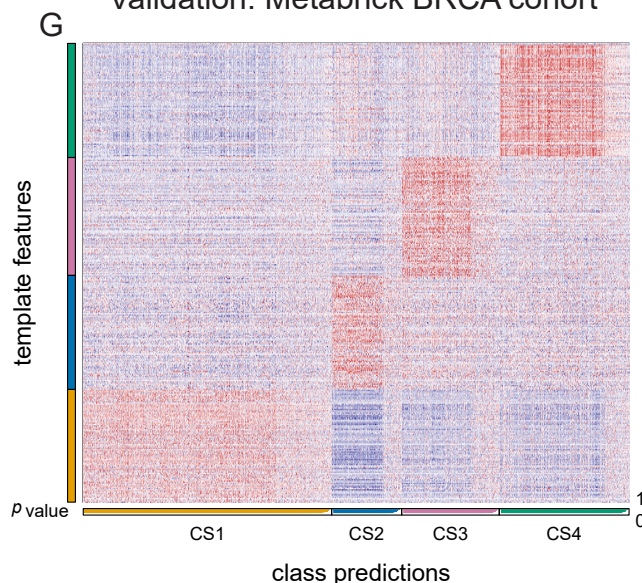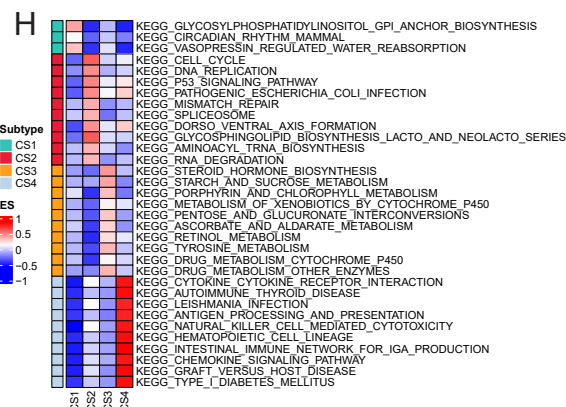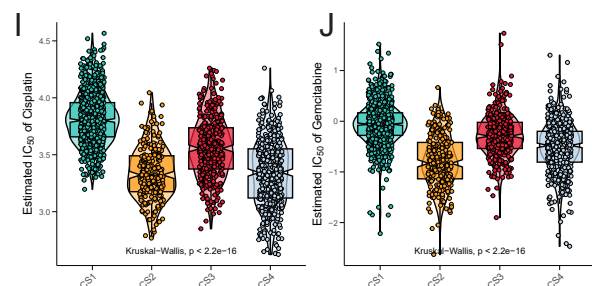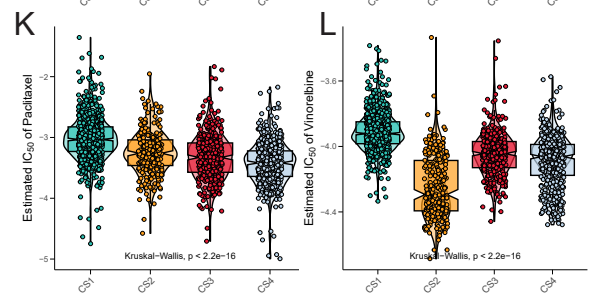

Supplement: Supplementary Figure 6 — Validation of molecular and drug response characteristics across breast cancer multimodal subtypes. (A–F) Validation of pathway activity and IC50 scores for four common chemotherapeutic drugs in the four subtypes within the TCGA BRCA cohort. (G–L) Validation of pathway activity and IC50 scores for the same chemotherapeutic drugs in the four subtypes within the Metabrick BRCA cohort. The analyses demonstrate consistent findings across both validation cohorts, underscoring the robustness of the molecular and drug response differences observed among the breast cancer multimodal subtypes. These results provide further evidence for the distinct biological and therapeutic profiles of each subtype, reinforcing the potential for subtype-specific treatment strategies in breast cancer. [file DataSheet6.pdf]

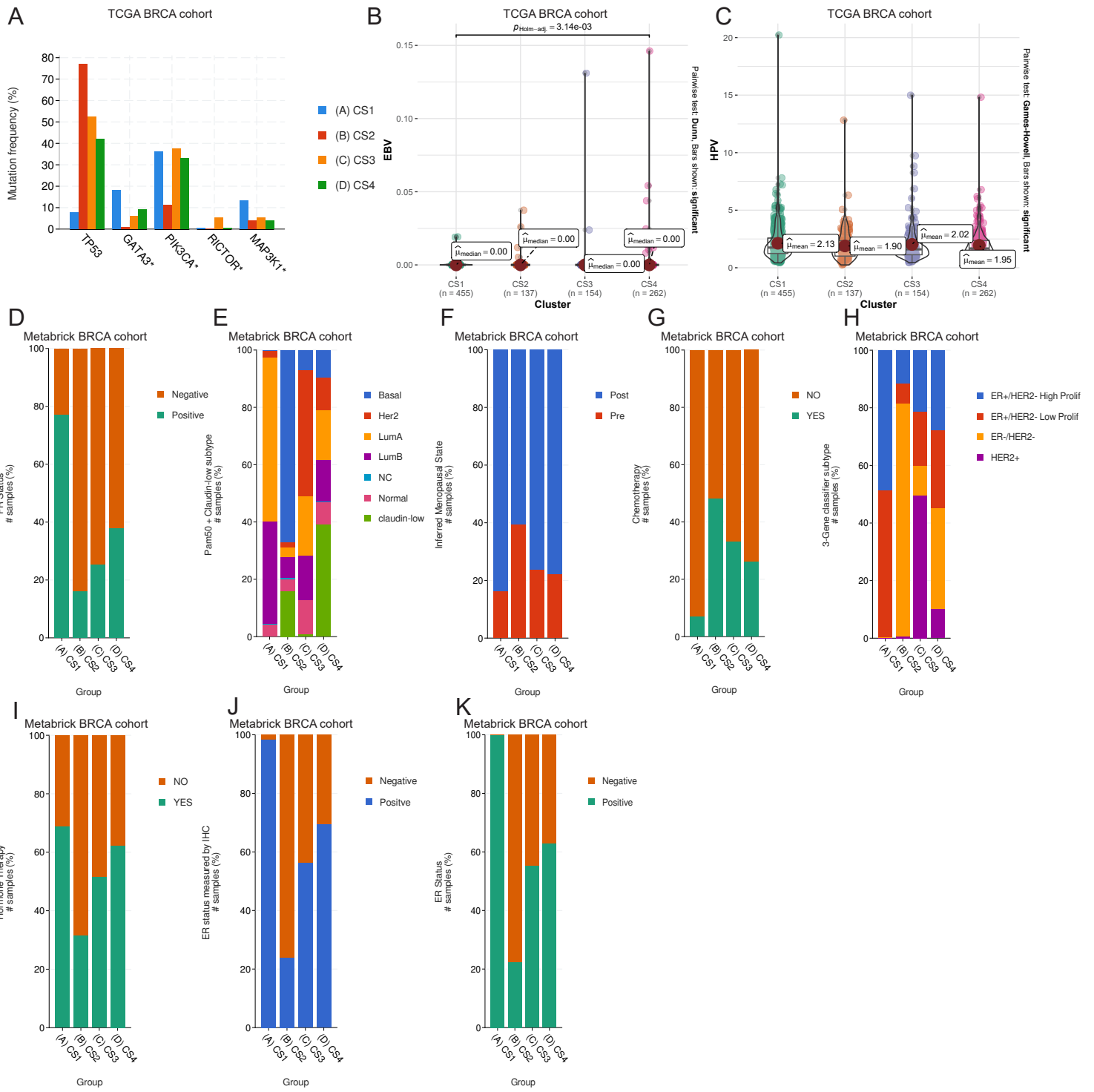

Supplement: Supplementary Figure 7 — Comparative analysis of four breast cancer multimodal subtypes in the TCGA-BRCA and Metabric-BRCA cohorts. (A) Mutation frequency comparison of five significantly different genes (TP53, GATA3, PIK3CA, RICTOR, MAP3K1) within the TCGA-BRCA cohort. (B) Comparison of EBV virus scores across the four multimodal subtypes in the TCGA-BRCA cohort, showing statistically significant differences. (C) Comparison of HPV virus scores across the four multimodal subtypes in the TCGA-BRCA cohort, with no statistically significant differences observed. (D–K) Comparative analysis of PR Status, Pam50+Claudin-low subtype, Inferred Menopausal State, Chemotherapy response, 3-Gene classifier subtype, Hormone Therapy response, ER Status measured by IHC, and overall ER Status across the four multimodal subtypes within the Metabric-BRCA cohort. These comparisons collectively provide a comprehensive understanding of the distinct molecular and clinical characteristics associated with each breast cancer multimodal subtype, reinforcing the robustness and clinical relevance of the proposed subtype classification. [file DataSheet7.pdf]

A

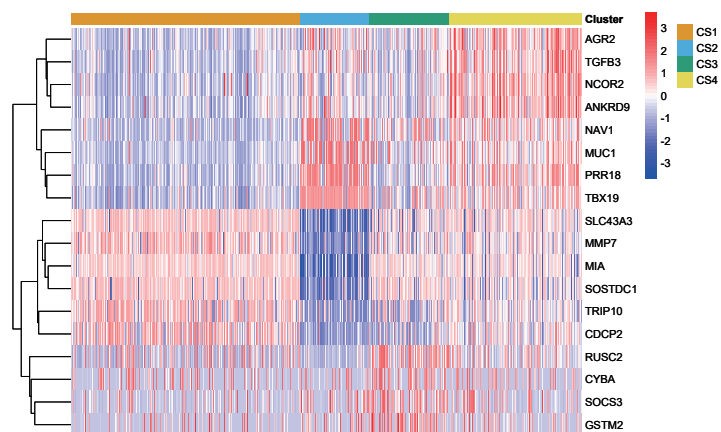

B

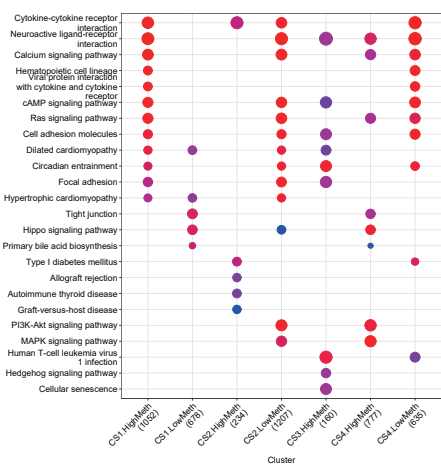

C

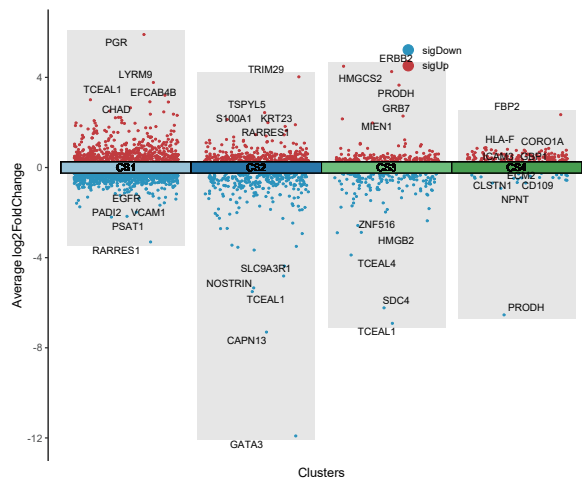

D

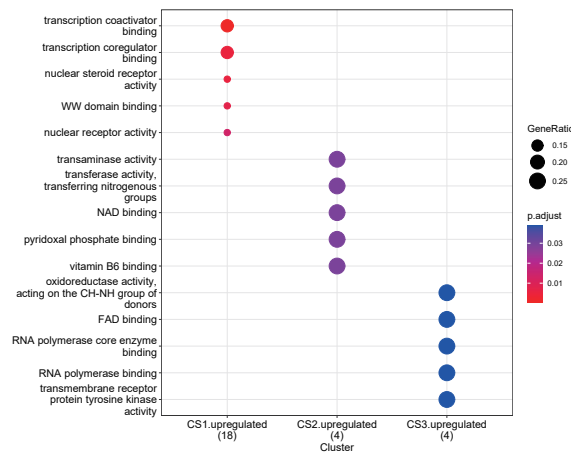

E

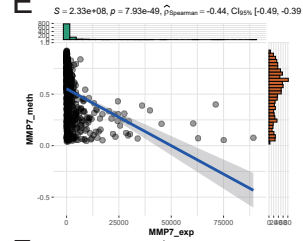

F

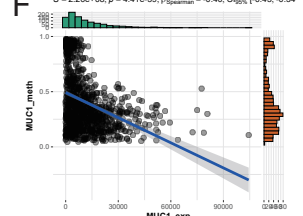

G

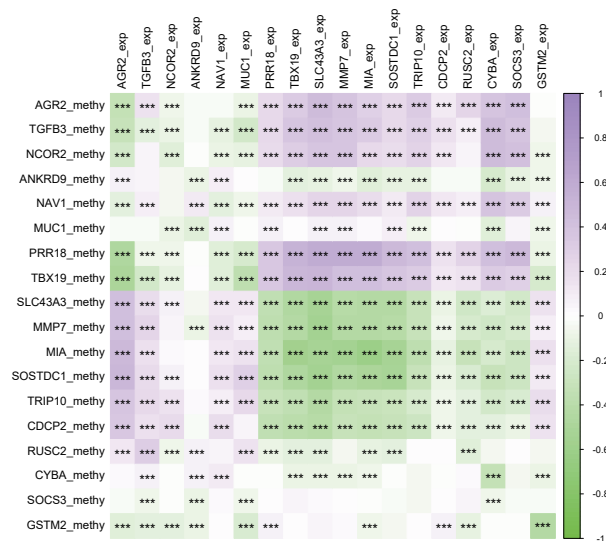

H

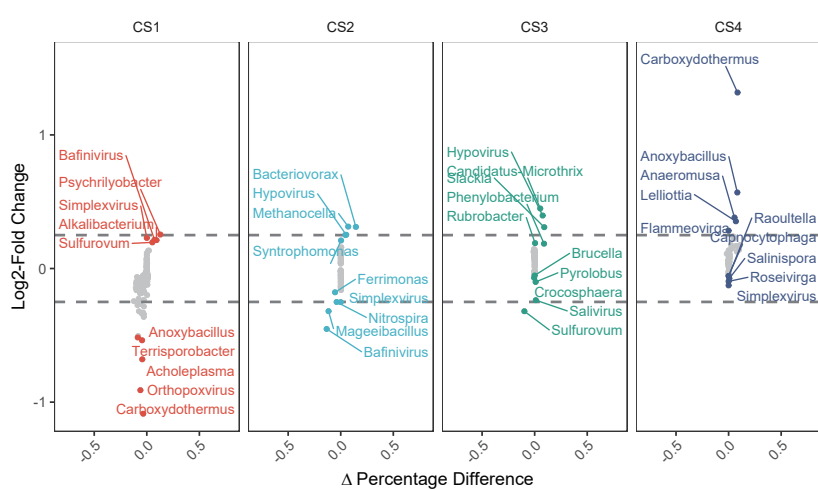

Supplement: Supplementary Figure 8 — Differential analysis of gene methylation, proteomics, and microbiomics across four breast cancer multimodal subtypes. The TCGA-BRCA cohort, possessing comprehensive multimodal data, was utilized to compare the four breast cancer multimodal subtypes across multiple molecular modalities. (A) Differential gene methylation analysis across the four breast cancer multimodal subtypes within the TCGA-BRCA cohort. The top 20 genes exhibiting significant methylation differences were selected, with the average beta value of all methylation sites for each gene used to represent its methylation level. (B) Functional enrichment analysis of differentially methylated genes. Highly methylated genes in the CS1 subtype were predominantly enriched in inflammation-related signaling pathways, suggesting suppression of these pathways in CS1. Conversely, the CS2 and CS4 subtypes showed high methylation in genes associated with inflammation-related pathways, indicating activation of these pathways. (C, D) Differential protein expression analysis and functional enrichment of differentially expressed proteins within the TCGA-BRCA cohort. (E, F) Negative correlation between gene expression and methylation levels of MMP7 and MUC1, respectively, illustrating the regulatory effect of methylation on the expression of these genes. (G) Significant negative correlation between gene expression and high methylation levels within the TCGA-BRCA cohort. (H) Differences in intratumoral microbiome composition across the four breast cancer multimodal subtypes within the TCGA-BRCA cohort (P<0.05 *, P<0.01 **, P<0.001 ***, P<0.0001 ****). [file DataSheet8.pdf]

A

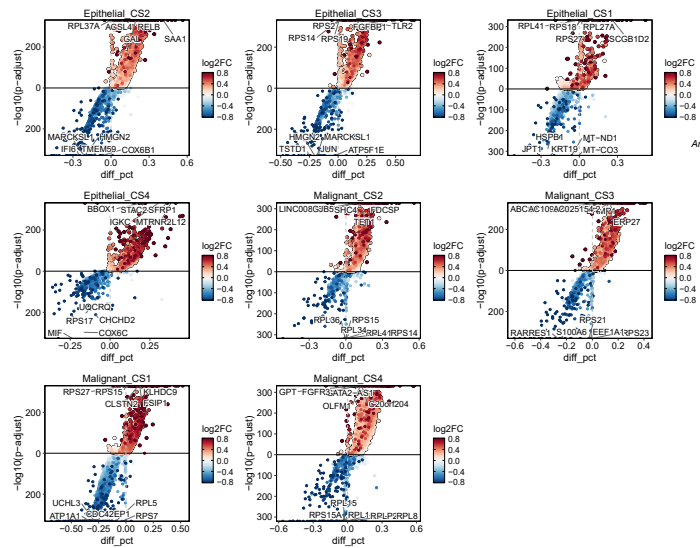

B

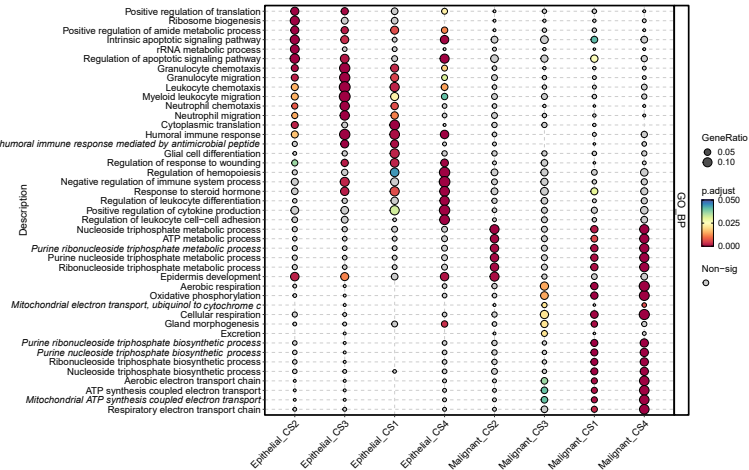

C

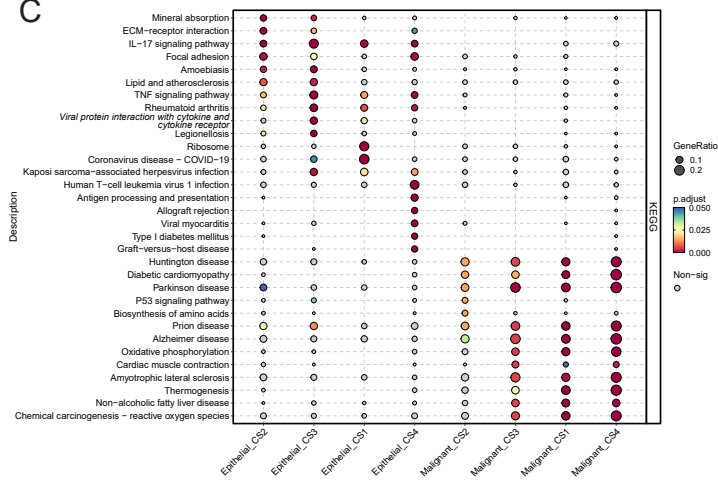

Supplement: Supplementary Figure 9 — Comparative analysis of the tumor microenvironment at the single-cell level across four breast cancer multimodal subtypes. (A) Volcano plots of differential expression analyses for Epithelial_CS1, Epithelial_CS2, Epithelial_CS3, Epithelial_CS4, Malignant_CS1, Malignant_CS2, Malignant_CS3, and Malignant_CS4. (B, C) Functional enrichment analysis results of highly expressed genes in the above eight cell subgroups, utilizing GO_BP and KEGG databases. [file DataSheet9.pdf]

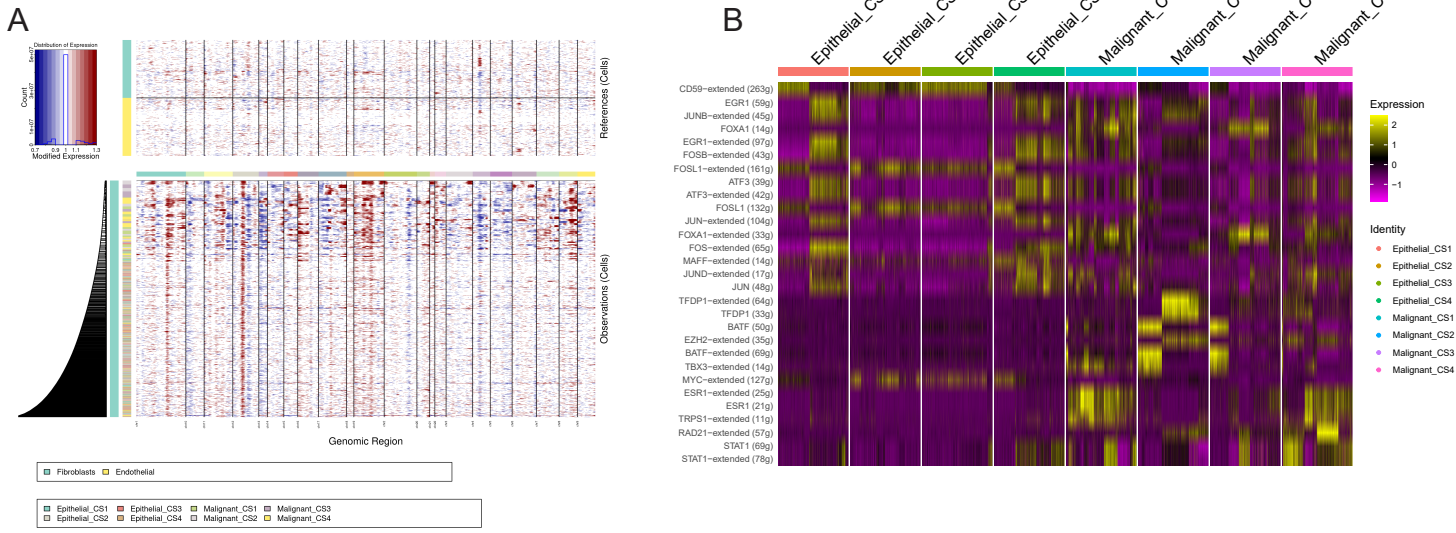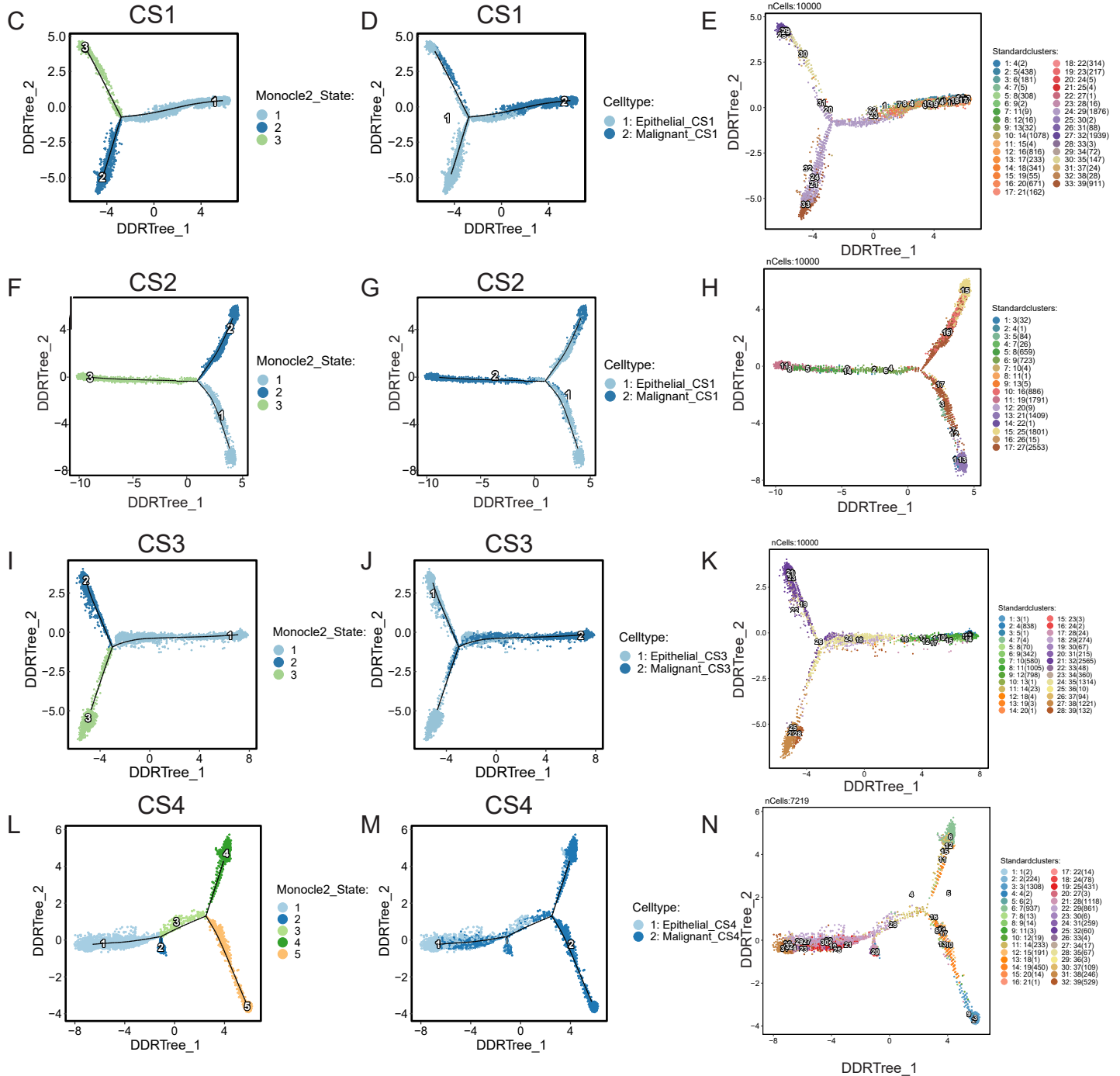

Supplement: Supplementary Figure 10 — Copy number variation, transcription factor analysis, and tumor evolution analysis in four breast cancer multimodal subtypes. (A) Copy number variation (CNV) analysis grouped by the four breast cancer multimodal subtypes, illustrating the distinct CNV profiles among the subtypes. (B) Transcription factor (TF) enrichment analysis for the eight cell subgroups (Epithelial_CS1, Epithelial_CS2, Epithelial_CS3, Epithelial_CS4, Malignant_CS1, Malignant_CS2, Malignant_CS3, and Malignant_CS4), highlighting key TFs driving the molecular characteristics of each subgroup. (C–E) Cell differentiation trajectory from epithelial cells to malignant cells in the CS1 subtype, depicting the stepwise evolution and key transitional states. (F–H) Cell differentiation trajectory from epithelial cells to malignant cells in the CS2 subtype, demonstrating the progression and cellular changes characteristic of this subtype. (I–K) Cell differentiation trajectory from epithelial cells to malignant cells in the CS3 subtype, showing the specific evolutionary pathway and intermediate stages. (L–N) Cell differentiation trajectory from epithelial cells to malignant cells in the CS4 subtype, outlining the transformation process and unique features of this subtype. These analyses provide comprehensive insights into the genetic alterations, transcriptional regulation, and evolutionary dynamics of breast cancer multimodal subtypes. The CNV analysis reveals subtype-specific genomic changes, while the transcription factor enrichment highlights the regulatory networks underlying each cell subgroup. The differentiation trajectories map the complex pathways through which epithelial cells evolve into malignant cells, offering a detailed view of tumor progression and potential targets for therapeutic intervention. [file DataSheet10.pdf]

A

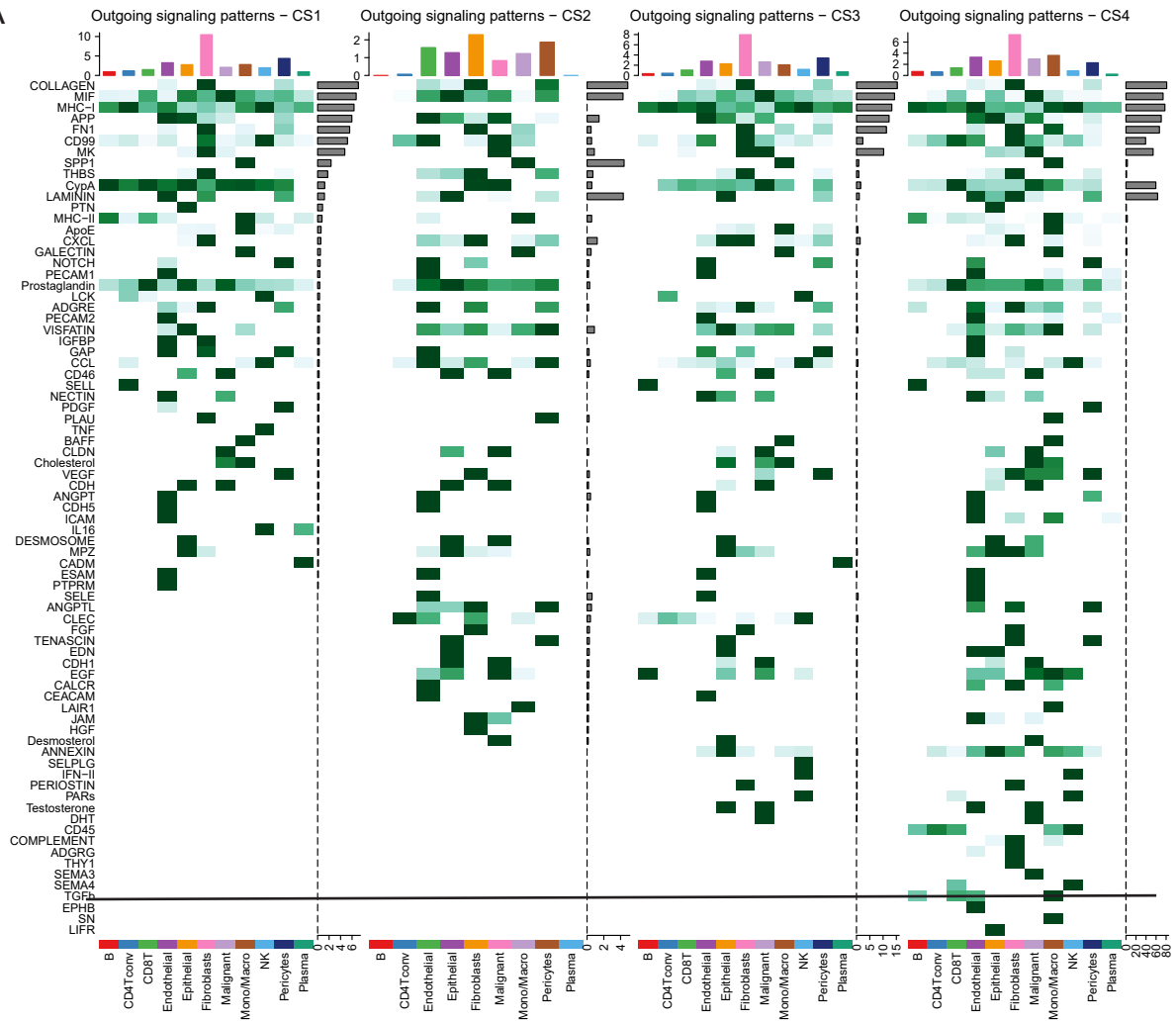

B

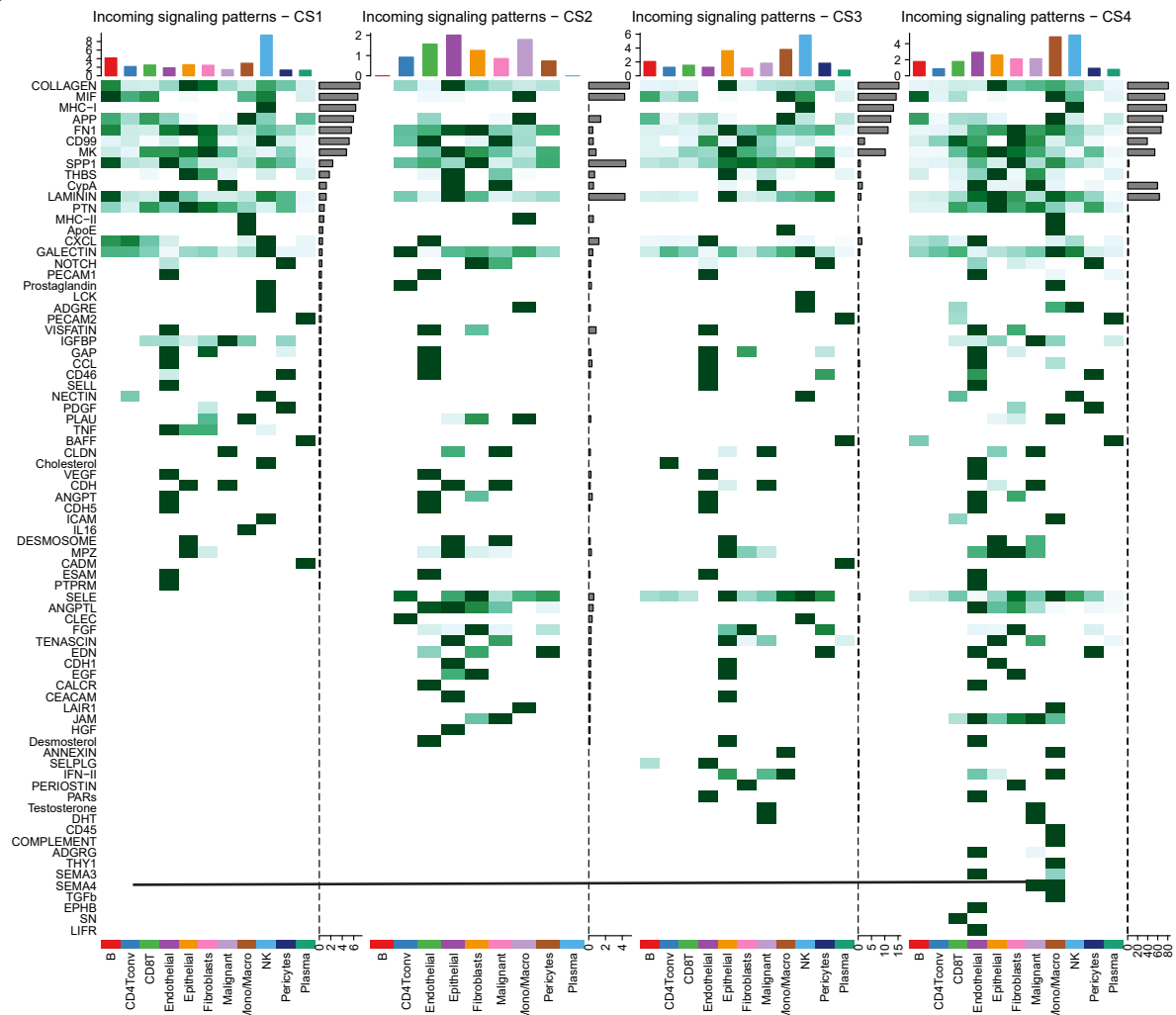

Supplement: Supplementary Figure 11 — Incoming and outgoing signaling patterns in four breast cancer multimodal subtypes. (A) Incoming signaling patterns of the four breast cancer multimodal subtypes, detailing the signals received by each of the 11 cell types within the tumor microenvironment. (B) Outgoing signaling patterns of the four breast cancer multimodal subtypes, illustrating the signals sent by each of the 11 cell types within the tumor microenvironment. These figures provide a detailed analysis of the directional flow of cellular communications in the tumor microenvironment of different breast cancer multimodal subtypes. The incoming signaling patterns (A) highlight which cell types are primarily receiving signals and how these patterns vary across subtypes. The outgoing signaling patterns (B) reveal the key signaling cell types and the nature of the signals they emit in each subtype. This comprehensive view of intercellular communication dynamics can help elucidate the complex interactions that drive tumor behavior and may identify critical nodes for therapeutic intervention in breast cancer. [file DataSheet11.pdf]

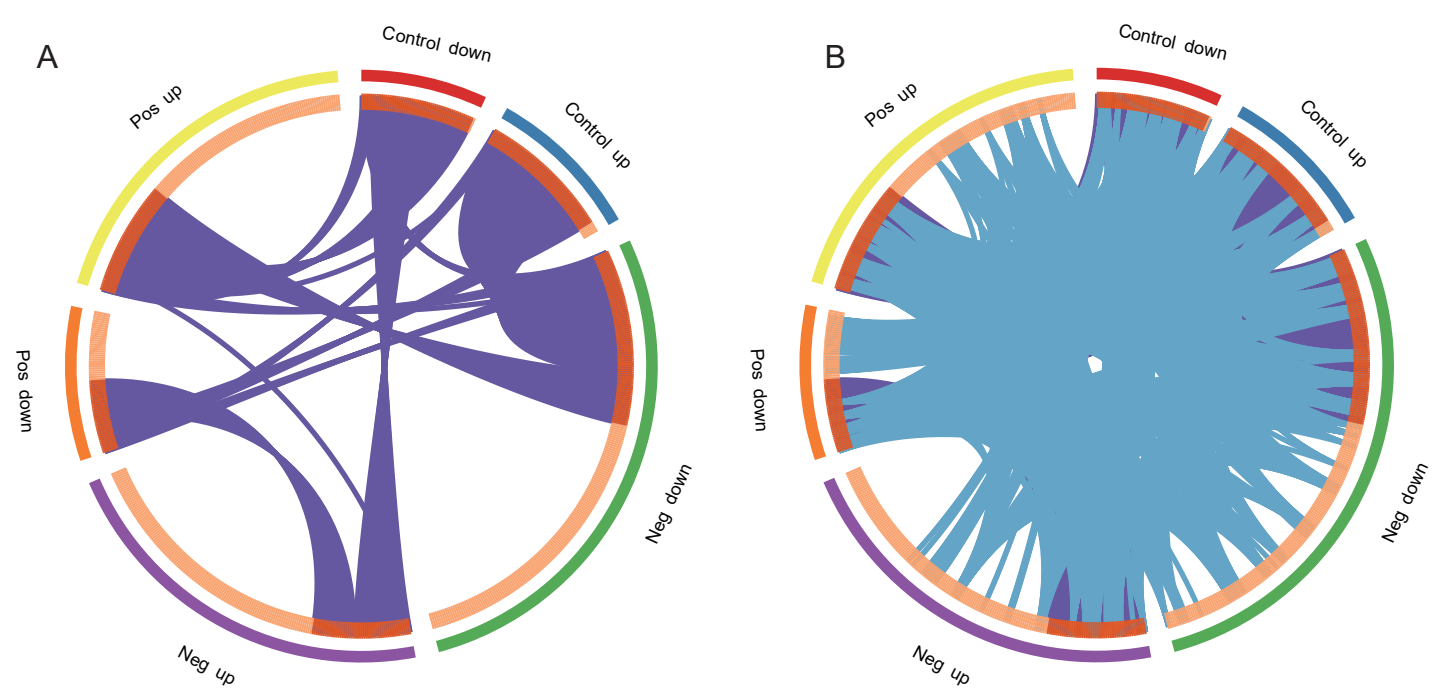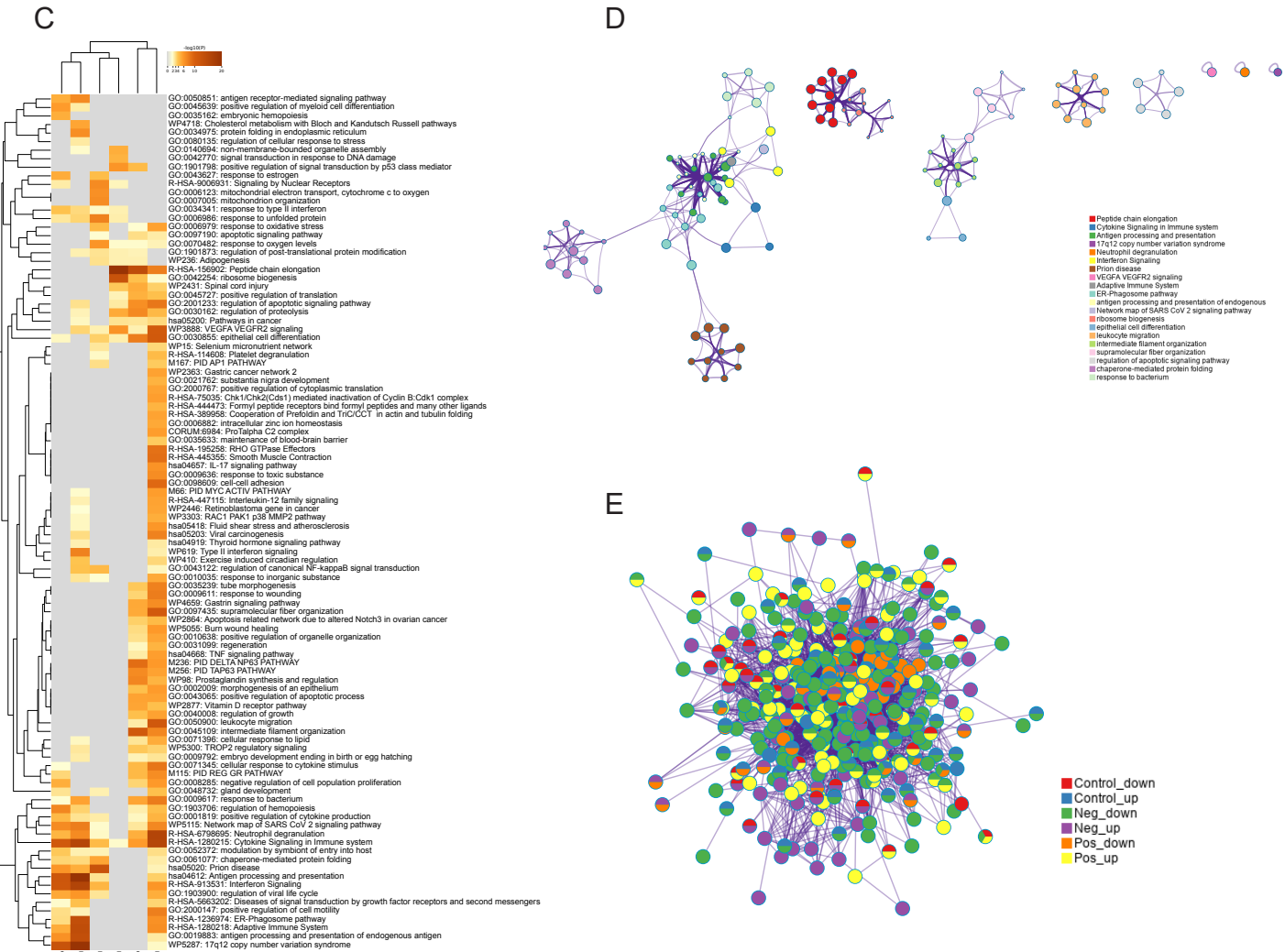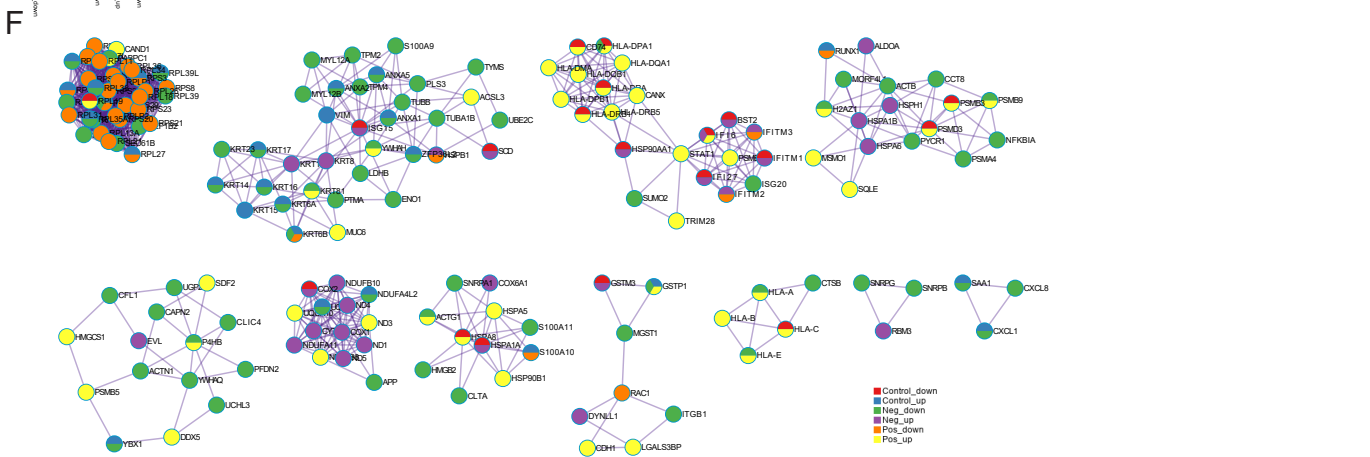

Supplement: Supplementary Figure 12 — Functional Enrichment Analysis of Differentially Expressed Genes in Malignant Cells using Metascape. (A, B) Comparative distribution of six differential gene sets: Pos up, Pos down, Neg up, Neg down, Ctrl up, and Ctrl down. (C) Functional enrichment analysis of the six differential gene sets mentioned above, highlighting key biological pathways and processes. (D) Construction of a functional enrichment network based on the differential gene sets, illustrating the interconnected pathways and their roles in cellular functions. (E) Protein-protein interaction (PPI) network constructed from the differential gene sets, showcasing the interactions between proteins encoded by the differentially expressed genes. (F) Core hub gene regulatory network identified using the MCODE algorithm on the PPI network of differentially expressed genes. These figures provide a detailed overview of the functional enrichment analysis performed on differentially expressed genes in malignant cells, specifically comparing pCR positive cells, pCR negative cells, and control cells. The comparative distribution of differential gene sets (A, B) reveals distinct patterns of gene expression associated with different drug response statuses. The functional enrichment analysis (C) highlights the key biological processes and pathways that are differentially regulated in these gene sets, offering insights into the underlying mechanisms of drug response. The functional enrichment network (D) and PPI network (E) provide a comprehensive view of the interconnected pathways and protein interactions, respectively, that are involved in the drug response. The identification of core hub gene regulatory networks using the MCODE algorithm (F) further pinpoints critical genes and interactions that may play pivotal roles in determining the drug response in malignant cells. These analyses are crucial for understanding the molecular basis of drug response variability in breast cancer and could potentially guide the deve [file DataSheet12.pdf]
