# Supplementary material for: Multimodal diagnostic models and subtype analysis for neoadjuvant therapy in breast cancer
Source: Front Immunol. 2025 Mar 18;16:1559200. doi: 10.3389/fimmu.2025.1559200 (PMC11958217; doi:10.3389/fimmu.2025.1559200)
Supplement: Supplementary Figure 2 — Performance of the model constructed using features selected via Lasso regression from three modalities (46 proteomics features, 60 transcriptomics features, and 42 radiomics features) combined with clinical features. The final model, obtained through another round of Lasso regression with 1000 permutations, includes 32 features. The performance metrics include AUC, Calibration curve, Precision-Recall, Lift curve, and Cumulative Gains. (A) Performance in the training set. (B) Performance in the validation set. [file DataSheet2.pdf]

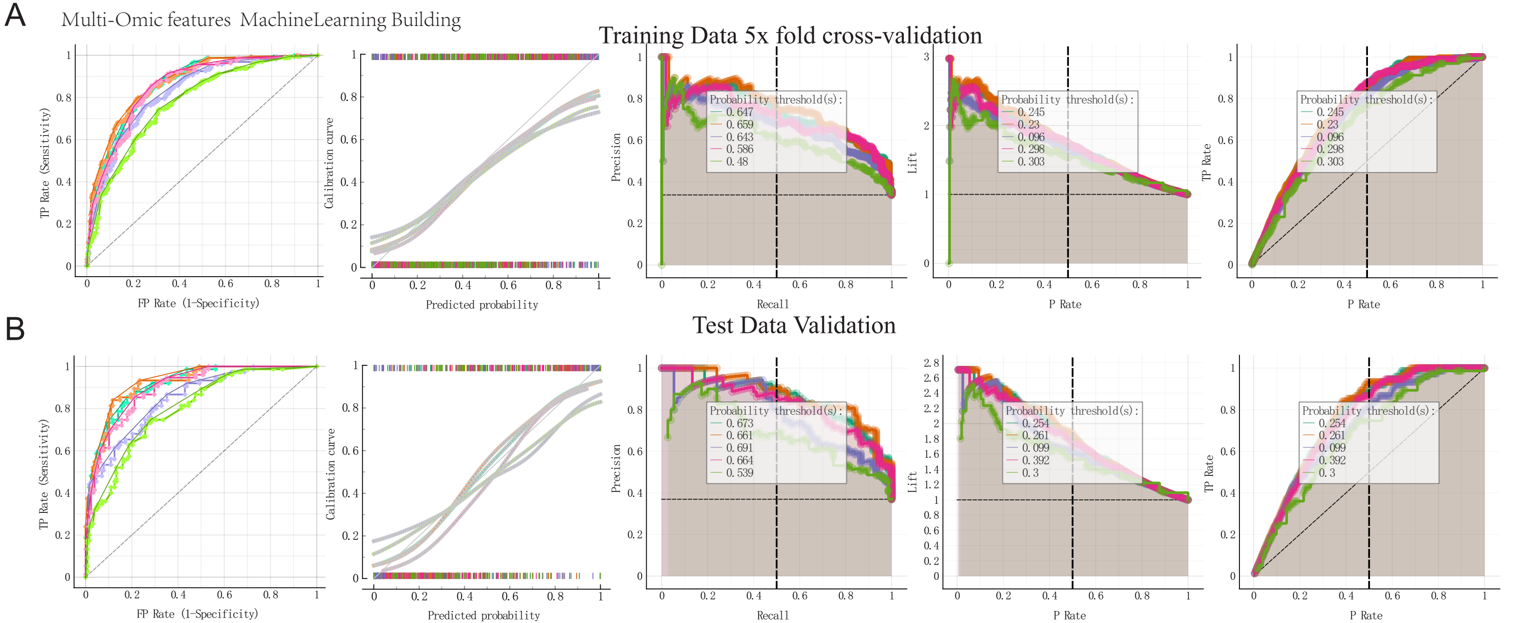
A
Multi-Omic features MachineLearning Building
Training Data 5x fold cross-validation
TP Rate (Sensitivity)
FP Rate (1-Specificity)
Calibration curve
Predicted probability
Precision
Recall
Probability threshold(s):
0.647
0.659
0.643
0.586
0.48
Lift
P Rate
Probability threshold(s):
0.245
0.23
0.096
0.298
0.303
TP Rate
P Rate
Probability threshold(s):
0.245
0.23
0.096
0.298
0.303
B
Test Data Validation
TP Rate (Sensitivity)
FP Rate (1-Specificity)
Calibration curve
Predicted probability
Precision
Recall
Probability threshold(s):
0.673
0.661
0.691
0.664
0.539
Lift
P Rate
Probability threshold(s):
0.254
0.261
0.099
0.392
0.3
TP Rate
P Rate
Probability threshold(s):
0.254
0.261
0.099
0.392
0.3
