# Supplementary material for: Multimodal diagnostic models and subtype analysis for neoadjuvant therapy in breast cancer
Source: Front Immunol. 2025 Mar 18;16:1559200. doi: 10.3389/fimmu.2025.1559200 (PMC11958217; doi:10.3389/fimmu.2025.1559200)
Supplement: Supplementary file 13 [file DataSheet13.pdf]

Sampling type: Stratified 5-fold Cross validation

Model	AUC	CA	F1	Prec	Recall	MCC
Logistic Regression	0.847	0.773	0.77	0.769	0.773	0.481
Ridge	0.858	0.777	0.774	0.773	0.777	0.491
Gradient Boosting	0.769	0.712	0.704	0.701	0.712	0.328
SVM	0.807	0.76	0.757	0.755	0.76	0.451
Random Forest	0.701	0.672	0.656	0.653	0.672	0.217

Sampling type: test on training data

Model	AUC	CA	F1	Prec	Recall	MCC
Logistic Regression	0.927	0.838	0.835	0.835	0.838	0.629
Ridge	0.932	0.84	0.838	0.838	0.84	0.635
Gradient Boosting	1	1	1	1	1	1
SVM	0.988	0.947	0.947	0.947	0.947	0.881
Random Forest	0.997	0.985	0.985	0.985	0.985	0.967

Sampling type: test on testing data

Model	AUC	CA	F1	Prec	Recall	MCC
Logistic Regression	0.876	0.793	0.79	0.79	0.793	0.547
Ridge	0.883	0.788	0.786	0.785	0.788	0.538
Gradient Boosting	0.789	0.734	0.728	0.728	0.734	0.413
SVM	0.814	0.739	0.734	0.734	0.739	0.425
Random Forest	0.721	0.69	0.668	0.679	0.69	0.293

Sampling type: Stratified 5-fold Cross validation
Target class: 0,1, show average over classes

Model	AUC	CA	F1	Prec	Recall	MCC
Logistic Regression	0.612	0.672	0.623	0.639	0.672	0.162
Ridge	0.645	0.684	0.639	0.659	0.684	0.203
Gradient Boosting	0.546	0.632	0.599	0.593	0.632	0.083
SVM	0.561	0.642	0.627	0.621	0.642	0.149
Random Forest	0.513	0.634	0.59	0.586	0.634	0.066

Sampling type: test on training data

Model	AUC	CA	F1	Prec	Recall	MCC
Logistic Regression	0.756	0.726	0.684	0.73	0.726	0.332
Ridge	0.759	0.731	0.693	0.732	0.731	0.345
Gradient Boosting	1	1	1	1	1	1
SVM	0.82	0.754	0.751	0.749	0.754	0.438
Random Forest	0.998	0.975	0.975	0.975	0.975	0.943

Sampling type: test on testing data

Model	AUC	CA	F1	Prec	Recall	MCC
Logistic Regression	0.734	0.675	0.631	0.667	0.675	0.243
Ridge	0.753	0.685	0.642	0.683	0.685	0.271
Gradient Boosting	0.653	0.65	0.631	0.632	0.65	0.202
SVM	0.656	0.65	0.649	0.647	0.65	0.243
Random Forest	0.592	0.621	0.595	0.594	0.621	0.122

Sampling type: Stratified 5-fold Cross validation
Target class: 0,1, show average over classes

**Model**	**AUC**	**CA**	**F1**	**Prec**	**Recall**	**MCC**
Logistic Regression	0.74	0.703	0.693	0.69	0.703	0.302
Ridge	0.739	0.701	0.69	0.688	0.701	0.296
Gradient Boosting	0.705	0.682	0.666	0.664	0.682	0.24
SVM	0.647	0.646	0.627	0.621	0.646	0.147
Random Forest	0.705	0.682	0.663	0.662	0.682	0.234

Sampling type: test on training data

**Model**	**AUC**	**CA**	**F1**	**Prec**	**Recall**	**MCC**
Logistic Regression	0.853	0.783	0.775	0.778	0.783	0.494
Ridge	0.856	0.804	0.797	0.801	0.804	0.545
Gradient Boosting	1	1	1	1	1	1
SVM	0.933	0.865	0.863	0.864	0.865	0.692
Random Forest	0.998	0.977	0.977	0.977	0.977	0.948

Sampling type: test on testing data

**Model**	**AUC**	**CA**	**F1**	**Prec**	**Recall**	**MCC**
Logistic Regression	0.762	0.675	0.658	0.661	0.675	0.263
Ridge	0.768	0.665	0.649	0.651	0.665	0.242
Gradient Boosting	0.698	0.665	0.643	0.649	0.665	0.233
SVM	0.682	0.64	0.62	0.621	0.64	0.178
Random Forest	0.638	0.67	0.631	0.657	0.67	0.231

Sampling type: Stratified 5-fold Cross validation
Target class: 0,1, show average over classes

Model	AUC	CA	F1	Prec	Recall	MCC
Logistic Regression	0.703	0.705	0.68	0.688	0.705	0.282
Ridge	0.691	0.684	0.662	0.663	0.684	0.233
Gradient Boosting	0.612	0.619	0.611	0.606	0.619	0.117
SVM	0.645	0.663	0.647	0.643	0.663	0.196
Random Forest	0.621	0.606	0.608	0.609	0.606	0.126

Sampling type: test on training data

Model	AUC	CA	F1	Prec	Recall	MCC
Logistic Regression	0.758	0.718	0.695	0.704	0.718	0.317
Ridge	0.763	0.722	0.701	0.709	0.722	0.33
Gradient Boosting	0.996	0.962	0.962	0.962	0.962	0.915
SVM	0.774	0.745	0.74	0.739	0.745	0.413
Random Forest	0.949	0.886	0.888	0.893	0.886	0.758

Sampling type: test on testing data

Model	AUC	CA	F1	Prec	Recall	MCC
Logistic Regression	0.696	0.64	0.607	0.616	0.64	0.16
Ridge	0.699	0.675	0.658	0.661	0.675	0.263
Gradient Boosting	0.645	0.606	0.592	0.587	0.606	0.112
SVM	0.649	0.665	0.663	0.662	0.665	0.273
Random Forest	0.643	0.591	0.594	0.597	0.591	0.135

Sampling type: Stratified 5-fold Cross validation
Target class: 0,1, show average over classes

Model	AUC	CA	F1	Prec	Recall	MCC
Logistic Regression	0.858	0.777	0.775	0.773	0.777	0.492
Ridge	0.865	0.802	0.801	0.8	0.802	0.552
Gradient Boosting	0.821	0.762	0.759	0.758	0.762	0.458
SVM	0.847	0.758	0.756	0.754	0.758	0.449
Random Forest	0.773	0.731	0.71	0.719	0.731	0.352

Sampling type: test on training data

Model	AUC	CA	F1	Prec	Recall	MCC
Logistic Regression	0.904	0.825	0.823	0.822	0.825	0.6
Ridge	0.906	0.829	0.828	0.828	0.829	0.614
Gradient Boosting	1	1	1	1	1	1
SVM	0.975	0.941	0.941	0.942	0.941	0.869
Random Forest	0.999	0.983	0.983	0.984	0.983	0.963

Sampling type: test on testing data

Model	AUC	CA	F1	Prec	Recall	MCC
Logistic Regression	0.91	0.823	0.819	0.822	0.823	0.612
Ridge	0.917	0.823	0.818	0.822	0.823	0.611
Gradient Boosting	0.843	0.778	0.773	0.775	0.778	0.512
SVM	0.893	0.808	0.807	0.807	0.808	0.585
Random Forest	0.794	0.724	0.716	0.717	0.724	0.387
