# Supplementary material for: Multimodal diagnostic models and subtype analysis for neoadjuvant therapy in breast cancer
Source: Front Immunol. 2025 Mar 18;16:1559200. doi: 10.3389/fimmu.2025.1559200 (PMC11958217; doi:10.3389/fimmu.2025.1559200)
Supplement: Supplementary file 14 [file DataSheet14.pdf]

Feature	Mean	Std	
ABCC11	0.0101705	0.000820652	1000
IL20	0.00569425	0.00100014	1000
CGA	0.00105278	0.000382137	1000
WNT6	0.000922146	0.000188917	1000
LONRF2	0.000672398	0.000237398	1000
RARRES1	0.000651266	0.000162946	1000
KLHDC7B	0.000449546	0.000197737	1000
TMEM45B	0.000295855	7.60E-05	1000
MAPT	0.000288171	7.76E-05	1000
SLC22A23	0.000263196	0.000246819	1000
TPSAB1	0.000105663	7.68E-05	1000
EZH2	4.80E-05	4.42E-05	1000
CCL18	4.61E-05	3.29E-05	1000
MARCO	4.03E-05	3.76E-05	1000
SCNN1A	4.03E-05	3.18E-05	1000
CDH3	4.03E-05	4.23E-05	1000
DOK7	3.07E-05	1.54E-05	1000
LRG1	3.07E-05	2.61E-05	1000
ADAMDEC1	2.88E-05	2.19E-05	1000
AQP5	2.50E-05	3.19E-05	1000
LOC647478	2.31E-05	2.89E-05	1000
RGS22	1.92E-05	1.22E-05	1000
HEY2	1.73E-05	1.27E-05	1000
PGR	1.34E-05	1.44E-05	1000
LAIR2	1.34E-05	7.68E-06	1000
SOX11	1.15E-05	1.12E-05	1000
NPNT	1.15E-05	1.41E-05	1000
C1orf64	1.15E-05	1.86E-05	1000
STX17	9.61E-06	1.92E-05	1000
NMU	7.68E-06	1.12E-05	1000
CD27	7.68E-06	3.84E-06	1000
SERPINA5	5.76E-06	7.68E-06	1000
IL22RA2	5.76E-06	1.15E-05	1000
KCNJ3	3.84E-06	7.68E-06	1000
CTSC	3.84E-06	4.71E-06	1000
PPP1R14C	3.84E-06	4.71E-06	1000
FUOM	3.84E-06	7.68E-06	1000
CENPW	3.84E-06	7.68E-06	1000
FGFBP1	3.84E-06	4.71E-06	1000
PSMD3	1.92E-06	3.84E-06	1000
FUT3	1.92E-06	3.84E-06	1000
PHACS	1.92E-06	3.84E-06	1000
PDE4B	1.92E-06	3.84E-06	1000
CCND1	0	0	1000
ESR1	0	0	1000
TXNDC5	0	0	1000
ACADSB	0	0	1000
SCUBE2	0	0	1000
GZMB	0	0	1000
THSD4	0	0	1000
SLC39A6	0	0	1000
ABCA3	0	0	1000
PCP2	0	0	1000

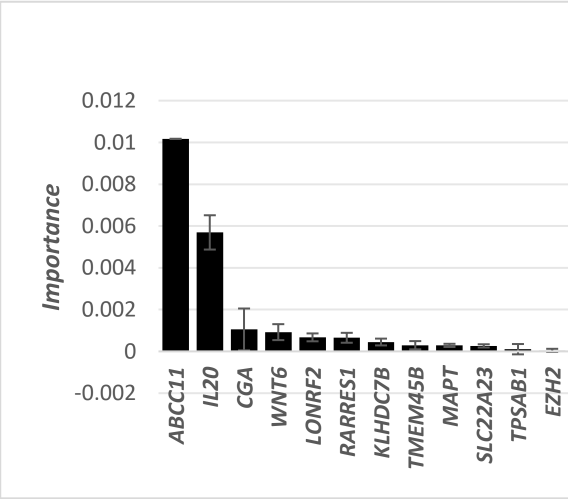

LRRC56	0	0	1000
LPPR3	0	0	1000
ACTR3	0	0	1000
WARS	0	0	1000
CCDC74B	0	0	1000
CHST11	0	0	1000
ZSWIM5	0	0	1000
ADCY9	0	0	1000
AI675062	0	0	1000
MED13L	0	0	1000
C3orf18	0	0	1000
FAM134B	0	0	1000
DBNDD2	0	0	1000
CXCL9	0	0	1000
IGF1R	0	0	1000
GAMT	0	0	1000
TMPRSS6	0	0	1000
PCSK4	0	0	1000
AL133644	0	0	1000
CD38	0	0	1000
IRS1	0	0	1000
TMSB10	0	0	1000
C6orf211	0	0	1000
SLC22A5	0	0	1000
RERG	0	0	1000
RIMS4	0	0	1000
GBP5	0	0	1000
AIM2	0	0	1000
GBP1	0	0	1000
HEXIM1	0	0	1000
CXCL13	0	0	1000
IDO1	0	0	1000
CDCA7	0	0	1000
CA12	0	0	1000
NKAIN1	0	0	1000
SLAMF8	0	0	1000
NRIP1	0	0	1000
CCNE1	0	0	1000
ZBED2	0	0	1000
GATA3	0	0	1000
PPM1J	0	0	1000
PARD6B	0	0	1000
ADCY1	0	0	1000
BBS1	0	0	1000
SPR	0	0	1000
MIEN1	0	0	1000
IL21R	0	0	1000
CRIPAK	0	0	1000
TPBG	0	0	1000
ZMYND10	0	0	1000
PTP4A2	0	0	1000
CCDC170	0	0	1000
FAM63A	0	0	1000
SLAMF7	0	0	1000

SYK	0	0	1000
HAPLN3	0	0	1000
SYBU	0	0	1000
CXCL10	0	0	1000
CLSTN2	0	0	1000
AMFR	0	0	1000
P2RY6	0	0	1000
WWP1	0	0	1000
LRRC49	0	0	1000
ANKS3	0	0	1000
C5orf30	0	0	1000
DNAJC12	0	0	1000
PLCD4	0	0	1000
CCL8	0	0	1000
CORO1C	0	0	1000
FBXL16	0	0	1000
MELK	0	0	1000
ERBB2	0	0	1000
CCDC71L	0	0	1000
MSANTD3	0	0	1000
CCL13	0	0	1000
BAI2	0	0	1000
SYTL4	0	0	1000
LRRC73	0	0	1000
FAM196A	0	0	1000
PDZK1	0	0	1000
CYTIP	0	0	1000
MCM6	0	0	1000
CCL4	0	0	1000
STC2	0	0	1000
PSAT1	0	0	1000
KCNF1	0	0	1000
SMIM22	0	0	1000
LCP1	0	0	1000
ELP2	0	0	1000
DNALI1	0	0	1000
TMEM123	0	0	1000
PDPK1	0	0	1000
IGFALS	0	0	1000
KIFC2	0	0	1000
TMSB15A	0	0	1000
KCTD6	0	0	1000
ECI1	0	0	1000
LOC102724	0	0	1000
PLIN5	0	0	1000
KIAA1467	0	0	1000
KIF3A	0	0	1000
ABLIM3	0	0	1000
NAT1	0	0	1000
LMO4	0	0	1000
FAM49A	0	0	1000
CTSF	0	0	1000
STK36	0	0	1000
TCL1A	0	0	1000

CHAD	0	0	1000
PGAP3	0	0	1000
NUDT6	0	0	1000
DAPP1	0	0	1000
UBD	0	0	1000
CXCL1	0	0	1000
MAST4	0	0	1000
AKR7L	0	0	1000
IL6ST	0	0	1000
ICAM1	0	0	1000
TBC1D9	0	0	1000
HTR7	0	0	1000
C1QC	0	0	1000
SPOPL	0	0	1000
SIRT3	0	0	1000
FAM174A	0	0	1000
CELSR2	0	0	1000
CXXC5	0	0	1000
SOD2	0	0	1000
MGC34796	0	0	1000
NPY1R	0	0	1000
STK17B	0	0	1000
MSL3P1	0	0	1000
KLHDC9	0	0	1000
RSPH1	0	0	1000
TCAP	0	0	1000
TMEM128	0	0	1000
ZC3H6	0	0	1000
SLC25A35	0	0	1000
RABEP1	0	0	1000
GLIPR2	0	0	1000
OCEL1	0	0	1000
MKL2	0	0	1000
ISG20	0	0	1000
RGS11	0	0	1000
CXCR3	0	0	1000
P4HTM	0	0	1000
GRB7	0	0	1000
CIRBP	0	0	1000
PDSS1	0	0	1000
FCGR1B	0	0	1000
NEDD4L	0	0	1000
KRT86	0	0	1000
PRRT2	0	0	1000
CAMLG	0	0	1000
IFI30	0	0	1000
ZNF425	0	0	1000
RMND1	0	0	1000
USP35	0	0	1000
POU2AF1	0	0	1000
DPEP3	0	0	1000
HLA-E	0	0	1000
GREB1	0	0	1000
ANKRD50	0	0	1000

CXCR4	0	0	1000
EIF3J-AS1	0	0	1000
PDCD1	0	0	1000
STAT1	0	0	1000
GNLY	0	0	1000
HLA-DRB1	0	0	1000
SLC19A2	0	0	1000
TNFSF13B	0	0	1000
PPP1R14B	0	0	1000
TAP1	0	0	1000
CD53	0	0	1000
IGLL1	0	0	1000
ICOS	0	0	1000
CXCR6	0	0	1000
BORA	0	0	1000
COX6C	0	0	1000
GBP4	0	0	1000
ANKHD1-EI	0	0	1000
AI862477	0	0	1000
SLTM	0	0	1000
COL4A4	0	0	1000
BCL2	0	0	1000
IFT140	0	0	1000
CACNA2D2	0	0	1000
CCDC109B	0	0	1000
PDK1	0	0	1000
LINC00657	0	0	1000
TFRC	0	0	1000
LCK	0	0	1000
FAM26F	0	0	1000
CSAD	0	0	1000
NXNL2	0	0	1000
HLA-DOB	0	0	1000
MCM10	0	0	1000
APBB2	0	0	1000
TMEM138	0	0	1000
LRRC6	0	0	1000
MAL	0	0	1000
IGIP	0	0	1000
ZNF500	0	0	1000
AGR3	0	0	1000
LILRA6	0	0	1000
FAM179B	0	0	1000
SLC7A2	0	0	1000
SEMA3B	0	0	1000
CDC45	0	0	1000
C17orf97	0	0	1000
WDR19	0	0	1000
GFRA1	0	0	1000
BCL11A	0	0	1000
PTPRCAP	0	0	1000
CASC4	0	0	1000
SLFN11	0	0	1000
GATAD1	0	0	1000

KRT7	0	0	1000
HLA-DRA	0	0	1000
TES	0	0	1000
TNFRSF8	0	0	1000
IFNG	0	0	1000
STARD3	0	0	1000
SH2D2A	0	0	1000
FLNB	0	0	1000
RTF1	0	0	1000
S100A9	0	0	1000
WNT3	0	0	1000
BAIAP3	0	0	1000
NME3	0	0	1000
SLC35F2	0	0	1000
TTC8	0	0	1000
QDPR	0	0	1000
CD19	0	0	1000
PTPRT	0	0	1000
IDH2	0	0	1000
NUS1	0	0	1000
MMP1	0	0	1000
ANXA2R	0	0	1000
NUP50	0	0	1000
ATP11C	0	0	1000
NOSTRIN	0	0	1000
PPP1R1B	0	0	1000
KCNK15	0	0	1000
IL2RB	0	0	1000
LAMP3	0	0	1000
GIMAP5	0	0	1000
CDCA5	0	0	1000
ELOVL2	0	0	1000
SHISA4	0	0	1000
FAM47E	0	0	1000
ITPKA	0	0	1000
ABRACL	0	0	1000
CASP4	0	0	1000
ANXA9	0	0	1000
TNFAIP8	0	0	1000
MEIS3P1	0	0	1000
LPXN	0	0	1000
CHAC2	0	0	1000
TTLL4	0	0	1000
M28170	0	0	1000
PSME4	0	0	1000
CLEC4E	0	0	1000
AW149008	0	0	1000
LAMB2	0	0	1000
SLC7A7	0	0	1000
ZSCAN18	0	0	1000
PDE8B	0	0	1000
PNPLA4	0	0	1000
HLA-DPA1	0	0	1000
POTEI	0	0	1000

HCFC1R1	0	0	1000
FAM73A	0	0	1000
LYPD6B	0	0	1000
FAM198B	0	0	1000
SLC2A5	0	0	1000
MTL5	0	0	1000
NKG7	0	0	1000
DCTN4	0	0	1000
LILRB2	0	0	1000
PALM	0	0	1000
NUDT12	0	0	1000
CCDC104	0	0	1000
THAP10	0	0	1000
DECR2	0	0	1000
HEXIM2	0	0	1000
ABAT	0	0	1000
COQ7	0	0	1000
CENPA	0	0	1000
CDKN2AIPN	0	0	1000
IGFLR1	0	0	1000
KIAA0125	0	0	1000
EPSTI1	0	0	1000
FCER1G	0	0	1000
LOC100506	0	0	1000
FAN1	0	0	1000
TMC4	0	0	1000
TCTN1	0	0	1000
C16orf34	0	0	1000
NM_003197	0	0	1000
LRRC46	0	0	1000
C1orf226	0	0	1000
MAPK1	0	0	1000
EFS	0	0	1000
PHGDH	0	0	1000
RAB11FIP3	0	0	1000
ARHGEF17	0	0	1000
TIGIT	0	0	1000
ACOT9	0	0	1000
TCN2	0	0	1000
X72475	0	0	1000
KIAA0232	0	0	1000
LAPTM5	0	0	1000
CREBBP	0	0	1000
IVD	0	0	1000
MTHFD2	0	0	1000
TMEM25	0	0	1000
GPER1	0	0	1000
HSD17B2	0	0	1000
MYRIP	0	0	1000
EAF2	0	0	1000
MYB	0	0	1000
RAB17	0	0	1000
S100A8	0	0	1000
SERPINA11	0	0	1000

CCR2	0	0	1000
PDIA5	0	0	1000
ANO1	0	0	1000
MZB1	0	0	1000
PNOC	0	0	1000
IL10RA	0	0	1000
CCL5	0	0	1000
KIF5C	0	0	1000
RAD50	0	0	1000
CXCL11	0	0	1000
FADS2	0	0	1000
LIAS	0	0	1000
SCN4B	0	0	1000
SEL1L3	0	0	1000
PRKX	0	0	1000
TRIM27	0	0	1000
B4GALNT4	0	0	1000
SASH3	0	0	1000
DKK1	0	0	1000
SMCO4	0	0	1000
SLMAP	0	0	1000
CCDC24	0	0	1000
MYO5C	0	0	1000
PNP	0	0	1000
ZKSCAN2	0	0	1000
CCR5	0	0	1000
CATSPER2	0	0	1000
C1QA	0	0	1000
OSM	0	0	1000
FBXL5	0	0	1000
SLC1A2	0	0	1000
CROCCP2	0	0	1000
EPB41L1	0	0	1000
WDR24	0	0	1000
HSPB1	0	0	1000
ATG4D	0	0	1000
CD2	0	0	1000
CCDC64B	0	0	1000
SLA	0	0	1000
MGC16025	0	0	1000
VAMP5	0	0	1000
BID	0	0	1000
RAMP1	0	0	1000
SUSD3	0	0	1000
SIRPG	0	0	1000
MRPS30	0	0	1000
BTF3	0	0	1000
LYZ	0	0	1000
NMNAT3	0	0	1000
ERLIN2	0	0	1000
TNFRSF17	0	0	1000
DPY19L2P4	0	0	1000
TBX21	0	0	1000
UBE2J1	0	0	1000

C1QB	0	0	1000
BCL2A1	0	0	1000
CCL2	0	0	1000
IL4I1	0	0	1000
CPNE5	0	0	1000
CCDC103	0	0	1000
HCLS1	0	0	1000
IGDCC3	0	0	1000
CD5	0	0	1000
AI053442	0	0	1000
CASP5	0	0	1000
EPB41L5	0	0	1000
SIT1	0	0	1000
CHEK1	0	0	1000
C3orf52	0	0	1000
SEC61B	0	0	1000
SEPHS1	0	0	1000
C9orf19	0	0	1000
CD83	0	0	1000
CGN	0	0	1000
MYL5	0	0	1000
FAM134A	0	0	1000
HLA-DPB2	0	0	1000
CCR7	0	0	1000
TDO2	0	0	1000
ACOT2	0	0	1000
ZNF703	0	0	1000
BTF3P11	0	0	1000
ITM2C	0	0	1000
BRICD5	0	0	1000
PHYHD1	0	0	1000
FAM64A	0	0	1000
ARNTL2	0	0	1000
POMT2	0	0	1000
FSIP1	0	0	1000
CDKL3	0	0	1000
LAIR1	0	0	1000
SOCS1	0	0	1000
PLEK	0	0	1000
PTGDS	0	0	1000
TTC25	0	0	1000
NDUFA7	0	0	1000
SALL2	0	0	1000
PCSK6	0	0	1000
PLD6	0	0	1000
ZNF37A	0	0	1000
ERBB4	0	0	1000
JMJD8	0	0	1000
IL18BP	0	0	1000
GDPD3	0	0	1000
TCEA2	0	0	1000
CASC1	0	0	1000
PURA	0	0	1000
REPS2	0	0	1000

P2RY8	0	0	1000
CTTNBP2	0	0	1000
MED24	0	0	1000
SYTL3	0	0	1000
TNFRSF4	0	0	1000
NFIL3	0	0	1000
LMAN1	0	0	1000
TMEM76	0	0	1000
NDFIP1	0	0	1000
GMPS	0	0	1000
RFC4	0	0	1000
PCBP1-AS1	0	0	1000
HLA-DMA	0	0	1000
NAT2	0	0	1000
H2AFX	0	0	1000
FKBP10	0	0	1000
ZFYVE27	0	0	1000
ANKRD22	0	0	1000
MYCN	0	0	1000
IRF4	0	0	1000
LILRB3	0	0	1000
GLYATL2	0	0	1000
POTEG	0	0	1000
AW235069	0	0	1000
ZNF75A	0	0	1000
TRIM69	0	0	1000
KIF2C	0	0	1000
WDR83	0	0	1000
C5orf45	0	0	1000
TRMU	0	0	1000
KYNU	0	0	1000
SPG11	0	0	1000
METTL15	0	0	1000
DCDC5	0	0	1000
C20orf23	0	0	1000
FPR3	0	0	1000
GZMH	0	0	1000
CDC2L2	0	0	1000
TRIM45	0	0	1000
C16orf93	0	0	1000
CORO1A	0	0	1000
CPEB2	0	0	1000
CCL3	0	0	1000
PPFIBP2	0	0	1000
DNMBP	0	0	1000
MST1	0	0	1000
CD3G	0	0	1000
CHST15	0	0	1000
CIITA	0	0	1000
EGFL6	0	0	1000
PFKP	0	0	1000
EML4	0	0	1000
ZMAT2	0	0	1000
BUB1	0	0	1000

PTAFR	0	0	1000
ROGDI	0	0	1000
DCAF16	0	0	1000
CDON	0	0	1000
PFN1	0	0	1000
CCL17	0	0	1000
RBM11	0	0	1000
TEKT2	0	0	1000
DPY19L1P1	0	0	1000
CD3D	0	0	1000
KRT81	0	0	1000
IL2RA	0	0	1000
NUDT8	0	0	1000
HAUS8	0	0	1000
SPCS3	0	0	1000
CLIC4	0	0	1000
SLAMF1	0	0	1000
SLC31A1	0	0	1000
HEBP1	0	0	1000
HLA-H	0	0	1000
PSMB9	0	0	1000
CCL3L3	0	0	1000
TUBB	0	0	1000
OR7E24	0	0	1000
HLA-DQB1	0	0	1000
GSTP1	0	0	1000
CYB5R1	0	0	1000
CNNM4	0	0	1000
IDI1	0	0	1000
SKP2	0	0	1000
KIAA1407	0	0	1000
LAG3	0	0	1000
TXNDC16	0	0	1000
DACH1	0	0	1000
GPR171	0	0	1000
C11orf49	0	0	1000
CD8A	0	0	1000
PLEKHO1	0	0	1000
RASSF4	0	0	1000
C9orf116	0	0	1000
XCL2	0	0	1000
MAPKBP1	0	0	1000
NM_01633(	0	0	1000
SEMA3F	0	0	1000
PLEKHH3	0	0	1000
KLHL42	0	0	1000
DHRS1	0	0	1000
FPR1	0	0	1000
MEI1	0	0	1000
WDR60	0	0	1000
JMJD7	0	0	1000
PCP4L1	0	0	1000
C1orf162	0	0	1000
BATF3	0	0	1000

ORAOV1	0	0	1000
THRA	0	0	1000
C2orf81	0	0	1000
PLEKHA6	0	0	1000
HLA-DRB4	0	0	1000
ZNF14	0	0	1000
CLUAP1	0	0	1000
CYP2B6	0	0	1000
MPPED2	0	0	1000
ADAL	0	0	1000
IL32	0	0	1000
GIMAP6	0	0	1000
HLA-J	0	0	1000
CD6	0	0	1000
EEF1A2	0	0	1000
FMNL2	0	0	1000
AHNAK	0	0	1000
DOCK1	0	0	1000
KRT18P55	0	0	1000
VCAM1	0	0	1000
ACADS	0	0	1000
FAM173A	0	0	1000
HAGH	0	0	1000
HLA-C	0	0	1000
ELMO3	0	0	1000
LILRA4	0	0	1000
HLA-DQB2	0	0	1000
CD79A	0	0	1000
HLA-DRB3	0	0	1000
BATF2	0	0	1000
NUDT5	0	0	1000
E2F3	0	0	1000
EBI3	0	0	1000
FYB	0	0	1000
IGF2BP3	0	0	1000
AGBL2	0	0	1000
RAB11FIP1	0	0	1000
CTSS	0	0	1000
UFD1L	0	0	1000
ETV7	0	0	1000
DUSP6	0	0	1000
REEP2	0	0	1000
PADI2	0	0	1000
SPOCK2	0	0	1000
GLYR1	0	0	1000
KIF12	0	0	1000
SORD	0	0	1000
NRBF2	0	0	1000
GPR183	0	0	1000
SRPK1	0	0	1000
HLA-L	0	0	1000
PRKCQ-AS1	0	0	1000
PIM2	0	0	1000
KLHDC1	0	0	1000

HLA-A	0	0	1000
AMZ1	0	0	1000
GTF2I	0	0	1000
TMSB15B	0	0	1000
ZNF776	0	0	1000
REEP5	0	0	1000
KLHL6	0	0	1000
ZNF44	0	0	1000
SDC4	0	0	1000
CYSTM1	0	0	1000
EGFL8	0	0	1000
AGL	0	0	1000
S100A11	0	0	1000
SUOX	0	0	1000
MS4A2	0	0	1000
KIAA0141	0	0	1000
PDCD6IP	0	0	1000
GMNN	0	0	1000
TRAF3IP3	0	0	1000
FAM78A	0	0	1000
ACE2	0	0	1000
IRF1	0	0	1000
MORC4	0	0	1000
CYBB	0	0	1000
THEMIS2	0	0	1000
CELSR1	0	0	1000
KIF11	0	0	1000
EPHX2	0	0	1000
SLC35E3	0	0	1000
CTSL	0	0	1000
COTL1	0	0	1000
PLA2G7	0	0	1000
AMN1	0	0	1000
TTC39A	0	0	1000
JAK3	0	0	1000
PPP1R18	0	0	1000
MEAF6	0	0	1000
MS4A4A	0	0	1000
EID1	0	0	1000
FCGR3B	0	0	1000
DLGAP5	0	0	1000
MAP3K12	0	0	1000
CREB3L2	0	0	1000
YPEL3	0	0	1000
RHOBTB3	0	0	1000
FMN1	0	0	1000
GMFG	0	0	1000
VAV1	0	0	1000
TRAF3IP1	0	0	1000
RNF111	0	0	1000
GCNT1	0	0	1000
EARS2	0	0	1000
LOC44033(	0	0	1000
OSTF1	0	0	1000

ROM1	0	0	1000
TTC18	0	0	1000
DHRS4-AS1	0	0	1000
SRGN	0	0	1000
PSME2	0	0	1000
STMND1	0	0	1000
SCAMP1	0	0	1000
FDXR	0	0	1000
NDUFB10	0	0	1000
RCOR3	0	0	1000
AI655888	0	0	1000
FUT1	0	0	1000
HDAC11	0	0	1000
LCMT2	0	0	1000
KCNE4	0	0	1000
HIST1H1A	0	0	1000
PTPN7	0	0	1000
NCF2	0	0	1000
UCHL3	0	0	1000
TNFRSF21	0	0	1000
CLIC6	0	0	1000
M55914	0	0	1000
HSPB8	0	0	1000
GET4	0	0	1000
CD163	0	0	1000
AIF1L	0	0	1000
SLC25A5	0	0	1000
CHCHD5	0	0	1000
IL2RG	0	0	1000
CLCA2	0	0	1000
SERINC3	0	0	1000
TEX10	0	0	1000
KIAA1279	0	0	1000
GPR162	0	0	1000
POTED	0	0	1000
GSTA2	0	0	1000
ZSCAN29	0	0	1000
DBT	0	0	1000
GPR126	0	0	1000
FBXO38	0	0	1000
PROM1	0	0	1000
NBEA	0	0	1000
LANCL1	0	0	1000
NAT8L	0	0	1000
TIMM8A	0	0	1000
ASB13	0	0	1000
TRIM63	0	0	1000
CD274	0	0	1000
ARHGAP9	0	0	1000
JARID2	0	0	1000
TLR2	0	0	1000
NASP	0	0	1000
AHCYL2	0	0	1000
PCYOX1L	0	0	1000

GIMAP4	0	0	1000
NPHP1	0	0	1000
TRPV2	0	0	1000
ENTPD8	0	0	1000
CDKN2A	0	0	1000
ATP6AP1L	0	0	1000
C3AR1	0	0	1000
MEX3A	0	0	1000
PCM1	0	0	1000
MAGI2	0	0	1000
GCOM1	0	0	1000
RTN4IP1	0	0	1000
PEX19	0	0	1000
NFS1	0	0	1000
MS4A6A	0	0	1000
ADAM19	0	0	1000
MGST1	0	0	1000
BSN	0	0	1000
CD79B	0	0	1000
BIN2	0	0	1000
THBS4	0	0	1000
ZSCAN32	0	0	1000
RTN4RL1	0	0	1000
JMJD7-PLA	0	0	1000
FCN3	0	0	1000
RBM12	0	0	1000
CD247	0	0	1000
CEACAM1	0	0	1000
NABP1	0	0	1000
DCUN1D5	0	0	1000
BLM	0	0	1000
SKP1	0	0	1000
BTG3	0	0	1000
HLA-B	0	0	1000
SERPING1	0	0	1000
TAGAP	0	0	1000
MAGED2	0	0	1000
GPC2	0	0	1000
IFNGR1	0	0	1000
GLI3	0	0	1000
SRGAP1	0	0	1000
APOBEC3G	0	0	1000
DICER1-AS	0	0	1000
LILRB4	0	0	1000
DEK	0	0	1000
ZAP70	0	0	1000
ZNF205-AS	0	0	1000
CDC123	0	0	1000
FCGR3A	0	0	1000
CHMP4B	0	0	1000
PREX1	0	0	1000
CCNA2	0	0	1000
LAX1	0	0	1000
AMD1	0	0	1000

HLA-DOA	0	0	1000
MRPS27	0	0	1000
EPB41L4A-	0	0	1000
LZTFL1	0	0	1000
CCDC96	0	0	1000
IMPA2	0	0	1000
RNASE6	0	0	1000
NOVA1	0	0	1000
ZFP14	0	0	1000
GTPBP8	0	0	1000
IL1B	0	0	1000
LYN	0	0	1000
TSHZ1	0	0	1000
GMPR2	0	0	1000
FAM89B	0	0	1000
GATS	0	0	1000
CNOT8	0	0	1000
ECHDC2	0	0	1000
NANOS1	0	0	1000
AKR7A2	0	0	1000
ASB2	0	0	1000
CMKLR1	0	0	1000
MEMO1	0	0	1000
B2M	0	0	1000
SMIM11	0	0	1000
UTP15	0	0	1000
S1PR4	0	0	1000
TUBB6	0	0	1000
CD96	0	0	1000
ERBB3	0	0	1000
SNAPC2	0	0	1000
CYBA	0	0	1000
MZF1	0	0	1000
ABHD2	0	0	1000
KIF4A	0	0	1000
IL27RA	0	0	1000
IL7R	0	0	1000
MYOF	0	0	1000
MPI	0	0	1000
TRIM66	0	0	1000
SEMA3C	0	0	1000
HLA-F	0	0	1000
SYNPO2	0	0	1000
MYO15B	0	0	1000
FAAH	0	0	1000
DTX3	0	0	1000
NUBP2	0	0	1000
ZG16B	0	0	1000
XCL1	0	0	1000
ANKRD42	0	0	1000
ST6GAL1	0	0	1000
CCNL2	0	0	1000
COG5	0	0	1000
NCF4	0	0	1000

MCOLN2	0	0	1000
APOBEC3F	0	0	1000
EXO1	0	0	1000
GBP2	0	0	1000
FKBP11	0	0	1000
GCH1	0	0	1000
SOWAHD	0	0	1000
TRAF1	0	0	1000
HCST	0	0	1000
NCOR1	0	0	1000
CAMK2B	0	0	1000
SHROOM3	0	0	1000
ITFG3	0	0	1000
ZNF236	0	0	1000
TLR1	0	0	1000
ERO1L	0	0	1000
RTN2	0	0	1000
NME5	0	0	1000
MPHOSPH6	0	0	1000
CD163L1	0	0	1000
MUM1	0	0	1000
HMGCL	0	0	1000
IGSF6	0	0	1000
CYTH1	0	0	1000
ZNF609	0	0	1000
SULT1A1	0	0	1000
ADIRF	0	0	1000
HMGB3	0	0	1000
DNAJC16	0	0	1000
FAM219B	0	0	1000
ZNF516	0	0	1000
FAM98C	0	0	1000
IL18RAP	0	0	1000
POLR3K	0	0	1000
SPRYD3	0	0	1000
B3GNT5	0	0	1000
PAX9	0	0	1000
YWHAQ	0	0	1000
TMEM145	0	0	1000
CEBPB	0	0	1000
ASS1	0	0	1000
MYOZ3	0	0	1000
C5orf24	0	0	1000
ZFP2	0	0	1000
CARD6	0	0	1000
VPREB3	0	0	1000
FCRL2	0	0	1000
CRNDE	0	0	1000
LIMK2	0	0	1000
PREP	0	0	1000
LTB	0	0	1000
JSRP1	0	0	1000
UVSSA	0	0	1000
SERTAD3	0	0	1000

KMO	0	0	1000
RPP40	0	0	1000
SMC6	0	0	1000
PPP1R16A	0	0	1000
GPRIN2	0	0	1000
FASLG	0	0	1000
CIPC	0	0	1000
DONSON	0	0	1000
ZNF683	0	0	1000
METRN	0	0	1000
UPP1	0	0	1000
TNFAIP8L2	0	0	1000
MDM2	0	0	1000
MFAP1	0	0	1000
SLC15A3	0	0	1000
HLA-DRB5	0	0	1000
AGFG1	0	0	1000
KRT18	0	0	1000
HIP1	0	0	1000
USP7	0	0	1000
IGJ	0	0	1000
USP14	0	0	1000
CCDC151	0	0	1000
H2AFJ	0	0	1000
ZNF814	0	0	1000
NOL6	0	0	1000
POMGNT1	0	0	1000
ACACB	0	0	1000
SGK1	0	0	1000
CCDC106	0	0	1000
TIGD2	0	0	1000
NFIB	0	0	1000
PIGV	0	0	1000
MPZL2	0	0	1000
PLA2G12A	0	0	1000
KIAA0430	0	0	1000
LRRK1	0	0	1000
RIPK4	0	0	1000
MZT2B	0	0	1000
DOK2	0	0	1000
IL12RB2	0	0	1000
BRF2	0	0	1000
PHF20	0	0	1000
HPN	0	0	1000
S100A3	0	0	1000
SRI	0	0	1000
LRFN2	0	0	1000
BMPR1B	0	0	1000
NPL	0	0	1000
SULT1A4	0	0	1000
SELPLG	0	0	1000
PGM3	0	0	1000
RRP36	0	0	1000
EIF4B	0	0	1000

SIGIRR	0	0	1000
SPSB3	0	0	1000
FAM86FP	0	0	1000
TPGS1	0	0	1000
CCP110	0	0	1000
WNK4	0	0	1000
SLC9A8	0	0	1000
CCDC176	0	0	1000
IL6	0	0	1000
HOOK1	0	0	1000
VPS28	0	0	1000
LDHD	0	0	1000
PLAC8	0	0	1000
KAT2A	0	0	1000
MDP1	0	0	1000
FCRL5	0	0	1000
ULK2	0	0	1000
HCP5	0	0	1000
CHEK2	0	0	1000
SCPEP1	0	0	1000
SP140	0	0	1000
CHDH	0	0	1000
WDR35	0	0	1000
PTPMT1	0	0	1000
STMN1	0	0	1000
NCOA7	0	0	1000
AI005573	0	0	1000
CACNG1	0	0	1000
FLJ31306	0	0	1000
IQCK	0	0	1000
INPP5D	0	0	1000
MRPL10	0	0	1000
TNF	0	0	1000
VGLL1	0	0	1000
GHRL	0	0	1000
LAD1	0	0	1000
ADM	0	0	1000
RNF19A	0	0	1000
LOC72968[illegible]	0	0	1000
CEP55	0	0	1000
KIAA1324[illegible]	0	0	1000
PLAT	0	0	1000
TMEM106B	0	0	1000
DEXI	0	0	1000
HTRA4	0	0	1000
HLA-DPB1	0	0	1000
RAI2	0	0	1000
FAM171A1	0	0	1000
BMS1P20	0	0	1000
ITK	0	0	1000
MARCKS	0	0	1000
CDK1	0	0	1000
NCF1	0	0	1000
EVL	0	0	1000

TMEM121	0	0	1000
ANXA3	0	0	1000
P2RY14	0	0	1000
HMBS	0	0	1000
TANK	0	0	1000
RAB40C	0	0	1000
CD48	0	0	1000
S100A4	0	0	1000
AFF3	0	0	1000
UCN	0	0	1000
MMP16	0	0	1000
RHPN1	0	0	1000
HRK	0	0	1000
TRAF5	0	0	1000
C21orf91	0	0	1000
EPHB6	0	0	1000
IGFBP4	0	0	1000
PPFIA1	0	0	1000
UBE2B	0	0	1000
TMEM8B	0	0	1000
SPTBN5	0	0	1000
GRPR	0	0	1000
WDR90	0	0	1000
OR7E91P	0	0	1000
GZMA	0	0	1000
APBB3	0	0	1000
CD52	0	0	1000
GREB1L	0	0	1000
WNT4	0	0	1000
ATP5D	0	0	1000
NBPF11	0	0	1000
ZNF112	0	0	1000
ARSG	0	0	1000
C1orf106	0	0	1000
PTDSS2	0	0	1000
MLPH	0	0	1000
MTFR2	0	0	1000
CNIH3	0	0	1000
ZNF264	0	0	1000
PCBD2	0	0	1000
ZRANB1	0	0	1000
C12orf10	0	0	1000
KIF3B	0	0	1000
PLA2G10	0	0	1000
FAM13B	0	0	1000
SOAT1	0	0	1000
LYRM7	0	0	1000
RAC2	0	0	1000
MLKL	0	0	1000
SPN	0	0	1000
CDAN1	0	0	1000
GLS2	0	0	1000
DYSF	0	0	1000
RCAN1	0	0	1000

FERMT3	0	0	1000
RQCD1	0	0	1000
CTSH	0	0	1000
ALG1	0	0	1000
TCEA3	0	0	1000
RGL4	0	0	1000
APOL1	0	0	1000
XLOC_0019	0	0	1000
GNGT2	0	0	1000
TBC1D10C	0	0	1000
IRF8	0	0	1000
SRD5A1	0	0	1000
APOBEC3B	0	0	1000
CRELD2	0	0	1000
TRPS1	0	0	1000
GALNT3	0	0	1000
KDM3B	0	0	1000
SGK3	0	0	1000
TOX2	0	0	1000
COX19	0	0	1000
CARD10	0	0	1000
PTGR2	0	0	1000
NCKAP1L	0	0	1000
TTK	0	0	1000
CMYA5	0	0	1000
TOR4A	0	0	1000
DMXL1	0	0	1000
ZFAND5	0	0	1000
HSD11B1	0	0	1000
SEPT12	0	0	1000
HLA-DQA2	0	0	1000
NECAB3	0	0	1000
GRIN3A	0	0	1000
PSMD7	0	0	1000
LSM2	0	0	1000
ABCC5	0	0	1000
RAB33A	0	0	1000
HCAR3	0	0	1000
ATF4	0	0	1000
SYTL2	0	0	1000
SSR4	0	0	1000
TMEM181	0	0	1000
KIAA1191	0	0	1000
MARVELD2	0	0	1000
NPIPB5	0	0	1000
ASNS	0	0	1000
CTLA4	0	0	1000
STX11	0	0	1000
TAPT1	0	0	1000
ZBP1	0	0	1000
LOC101926	0	0	1000
OCLN	0	0	1000
SLC7A8	0	0	1000
CYB5D2	0	0	1000

PAK4	0	0	1000
CHN1	0	0	1000
CD70	0	0	1000
HSPA1A	0	0	1000
IFI16	0	0	1000
GSTA5	0	0	1000
FNDC3B	0	0	1000
FCGR2A	0	0	1000
PTGER4	0	0	1000
HHAT	0	0	1000
MGAT1	0	0	1000
TLE1	0	0	1000
MFSD2A	0	0	1000
HTR7P1	0	0	1000
CEP76	0	0	1000
NOS1AP	0	0	1000
GRTP1	0	0	1000
MCM7	0	0	1000
PLEKHO2	0	0	1000
ARL1	0	0	1000
UCK2	0	0	1000
CFLAR	0	0	1000
ABHD14B	0	0	1000
PHACTR2	0	0	1000
SLC36A4	0	0	1000
NUBPL	0	0	1000
PEX12	0	0	1000
SFXN2	0	0	1000
C1S	0	0	1000
ASCC2	0	0	1000
MAP3K5	0	0	1000
CASP1	0	0	1000
ST8SIA1	0	0	1000
GIN1	0	0	1000
ANO9	0	0	1000
CCL7	0	0	1000
ICAM2	0	0	1000
ARL4C	0	0	1000
KANSL1	0	0	1000
TRIM8	0	0	1000
CST9	0	0	1000
CPEB3	0	0	1000
PEX11B	0	0	1000
PPM1H	0	0	1000
VPS37B	0	0	1000
BBS5	0	0	1000
BEND3	0	0	1000
DSC2	0	0	1000
LRBA	0	0	1000
DCDC2	0	0	1000
LTC4S	0	0	1000
GLUL	0	0	1000
MRC1	0	0	1000
VCP	0	0	1000

CSRP2	0	0	1000
MYBL2	0	0	1000
CD300LF	0	0	1000
SLC35E2	0	0	1000
AP2B1	0	0	1000
SLX4	0	0	1000
HIST1H2AC	0	0	1000
PIGQ	0	0	1000
SREK1	0	0	1000
MRFAP1L1	0	0	1000
PAQR5	0	0	1000
TFF1	0	0	1000
STUB1	0	0	1000
SSH3	0	0	1000
PDE6B	0	0	1000
AOAH	0	0	1000
SNX20	0	0	1000
KARS	0	0	1000
TELO2	0	0	1000
DENND1C	0	0	1000
AP4E1	0	0	1000
PVRIG	0	0	1000
CEP290	0	0	1000
LRRC17	0	0	1000
RSL1D1	0	0	1000
HAGHL	0	0	1000
PLIN1	0	0	1000
PRKCB	0	0	1000
PGK1	0	0	1000
TRAK2	0	0	1000
SSBP2	0	0	1000
IFRD1	0	0	1000
PALM3	0	0	1000
GSDMB	0	0	1000
PSTPIP1	0	0	1000
MGAT4A	0	0	1000
GTSE1	0	0	1000
ZNF219	0	0	1000
MCM3	0	0	1000
C9orf89	0	0	1000
ZBTB16	0	0	1000
ZNF563	0	0	1000
KLHL12	0	0	1000
OGFOD2	0	0	1000
BTN3A3	0	0	1000
LILRB1	0	0	1000
LINC0047[illegible]	0	0	1000
C5orf15	0	0	1000
C17orf58	0	0	1000
AI694524	0	0	1000
TTC21A	0	0	1000
AIF1	0	0	1000
CDKN3	0	0	1000
MIR155HG	0	0	1000

PPP1R37	0	0	1000
FCGR2B	0	0	1000
PIGT	0	0	1000
MPEG1	0	0	1000
RGL1	0	0	1000
ARFGAP3	0	0	1000
KCNG1	0	0	1000
CYHR1	0	0	1000
CPLX1	0	0	1000
DESI2	0	0	1000
ITGB7	0	0	1000
LBR	0	0	1000
OR7E156P	0	0	1000
TMEM45A	0	0	1000
ANKEF1	0	0	1000
ZNF446	0	0	1000
RNF144B	0	0	1000
CD40	0	0	1000
CXCL17	0	0	1000
IL15RA	0	0	1000
MNDA	0	0	1000
LRP2	0	0	1000
RCN1	0	0	1000
CPVL	0	0	1000
JAKMIP1	0	0	1000
RRAGD	0	0	1000
INPP4A	0	0	1000
MXD4	0	0	1000
RBMS1	0	0	1000
ZCCHC10	0	0	1000
TRIM58	0	0	1000
PELI3	0	0	1000
PDCD5	0	0	1000
PPP1R3C	0	0	1000
TTC37	0	0	1000
GRIK3	0	0	1000
GAS5	0	0	1000
TMEM80	0	0	1000
SPDYE5	0	0	1000
SERPINA3	0	0	1000
AW183918	0	0	1000
UBE2L3	0	0	1000
PAFAH2	0	0	1000
ZNF721	0	0	1000
PLD1	0	0	1000
BANK1	0	0	1000
FANCA	0	0	1000
ARL5B	0	0	1000
AHCYL1	0	0	1000
OTUD7B	0	0	1000
DGAT1	0	0	1000
RAD1	0	0	1000
NAGK	0	0	1000
TRPC1	0	0	1000

SNX1	0	0	1000
KRTCAP3	0	0	1000
C20orf96	0	0	1000
SLC22A15	0	0	1000
C16orf80	0	0	1000
BBOX1	0	0	1000
MSL3	0	0	1000
AMIGO2	0	0	1000
RAB22A	0	0	1000
DCPS	0	0	1000
INADL	0	0	1000
FANK1	0	0	1000
C5	0	0	1000
APOBEC3C	0	0	1000
VNN2	0	0	1000
CLEC4A	0	0	1000
STK39	0	0	1000
COG2	0	0	1000
NCOR2	0	0	1000
GGCT	0	0	1000
CD74	0	0	1000
INSR	0	0	1000
DEFB1	0	0	1000
NOXA1	0	0	1000
RASEF	0	0	1000
PHF21A	0	0	1000
SIK3	0	0	1000
AKR7A3	0	0	1000
HOXC10	0	0	1000
LOC93622	0	0	1000
LDLRAD4	0	0	1000
ZNF552	0	0	1000
RGL3	0	0	1000
ARHGAP25	0	0	1000
MAPK8IP2	0	0	1000
RAB42	0	0	1000
GALNT16	0	0	1000
NISCH	0	0	1000
RBM24	0	0	1000
EML2	0	0	1000
PRDX4	0	0	1000
TNFRSF9	0	0	1000
FLJ42627	0	0	1000
KCTD3	0	0	1000
LOC10013[illegible]	0	0	1000
C16orf58	0	0	1000
ZBTB4	0	0	1000
TM4SF19	0	0	1000
C6orf141	0	0	1000
FAAH2	0	0	1000
IGF2	0	0	1000
CSF2RA	0	0	1000
NAMPT	0	0	1000
YTHDC2	0	0	1000

SMARCD3	0	0	1000
IFNAR2	0	0	1000
BRCA1	0	0	1000
ZNF214	0	0	1000
EVI2B	0	0	1000
FDCSP	0	0	1000
MAP9	0	0	1000
NCS1	0	0	1000
SPSB1	0	0	1000
GGT7	0	0	1000
PTPRK	0	0	1000
SLC22A4	0	0	1000
FBXO44	0	0	1000
PRF1	0	0	1000
MAP6	0	0	1000
PGRMC1	0	0	1000
CDKN1B	0	0	1000
IDUA	0	0	1000
CCDC85A	0	0	1000
RBM5	0	0	1000
DBI	0	0	1000
SAP18	0	0	1000
IGKV1D-8	0	0	1000
ZNF25	0	0	1000
AI810669	0	0	1000
GPC1	0	0	1000
IQCE	0	0	1000
GRIA2	0	0	1000
DENND1B	0	0	1000
KIAA1522	0	0	1000
CCDC25	0	0	1000
ERCC6L	0	0	1000
DYX1C1	0	0	1000
CDC20	0	0	1000
BSDC1	0	0	1000
AGAP2	0	0	1000
HENMT1	0	0	1000
AP1AR	0	0	1000
RAB9A	0	0	1000
ZNF580	0	0	1000
ATHL1	0	0	1000
PROSC	0	0	1000
PLCG2	0	0	1000
R3HDM2	0	0	1000
USP47	0	0	1000
WNT10A	0	0	1000
KLRD1	0	0	1000
SRRM2	0	0	1000
DMRTC1	0	0	1000
PHLDB3	0	0	1000
TMEM150C	0	0	1000
COASY	0	0	1000
BCAT2	0	0	1000
ARPC5L	0	0	1000

LAMA3	0	0	1000
FAM136A	0	0	1000
UGCG	0	0	1000
ARSB	0	0	1000
TRPV6	0	0	1000
NDC80	0	0	1000
YBX3	0	0	1000
FAM102B	0	0	1000
CEP57L1	0	0	1000
SYNCRIP	0	0	1000
ARL3	0	0	1000
AFF4	0	0	1000
TMEM176B	0	0	1000
FOLH1B	0	0	1000
GHDC	0	0	1000
NYX	0	0	1000
DDIT4	0	0	1000
CAMK2N1	0	0	1000
IKZF5	0	0	1000
FUZ	0	0	1000
BCL2L14	0	0	1000
GNB4	0	0	1000
BAIAP2-AS	0	0	1000
NFKBIE	0	0	1000
ACOT1	0	0	1000
GPR84	0	0	1000
POLR2D	0	0	1000
TEX264	0	0	1000
TPRG1	0	0	1000
PLCH1	0	0	1000
OSCP1	0	0	1000
APOA1BP	0	0	1000
TARS	0	0	1000
DPH3P1	0	0	1000
FAHD1	0	0	1000
TMEM55A	0	0	1000
CD68	0	0	1000
CD69	0	0	1000
MTMR9	0	0	1000
ZFAS1	0	0	1000
FOXA1	0	0	1000
KCNN3	0	0	1000
DOK3	0	0	1000
CKAP2L	0	0	1000
USB1	0	0	1000
ZFHX2	0	0	1000
AMER1	0	0	1000
CAP1	0	0	1000
ZNF557	0	0	1000
MTO1	0	0	1000
DNAJB11	0	0	1000
LSM6	0	0	1000
HN1	0	0	1000
MAOA	0	0	1000

TCEAL6	0	0	1000
SNX13	0	0	1000
C19orf33	0	0	1000
STXBP2	0	0	1000
MAN2A2	0	0	1000
SUV39H2	0	0	1000
RTN4R	0	0	1000
HJURP	0	0	1000
TMEM30B	0	0	1000
OPLAH	0	0	1000
MTA3	0	0	1000
TPP2	0	0	1000
PTGER3	0	0	1000
FNBP1	0	0	1000
ORMDL3	0	0	1000
ZNF587	0	0	1000
CD7	0	0	1000
HDAC5	0	0	1000
NEBL	0	0	1000
CD8B	0	0	1000
KITLG	0	0	1000
PLBD1	0	0	1000
LPIN1	0	0	1000
SP2	0	0	1000
YBX1	0	0	1000
TYROBP	0	0	1000
CLIP4	0	0	1000
SLC25A23	0	0	1000
SHC2	0	0	1000
SNRPG	0	0	1000
LCP2	0	0	1000
ACVR2B	0	0	1000
PDGFD	0	0	1000
MOAP1	0	0	1000
TMPRSS13	0	0	1000
CDT1	0	0	1000
WIPF1	0	0	1000
EMP3	0	0	1000
GLI1	0	0	1000
RNF208	0	0	1000
SPEF2	0	0	1000
NTHL1	0	0	1000
C15orf39	0	0	1000
FDPS	0	0	1000
DRG1	0	0	1000
ZADH2	0	0	1000
ARMC9	0	0	1000
SLC7A5	0	0	1000
ARHGAP30	0	0	1000
SMA4	0	0	1000
MIR100HG	0	0	1000
APPL2	0	0	1000
NUDT16L1	0	0	1000
TCEAL1	0	0	1000

PAK1IP1	0	0	1000
RGS1	0	0	1000
FBXO5	0	0	1000
GPRC5A	0	0	1000
PCMTD2	0	0	1000
HOMER3	0	0	1000
EFNA4	0	0	1000
UBE2C	0	0	1000
LOC15506(	0	0	1000
IL15	0	0	1000
PTPRA	0	0	1000
IK	0	0	1000
LRRC25	0	0	1000
POMC	0	0	1000
FAM183A	0	0	1000
DDOST	0	0	1000
PLS1	0	0	1000
AMICA1	0	0	1000
PJA2	0	0	1000
RFX2	0	0	1000
TRIM26	0	0	1000
EPS15L1	0	0	1000
MUCL1	0	0	1000
TSPAN1	0	0	1000
MYL12A	0	0	1000
FKBP4	0	0	1000
MCUR1	0	0	1000
SH3RF1	0	0	1000
ZNF271	0	0	1000
RUNDC1	0	0	1000
NLRC4	0	0	1000
PTCRA	0	0	1000
LYRM1	0	0	1000
ASB8	0	0	1000
PNMT	0	0	1000
ARID1A	0	0	1000
TYW5	0	0	1000
PDDC1	0	0	1000
DNAJC17	0	0	1000
LEAP2	0	0	1000
STK32B	0	0	1000
AKAP7	0	0	1000
PSMA4	0	0	1000
KLRC1	0	0	1000
C17orf96	0	0	1000
CP	0	0	1000
ACSL1	0	0	1000
DUSP22	0	0	1000
SNX24	0	0	1000
C12orf60	0	0	1000
PACS2	0	0	1000
HDAC2	0	0	1000
ARFGEF1	0	0	1000
E4F1	0	0	1000

RCSD1	0	0	1000
GIMAP7	0	0	1000
ATF3	0	0	1000
CD14	0	0	1000
SLC35F3	0	0	1000
MTHFD1L	0	0	1000
ZFP36	0	0	1000
MMP3	0	0	1000
RNF14	0	0	1000
TRPM7	0	0	1000
CTBP1-AS2	0	0	1000
WDFY3	0	0	1000
SPATA18	0	0	1000
MLST8	0	0	1000
GRK6	0	0	1000
KLF3-AS1	0	0	1000
C16orf45	0	0	1000
RBM47	0	0	1000
BTN3A2	0	0	1000
TPM4	0	0	1000
NPIPA1	0	0	1000
C2	0	0	1000
RIN1	0	0	1000
GTF3C6	0	0	1000
TENC1	0	0	1000
MORN4	0	0	1000
CSNK1G3	0	0	1000
PHC3	0	0	1000
MT1H	0	0	1000
AQP9	0	0	1000
CRYZ	0	0	1000
LRRC57	0	0	1000
PPP1R1A	0	0	1000
RUNX3	0	0	1000
KIAA0020	0	0	1000
ACTB	0	0	1000
C1orf27	0	0	1000
SMARCC2	0	0	1000
HMGN3	0	0	1000
SLPI	0	0	1000
DNAJC21	0	0	1000
UQCRFS1	0	0	1000
GALNT7	0	0	1000
C14orf79	0	0	1000
CD24	0	0	1000
6-Mar	0	0	1000
ENDOV	0	0	1000
NCK1	0	0	1000
MATN3	0	0	1000
LILRB5	0	0	1000
BBS2	0	0	1000
MYO6	0	0	1000
TP53BP1	0	0	1000
ZNF720	0	0	1000

FBXO15	0	0	1000
WASH2P	0	0	1000
PRRG2	0	0	1000
HPGDS	0	0	1000
FBXO41	0	0	1000
LAT	0	0	1000
COMT	0	0	1000
EIF3J	0	0	1000
MAGI1	0	0	1000
BARX1	0	0	1000
HLA-G	0	0	1000
C2orf15	0	0	1000
RHBDF1	0	0	1000
NT5C2	0	0	1000
GPATCH8	0	0	1000
ALDH7A1	0	0	1000
NBPF9	0	0	1000
ODC1	0	0	1000
HIST1H2AK	0	0	1000
C5orf20	0	0	1000
PPP1R16B	0	0	1000
CDCA8	0	0	1000
RAE1	0	0	1000
PIM1	0	0	1000
NIPA1	0	0	1000
DISP1	0	0	1000
RASAL3	0	0	1000
FLJ32255	0	0	1000
LINC00950	0	0	1000
HERC2	0	0	1000
NEURL2	0	0	1000
CXorf65	0	0	1000
SLC41A2	0	0	1000
FDPSP2	0	0	1000
MFNG	0	0	1000
TIMM13	0	0	1000
GAS8	0	0	1000
NT5M	0	0	1000
LINC01116	0	0	1000
NPDC1	0	0	1000
RPS7	0	0	1000
DYNLRB2	0	0	1000
TRAPPC6A	0	0	1000
MICU3	0	0	1000
TRIM4	0	0	1000
FCN1	0	0	1000
C1orf167	0	0	1000
SETBP1	0	0	1000
DEPDC1	0	0	1000
CCDC117	0	0	1000
MMS22L	0	0	1000
CA9	0	0	1000
LINC00883	0	0	1000
FAM63B	0	0	1000

SERPINB8	0	0	1000
ZNF91	0	0	1000
ARL9	0	0	1000
MPC2	0	0	1000
RSU1	0	0	1000
TMEM101	0	0	1000
PRKY	0	0	1000
FEN1	0	0	1000
PIP5K1B	0	0	1000
KRT37	0	0	1000
RALGAPB	0	0	1000
PIANP	0	0	1000
ZNF862	0	0	1000
CALML5	0	0	1000
ATG16L2	0	0	1000
ACLY	0	0	1000
SATB2	0	0	1000
TCEAL4	0	0	1000
ABHD1	0	0	1000
TMEM9B	0	0	1000
LIME1	0	0	1000
LRMP	0	0	1000
SPRED1	0	0	1000
TEKT4	0	0	1000
ZMYND12	0	0	1000
LRRC48	0	0	1000
TTBK2	0	0	1000
ID2	0	0	1000
TBC1D19	0	0	1000
RARRES2	0	0	1000
USP8	0	0	1000
GRHL1	0	0	1000
IFT88	0	0	1000
NCBP1	0	0	1000
KIAA1432	0	0	1000
DERL2	0	0	1000
DNMT3B	0	0	1000
KLHDC10	0	0	1000
FLJ30064	0	0	1000
HLA-DMB	0	0	1000
RPN1	0	0	1000
G26403	0	0	1000
BTK	0	0	1000
UBE2D1	0	0	1000
TMEM67	0	0	1000
NBR1	0	0	1000
TLE3	0	0	1000
BHLHE40	0	0	1000
ASGR1	0	0	1000
TPPP3	0	0	1000
PNMA1	0	0	1000
FBXO36	0	0	1000
SLC35A3	0	0	1000
ARPC5	0	0	1000

KIF16B	0	0	1000
FDFT1	0	0	1000
ERGIC1	0	0	1000
CDK6	0	0	1000
HSD17B8	0	0	1000
LYSMD4	0	0	1000
PHEX	0	0	1000
C16orf13	0	0	1000
SYT1	0	0	1000
STARD4	0	0	1000
AHSA2	0	0	1000
KLRC4	0	0	1000
PKP4	0	0	1000
ZNF304	0	0	1000
SIGLEC17P	0	0	1000
RNASEH1	0	0	1000
DGKQ	0	0	1000
GHR	0	0	1000
RNF125	0	0	1000
RHOJ	0	0	1000
NEK9	0	0	1000
SEMA3G	0	0	1000
KIF14	0	0	1000
THEM4	0	0	1000
PPIL6	0	0	1000
SPATA5L1	0	0	1000
DNAJA3	0	0	1000
SPNS3	0	0	1000
ZC4H2	0	0	1000
CYB5R4	0	0	1000
CCDC11	0	0	1000
AKAP11	0	0	1000
TFEC	0	0	1000
ADCY7	0	0	1000
NPEPPS	0	0	1000
MUSTN1	0	0	1000
SLC29A1	0	0	1000
MYCBPAP	0	0	1000
DDC	0	0	1000
GUSBP3	0	0	1000
ZBTB32	0	0	1000
EPB41L4B	0	0	1000
CDKAL1	0	0	1000
USP1	0	0	1000
NEK10	0	0	1000
SMR3A	0	0	1000
CEP104	0	0	1000
FAM122A	0	0	1000
KIAA1109	0	0	1000
SPARCL1	0	0	1000
KIF2A	0	0	1000
PARP8	0	0	1000
CAPS2	0	0	1000
LOC101927	0	0	1000

TCEB2	0	0	1000
GLOD5	0	0	1000
ZFYVE28	0	0	1000
SREBF2	0	0	1000
OR8B8	0	0	1000
ZDHHC20	0	0	1000
MYO10	0	0	1000
ZNF174	0	0	1000
AA148535	0	0	1000
IL3RA	0	0	1000
RANBP1	0	0	1000
CHML	0	0	1000
RFX7	0	0	1000
SBF1	0	0	1000
PPIP5K2	0	0	1000
SCLY	0	0	1000
BAG1	0	0	1000
SLC24A1	0	0	1000
ADORA1	0	0	1000
UBE2A	0	0	1000
HRASLS	0	0	1000
UGT2B7	0	0	1000
DZANK1	0	0	1000
FAM193A	0	0	1000
SLC23A2	0	0	1000
SNHG11	0	0	1000
HEYL	0	0	1000
MAATS1	0	0	1000
PTTG2	0	0	1000
SUDS3	0	0	1000
CNIH4	0	0	1000
LOC101927	0	0	1000
CFL1	0	0	1000
CST9L	0	0	1000
FIGN	0	0	1000
ZNRF3	0	0	1000
PDE5A	0	0	1000
UBE2E3	0	0	1000
P2RX4	0	0	1000
H2BFXP	0	0	1000
AGAP9	0	0	1000
OCIAD1	0	0	1000
ENPP5	0	0	1000
GPR19	0	0	1000
HTT	0	0	1000
TNRC6B	0	0	1000
L3MBTL1	0	0	1000
RELT	0	0	1000
LRIG3	0	0	1000
CD97	0	0	1000
TNIP3	0	0	1000
LIMS1	0	0	1000
ARHGEF40	0	0	1000
EDN2	0	0	1000

PPP2R5C	0	0	1000
RAB27A	0	0	1000
UQCRH	0	0	1000
MPP2	0	0	1000
REM2	0	0	1000
GIMAP1	0	0	1000
NABP2	0	0	1000
CEP68	0	0	1000
FMO1	0	0	1000
STT3A	0	0	1000
RIPK2	0	0	1000
NLGN2	0	0	1000
PANX1	0	0	1000
WNT2	0	0	1000
NPIPB9	0	0	1000
CHRD	0	0	1000
KRT8	0	0	1000
RNLS	0	0	1000
ARID2	0	0	1000
TMEM87A	0	0	1000
PSMB2	0	0	1000
LRRC8C	0	0	1000
ZBTB48	0	0	1000
BCL2L12	0	0	1000
FAM102A	0	0	1000
CALR	0	0	1000
SGSM2	0	0	1000
SULT1A2	0	0	1000
GPHN	0	0	1000
FAM120A	0	0	1000
ZW10	0	0	1000
CACNA1H	0	0	1000
EIF3C	0	0	1000
CHAC1	0	0	1000
TTYH3	0	0	1000
FAM208A	0	0	1000
C10orf82	0	0	1000
CYTH4	0	0	1000
FCGRT	0	0	1000
CRY2	0	0	1000
SH2D1A	0	0	1000
APEH	0	0	1000
WFIKKN1	0	0	1000
SESTD1	0	0	1000
HVCN1	0	0	1000
STX18	0	0	1000
RASGRP3	0	0	1000
NRN1L	0	0	1000
TNFSF10	0	0	1000
POLR3E	0	0	1000
TNK1	0	0	1000
SLC38A5	0	0	1000
CBLN2	0	0	1000
ZNF688	0	0	1000

FRS2	0	0	1000
C10orf32	0	0	1000
KIAA1377	0	0	1000
PBX1	0	0	1000
ZNF577	0	0	1000
ARF3	0	0	1000
C1orf198	0	0	1000
MFSD3	0	0	1000
GDE1	0	0	1000
RFTN1	0	0	1000
OSBPL3	0	0	1000
SMAD3	0	0	1000
SHPK	0	0	1000
FAM183B	0	0	1000
BAZ2A	0	0	1000
TNFSF14	0	0	1000
MAT2A	0	0	1000
CD1A	0	0	1000
NLRC3	0	0	1000
ZNF606	0	0	1000
FOXD2	0	0	1000
RGS5	0	0	1000
CC2D2A	0	0	1000
BIRC3	0	0	1000
PPP2R3C	0	0	1000
LURAP1L	0	0	1000
GABPB1-AS	0	0	1000
GZMK	0	0	1000
DHTKD1	0	0	1000
CCDC61	0	0	1000
SCRIB	0	0	1000
SLC30A8	0	0	1000
FAM25A	0	0	1000
EPHA10	0	0	1000
BUD13	0	0	1000
TLR6	0	0	1000
PLAUR	0	0	1000
KANSL2	0	0	1000
NLRP7	0	0	1000
XPNPEP1	0	0	1000
GPRASP1	0	0	1000
THSD7A	0	0	1000
CTSW	0	0	1000
VAV3	0	0	1000
ING1	0	0	1000
CNNM3	0	0	1000
CHD3	0	0	1000
CD80	0	0	1000
APOL6	0	0	1000
RPL22L1	0	0	1000
KATNAL2	0	0	1000
TSC2	0	0	1000
PABPC1L	0	0	1000
PTPN22	0	0	1000

TIGD7	0	0	1000
TNNT1	0	0	1000
RASSF7	0	0	1000
FAM83D	0	0	1000
MEIS3	0	0	1000
CDS1	0	0	1000
GPR89B	0	0	1000
PER1	0	0	1000
C19orf38	0	0	1000
TRIM33	0	0	1000
EMR2	0	0	1000
FBP1	0	0	1000
PTPLAD1	0	0	1000
STEAP1	0	0	1000
CTR9	0	0	1000
TPTE2P5	0	0	1000
SPANXD	0	0	1000
BAP1	0	0	1000
CUX1	0	0	1000
SLC35C1	0	0	1000
CHMP2A	0	0	1000
BLK	0	0	1000
FAM20A	0	0	1000
HECA	0	0	1000
AMT	0	0	1000
SEZ6L2	0	0	1000
MAPK8IP3	0	0	1000
NDUFA2	0	0	1000
BDP1	0	0	1000
RPL23AP32	0	0	1000
TUSC2	0	0	1000
MAGIX	0	0	1000
PREPL	0	0	1000
GFER	0	0	1000
TUBA1C	0	0	1000
NMI	0	0	1000
RASGRP2	0	0	1000
CCDC136	0	0	1000
FBXO2	0	0	1000
HOMEZ	0	0	1000
PTPRC	0	0	1000
NAB1	0	0	1000
LARS	0	0	1000
SCARB1	0	0	1000
SULT2B1	0	0	1000
GDAP1	0	0	1000
SDC1	0	0	1000
TCF12	0	0	1000
PAN2	0	0	1000
SHRM	0	0	1000
ZC3H7A	0	0	1000
STXBP3	0	0	1000
TSNAX	0	0	1000
IL17D	0	0	1000

SLU7	0	0	1000
PPIB	0	0	1000
ANKMY1	0	0	1000

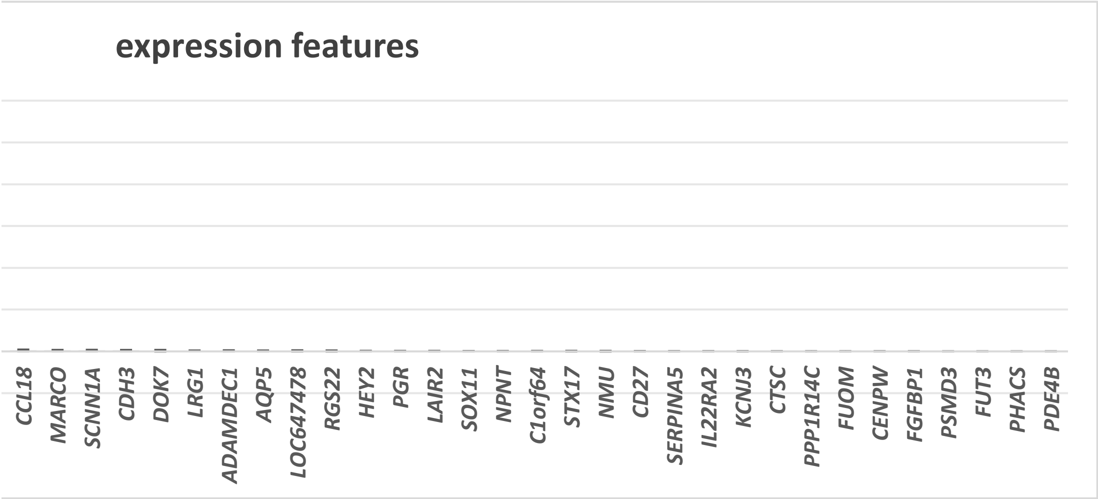
expression features
CCL18
MARCO
SCNN1A
CDH3
DOK7
LRG1
ADAMDEC1
AQP5
LOC647478
RGS22
HEY2
PGR
LAIR2
SOX11
NPNT
C1orf64
STX17
NMU
CD27
SERPINA5
IL22RA2
KCNJ3
CTSC
PPP1R14C
FUOM
CENPW
FGFBP1
PSMD3
FUT3
PHACS
PDE4B

Feature	Mean	Std	
Cyclin.D1	0.0788416	0.0160716	1000
STAT5.Y69	0.0507833	0.00928703	1000
ATR.S428	0.0464512	0.0105594	1000
IRS1.S612	0.0411527	0.0117562	1000
Estrogen.	0.0387532	0.00970821	1000
p70S6K.T4	0.0282811	0.00575405	1000
STAT1.Y70	0.0275472	0.00427835	1000
AMPKb1.S1	0.0269324	0.00776387	1000
ALK.Y1586	0.0247404	0.003252	1000
MET.Y1234	0.0196436	0.00625345	1000
ALK.Y1604	0.0169118	0.00284925	1000
CHK2.S33.	0.0152826	0.00284642	1000
AKT.T308	0.0150925	0.00749179	1000
p90RSK.S3	0.0118208	0.00408123	1000
ERBB3.Y12	0.0109562	0.0054357	1000
ERBB2.Y12	0.0104875	0.00760912	1000
STAT3.Y70	0.0103415	0.00524837	1000
AKT.S473	0.00957303	0.00505959	1000
Caspase.7	0.00869507	0.00508179	1000
PI3K.p85.	0.00801498	0.00426483	1000
IkBa.S32.	0.00796311	0.00336606	1000
p53.S15	0.00674703	0.00332285	1000
A.RAF.S29	0.00667403	0.00117245	1000
FAK.Y576.	0.00637433	0.00446307	1000
RB.S780	0.00637049	0.00479148	1000
CREB.S133	0.0061899	0.00133037	1000
BAD.S136	0.00613227	0.00276936	1000
eNOS.S113	0.0059805	0.00207315	1000
FADD.S194	0.00528889	0.00294637	1000
EGFR.tota	0.00524086	0.00237473	1000
Acetyl.Co	0.00491235	0.00263536	1000
ERBB3.tot	0.00489314	0.00259677	1000
AMPKa1.S4	0.00453388	0.00296414	1000
eIF4G.S11	0.00406128	0.00238686	1000
PTEN.tota	0.00386341	0.00196131	1000
mTOR.S244	0.00371932	0.00210861	1000
JAK1.Y102	0.00334086	0.00147414	1000
CHK1.S345	0.00328707	0.00183042	1000
p38.MAPK.	0.00297584	0.00177572	1000
Cofilin.S	0.00292973	0.00134465	1000
p27.T187	0.00264541	0.0021781	1000
PTEN.S380	0.00261275	0.00229452	1000
ERK1.2.T2	0.00173479	0.0011946	1000
IGF1R.Y11	0.0015811	0.00167323	1000
Cyclin.B1	0.00116613	0.000837219	1000
LC3B.tota	0.00111234	0.00200279	1000
S6RP.S240	0.00110465	0.00107116	1000
X4EBP1.S6	0.00108928	0.00132462	1000
Cyclin.A2	0.000960569	0.000241483	1000
E.cadheri	0.000628212	0.000245169	1000
MEK1.2.S2	0.000535997	0.00101084	1000
ATM.S1981	0.000501417	0.00162108	1000
SRC.Y527	0.000403439	0.000823919	1000

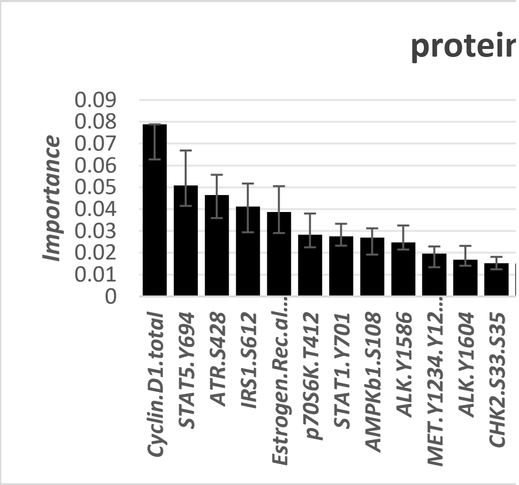

PLK1.T21(	0.000399597	0.000698819	1000
p70S6K.S3	0.000389991	0.0021173	1000
EGFR.Y114	0.00033812	0.000439575	1000
ERBB2.tot	0.000316988	0.00128234	1000
ERBB4.tot	0.000305461	0.00169298	1000
Ki67.tota	0.000301619	0.000619536	1000
STAT3.S72	0.000267038	0.000124415	1000
FOXO3a.S2	0.000242063	0.000653847	1000
EGFR.Y117	0.000232458	0.000407009	1000
VEGFR2.Y9	0.000207483	0.000508452	1000
GSK3aB.S2	0.000195956	0.000222272	1000
Caspase.9	0.000132558	0.000246804	1000
PARP..cle	0.000126795	0.000119111	1000
cKIT.Y703	0.000107584	0.000373828	1000
B.RAF.S44	9.41E-05	0.00050535	1000
Aurora.A.	1.92E-05	0.000663585	1000
EGFR.Y106	0	0	1000
cABL.T735	0	0	1000
FOXO1.S25	0	0	1000
FOXO1.T24	0	0	1000
Heregulir	0	0	1000
p70S6K.T3	0	0	1000
RTK.ROR1.	0	0	1000
SHC.Y317	0	0	1000
YAP.S127	0	0	1000
eNOS.NOSI	-1.92E-05	0.000923086	1000
PDGFRb.Y7	-0.000434177	0.000430969	1000
ERBB2.Y87	-0.000718505	0.00105089	1000
eIF4E.S2(	-0.000724269	0.000828044	1000

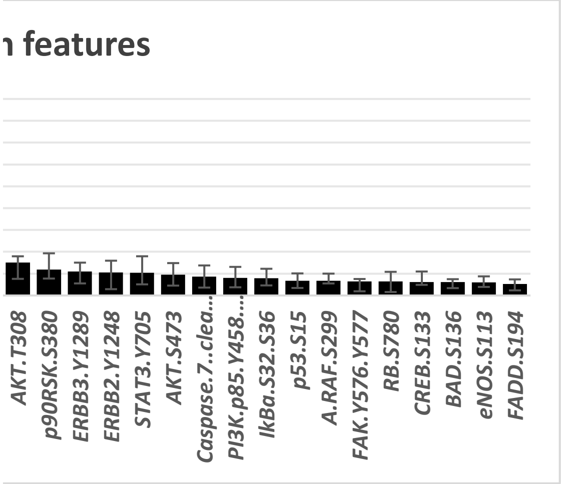
n features
AKT.T308
p90RSK.S380
ERBB3.Y1289
ERBB2.Y1248
STAT3.Y705
AKT.S473
Caspase.7..clea...
PI3K.p85.Y458....
IkBa.S32.S36
p53.S15
A.RAF.S299
FAK.Y576.Y577
RB.S780
CREB.S133
BAD.S136
eNOS.S113
FADD.S194

Feature	Mean	Std	
original_	0.116596	0.00727115	1000
wavelet-I	0.0714663	0.00497338	1000
original_	0.0452024	0.00885499	1000
log-sigma	0.0343557	0.0103065	1000
log-sigma	0.0292416	0.00745022	1000
wavelet-I	0.0258105	0.00258504	1000
wavelet-I	0.0257163	0.00831098	1000
wavelet-I	0.0255819	0.00956367	1000
wavelet-I	0.0249844	0.00254419	1000
wavelet-I	0.0231535	0.00546901	1000
wavelet-I	0.0201969	0.00448406	1000
wavelet-I	0.0190346	0.00304832	1000
wavelet-I	0.017734	0.00568809	1000
log-sigma	0.0159647	0.00361421	1000
wavelet-I	0.0154671	0.00457448	1000
diagnosti	0.0137784	0.00420822	1000
wavelet-I	0.0134038	0.00453914	1000
wavelet-I	0.0123491	0.00338236	1000
wavelet-I	0.0104087	0.00126453	1000
wavelet-I	0.00989001	0.00193624	1000
wavelet-I	0.00962106	0.00453316	1000
log-sigma	0.00913309	0.00255329	1000
wavelet-I	0.00865088	0.00152766	1000
wavelet-I	0.00818981	0.00210001	1000
wavelet-I	0.00756544	0.00157595	1000
wavelet-I	0.00685078	0.00247515	1000
wavelet-I	0.0064262	0.00280662	1000
log-sigma	0.00616301	0.00137243	1000
wavelet-I	0.00597858	0.00136745	1000
wavelet-I	0.0058806	0.00245211	1000
wavelet-I	0.00554632	0.00232337	1000
wavelet-I	0.00554248	0.00120469	1000
wavelet-I	0.00547908	0.00261587	1000
wavelet-I	0.00533692	0.000748864	1000
wavelet-I	0.00515441	0.00330654	1000
wavelet-I	0.00433601	0.00106002	1000
wavelet-I	0.00396715	0.00405202	1000
wavelet-I	0.00388262	0.00109507	1000
wavelet-I	0.00309879	0.000930069	1000
wavelet-I	0.00300466	0.000841217	1000
wavelet-I	0.00297008	0.00153907	1000
wavelet-I	0.00283944	0.00148846	1000
wavelet-I	0.00280486	0.00130544	1000
wavelet-I	0.00274531	0.00153126	1000
wavelet-I	0.0026089	0.00149121	1000
wavelet-I	0.00243216	0.000720706	1000
wavelet-I	0.00241295	0.000520249	1000
wavelet-I	0.00224581	0.00144713	1000
wavelet-I	0.00206906	0.000813882	1000
wavelet-I	0.00199222	0.000583678	1000
wavelet-I	0.00190961	0.00112207	1000
wavelet-I	0.0018174	0.000821191	1000
wavelet-I	0.00169829	0.000637297	1000

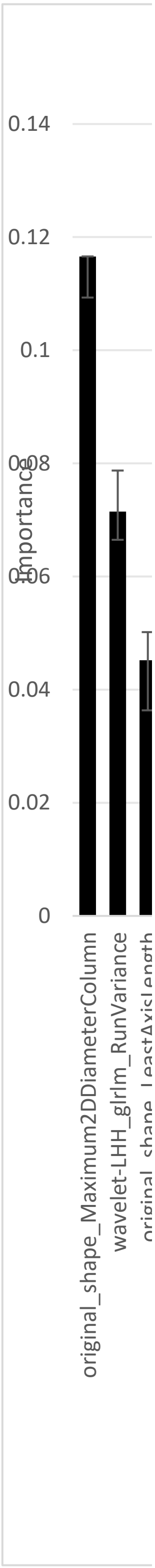

diagnosti	0.00148888	0.000631029	1000
log-sigma	0.00143317	0.0010363	1000
wavelet-H	0.00132558	0.00208631	1000
wavelet-H	0.00123337	0.00105547	1000
wavelet-H	0.00121416	0.00155872	1000
wavelet-L	0.00119495	0.00172649	1000
original_	0.00115076	0.00151539	1000
wavelet-H	0.00101628	0.00112886	1000
original_	0.000981701	0.0009642	1000
original_	0.000964411	0.00130296	1000
log-sigma	0.000943278	0.000830643	1000
wavelet-L	0.000829931	0.000661653	1000
wavelet-H	0.000826089	0.000887952	1000
wavelet-L	0.000814562	0.000798634	1000
wavelet-L	0.000799193	0.000735758	1000
wavelet-L	0.000799193	0.00144922	1000
original_	0.000774218	0.000720532	1000
wavelet-H	0.000768455	0.00124713	1000
log-sigma	0.000724269	0.000243128	1000
log-sigma	0.000680083	0.000202076	1000
wavelet-H	0.000666635	0.000746841	1000
wavelet-L	0.000582105	0.000271323	1000
wavelet-L	0.000562893	0.00035008	1000
wavelet-L	0.000480284	0.000482393	1000
log-sigma	0.00045531	0.000110795	1000
wavelet-H	0.000441862	0.000512083	1000
wavelet-L	0.000401518	0.000688652	1000
wavelet-H	0.000399597	0.000628174	1000
wavelet-H	0.000380385	0.000837241	1000
wavelet-L	0.000372701	0.000712672	1000
original_	0.000315067	0.00168993	1000
wavelet-H	0.00025359	0.000533945	1000
wavelet-L	0.000243984	7.28E-05	1000
wavelet-L	0.000217089	0.000451798	1000
wavelet-H	0.000192114	0.000207714	1000
wavelet-H	0.000136401	0.000108574	1000
log-sigma	6.53E-05	9.52E-05	1000
log-sigma	6.34E-05	0.000106376	1000
wavelet-H	5.96E-05	7.81E-05	1000
wavelet-L	4.42E-05	3.36E-05	1000
wavelet-H	1.54E-05	4.24E-05	1000
original_	5.76E-06	0.000392383	1000
log-sigma	5.76E-06	7.68E-06	1000
diagnosti	0	0	1000
diagnosti	0	0	1000
diagnosti	0	0	1000
diagnosti	0	0	1000
diagnosti	0	0	1000
diagnosti	0	0	1000
original_	0	0	1000
diagnosti	0	0	1000
diagnosti	0	0	1000
diagnosti	0	0	1000
original_	0	0	1000

diagnosti	0	0	1000
original_	0	0	1000
original_	0	0	1000
original_	0	0	1000
original_	0	0	1000
original_	0	0	1000
original_	0	0	1000
original_	0	0	1000
original_	0	0	1000
original_	0	0	1000
original_	0	0	1000
original_	0	0	1000
original_	0	0	1000
original_	0	0	1000
original_	0	0	1000
original_	0	0	1000
original_	0	0	1000
original_	0	0	1000
original_	0	0	1000
original_	0	0	1000
original_	0	0	1000
original_	0	0	1000
original_	0	0	1000
original_	0	0	1000
original_	0	0	1000
original_	0	0	1000
original_	0	0	1000
original_	0	0	1000
original_	0	0	1000
original_	0	0	1000
original_	0	0	1000
original_	0	0	1000
original_	0	0	1000
original_	0	0	1000
original_	0	0	1000
original_	0	0	1000
original_	0	0	1000
original_	0	0	1000
original_	0	0	1000
original_	0	0	1000
original_	0	0	1000
original_	0	0	1000
original_	0	0	1000
original_	0	0	1000
original_	0	0	1000
original_	0	0	1000
original_	0	0	1000
original_	0	0	1000
original_	0	0	1000
original_	0	0	1000
original_	0	0	1000
original_	0	0	1000
original_	0	0	1000
original_	0	0	1000

original_	0	0	1000
original_	0	0	1000
original_	0	0	1000
original_	0	0	1000
original_	0	0	1000
original_	0	0	1000
original_	0	0	1000
original_	0	0	1000
original_	0	0	1000
original_	0	0	1000
original_	0	0	1000
original_	0	0	1000
original_	0	0	1000
original_	0	0	1000
original_	0	0	1000
original_	0	0	1000
original_	0	0	1000
original_	0	0	1000
original_	0	0	1000
original_	0	0	1000
original_	0	0	1000
original_	0	0	1000
original_	0	0	1000
original_	0	0	1000
original_	0	0	1000
original_	0	0	1000
original_	0	0	1000
original_	0	0	1000
original_	0	0	1000
original_	0	0	1000
original_	0	0	1000
original_	0	0	1000
original_	0	0	1000
original_	0	0	1000
original_	0	0	1000
original_	0	0	1000
original_	0	0	1000
original_	0	0	1000
original_	0	0	1000
original_	0	0	1000
original_	0	0	1000
original_	0	0	1000
original_	0	0	1000
wavelet-l	0	0	1000
wavelet-l	0	0	1000
wavelet-l	0	0	1000
wavelet-l	0	0	1000
wavelet-l	0	0	1000
wavelet-l	0	0	1000
wavelet-l	0	0	1000
wavelet-l	0	0	1000
wavelet-l	0	0	1000
wavelet-l	0	0	1000
wavelet-l	0	0	1000

wavelet-I	0	0	1000
wavelet-I	0	0	1000
wavelet-I	0	0	1000
wavelet-I	0	0	1000
wavelet-I	0	0	1000
wavelet-I	0	0	1000
wavelet-I	0	0	1000
wavelet-I	0	0	1000
wavelet-I	0	0	1000
wavelet-I	0	0	1000
wavelet-I	0	0	1000
wavelet-I	0	0	1000
wavelet-I	0	0	1000
wavelet-I	0	0	1000
wavelet-I	0	0	1000
wavelet-I	0	0	1000
wavelet-I	0	0	1000
wavelet-I	0	0	1000
wavelet-I	0	0	1000
wavelet-I	0	0	1000
wavelet-I	0	0	1000
wavelet-I	0	0	1000
wavelet-I	0	0	1000
wavelet-I	0	0	1000
wavelet-I	0	0	1000
wavelet-I	0	0	1000
wavelet-I	0	0	1000
wavelet-I	0	0	1000
wavelet-I	0	0	1000
wavelet-I	0	0	1000
wavelet-I	0	0	1000
wavelet-I	0	0	1000
wavelet-I	0	0	1000
wavelet-I	0	0	1000
wavelet-I	0	0	1000
wavelet-I	0	0	1000
wavelet-I	0	0	1000
wavelet-I	0	0	1000
wavelet-I	0	0	1000
wavelet-I	0	0	1000
wavelet-I	0	0	1000
wavelet-I	0	0	1000
wavelet-I	0	0	1000
wavelet-I	0	0	1000
wavelet-I	0	0	1000
wavelet-I	0	0	1000
wavelet-I	0	0	1000
wavelet-I	0	0	1000
wavelet-I	0	0	1000
wavelet-I	0	0	1000
wavelet-I	0	0	1000
wavelet-I	0	0	1000
wavelet-I	0	0	1000
wavelet-I	0	0	1000

wavelet-I	0	0	1000
wavelet-I	0	0	1000
wavelet-I	0	0	1000
wavelet-I	0	0	1000
wavelet-I	0	0	1000
wavelet-I	0	0	1000
wavelet-I	0	0	1000
wavelet-I	0	0	1000
wavelet-I	0	0	1000
wavelet-I	0	0	1000
wavelet-I	0	0	1000
wavelet-I	0	0	1000
wavelet-I	0	0	1000
wavelet-I	0	0	1000
wavelet-I	0	0	1000
wavelet-I	0	0	1000
wavelet-I	0	0	1000
wavelet-I	0	0	1000
wavelet-I	0	0	1000
wavelet-I	0	0	1000
wavelet-I	0	0	1000
wavelet-I	0	0	1000
wavelet-I	0	0	1000
wavelet-I	0	0	1000
wavelet-I	0	0	1000
wavelet-I	0	0	1000
wavelet-I	0	0	1000
wavelet-I	0	0	1000
wavelet-I	0	0	1000
wavelet-I	0	0	1000
wavelet-I	0	0	1000
wavelet-I	0	0	1000
wavelet-I	0	0	1000
wavelet-I	0	0	1000
wavelet-I	0	0	1000
wavelet-I	0	0	1000
wavelet-I	0	0	1000
wavelet-I	0	0	1000
wavelet-I	0	0	1000
wavelet-I	0	0	1000
wavelet-I	0	0	1000
wavelet-I	0	0	1000
wavelet-I	0	0	1000
wavelet-I	0	0	1000
wavelet-I	0	0	1000
wavelet-I	0	0	1000
wavelet-I	0	0	1000
wavelet-I	0	0	1000
wavelet-I	0	0	1000
wavelet-I	0	0	1000
wavelet-I	0	0	1000
wavelet-I	0	0	1000
wavelet-I	0	0	1000
wavelet-I	0	0	1000

wavelet-I	0	0	1000
wavelet-I	0	0	1000
wavelet-I	0	0	1000
wavelet-I	0	0	1000
wavelet-I	0	0	1000
wavelet-I	0	0	1000
wavelet-I	0	0	1000
wavelet-I	0	0	1000
wavelet-I	0	0	1000
wavelet-I	0	0	1000
wavelet-I	0	0	1000
wavelet-I	0	0	1000
wavelet-I	0	0	1000
wavelet-I	0	0	1000
wavelet-I	0	0	1000
wavelet-I	0	0	1000
wavelet-I	0	0	1000
wavelet-I	0	0	1000
wavelet-I	0	0	1000
wavelet-I	0	0	1000
wavelet-I	0	0	1000
wavelet-I	0	0	1000
wavelet-I	0	0	1000
wavelet-I	0	0	1000
wavelet-I	0	0	1000
wavelet-I	0	0	1000
wavelet-I	0	0	1000
wavelet-I	0	0	1000
wavelet-I	0	0	1000
wavelet-I	0	0	1000
wavelet-I	0	0	1000
wavelet-I	0	0	1000
wavelet-I	0	0	1000
wavelet-I	0	0	1000
wavelet-I	0	0	1000
wavelet-I	0	0	1000
wavelet-I	0	0	1000
wavelet-I	0	0	1000
wavelet-I	0	0	1000
wavelet-I	0	0	1000
wavelet-I	0	0	1000
wavelet-I	0	0	1000
wavelet-I	0	0	1000
wavelet-I	0	0	1000
wavelet-I	0	0	1000
wavelet-I	0	0	1000
wavelet-I	0	0	1000
wavelet-I	0	0	1000
wavelet-I	0	0	1000
wavelet-I	0	0	1000
wavelet-I	0	0	1000
wavelet-I	0	0	1000
wavelet-I	0	0	1000
wavelet-I	0	0	1000

wavelet-I	0	0	1000
wavelet-I	0	0	1000
wavelet-I	0	0	1000
wavelet-I	0	0	1000
wavelet-I	0	0	1000
wavelet-I	0	0	1000
wavelet-I	0	0	1000
wavelet-I	0	0	1000
wavelet-I	0	0	1000
wavelet-I	0	0	1000
wavelet-I	0	0	1000
wavelet-I	0	0	1000
wavelet-I	0	0	1000
wavelet-I	0	0	1000
wavelet-I	0	0	1000
wavelet-I	0	0	1000
wavelet-I	0	0	1000
wavelet-I	0	0	1000
wavelet-I	0	0	1000
wavelet-I	0	0	1000
wavelet-I	0	0	1000
wavelet-I	0	0	1000
wavelet-I	0	0	1000
wavelet-I	0	0	1000
wavelet-I	0	0	1000
wavelet-I	0	0	1000
wavelet-I	0	0	1000
wavelet-I	0	0	1000
wavelet-I	0	0	1000
wavelet-I	0	0	1000
wavelet-I	0	0	1000
wavelet-I	0	0	1000
wavelet-I	0	0	1000
wavelet-I	0	0	1000
wavelet-I	0	0	1000
wavelet-I	0	0	1000
wavelet-I	0	0	1000
wavelet-I	0	0	1000
wavelet-I	0	0	1000
wavelet-I	0	0	1000
wavelet-I	0	0	1000
wavelet-I	0	0	1000
wavelet-I	0	0	1000
wavelet-I	0	0	1000
wavelet-I	0	0	1000
wavelet-I	0	0	1000
wavelet-I	0	0	1000
wavelet-I	0	0	1000
wavelet-I	0	0	1000
wavelet-I	0	0	1000
wavelet-I	0	0	1000
wavelet-I	0	0	1000
wavelet-I	0	0	1000
wavelet-I	0	0	1000

wavelet-L	0	0	1000
wavelet-L	0	0	1000
wavelet-L	0	0	1000
wavelet-L	0	0	1000
wavelet-L	0	0	1000
wavelet-L	0	0	1000
wavelet-L	0	0	1000
wavelet-L	0	0	1000
wavelet-L	0	0	1000
wavelet-L	0	0	1000
wavelet-L	0	0	1000
wavelet-L	0	0	1000
wavelet-L	0	0	1000
wavelet-L	0	0	1000
wavelet-H	0	0	1000
wavelet-H	0	0	1000
wavelet-H	0	0	1000
wavelet-H	0	0	1000
wavelet-H	0	0	1000
wavelet-H	0	0	1000
wavelet-H	0	0	1000
wavelet-H	0	0	1000
wavelet-H	0	0	1000
wavelet-H	0	0	1000
wavelet-H	0	0	1000
wavelet-H	0	0	1000
wavelet-H	0	0	1000
wavelet-H	0	0	1000
wavelet-H	0	0	1000
wavelet-H	0	0	1000
wavelet-H	0	0	1000
wavelet-H	0	0	1000
wavelet-H	0	0	1000
wavelet-H	0	0	1000
wavelet-H	0	0	1000
wavelet-H	0	0	1000
wavelet-H	0	0	1000
wavelet-H	0	0	1000
wavelet-H	0	0	1000
wavelet-H	0	0	1000
wavelet-H	0	0	1000
wavelet-H	0	0	1000
wavelet-H	0	0	1000
wavelet-H	0	0	1000
wavelet-H	0	0	1000
wavelet-H	0	0	1000
wavelet-H	0	0	1000
wavelet-H	0	0	1000
wavelet-H	0	0	1000
wavelet-H	0	0	1000
wavelet-H	0	0	1000
wavelet-H	0	0	1000
wavelet-H	0	0	1000
wavelet-H	0	0	1000

wavelet-H	0	0	1000
wavelet-H	0	0	1000
wavelet-H	0	0	1000
wavelet-H	0	0	1000
wavelet-H	0	0	1000
wavelet-H	0	0	1000
wavelet-H	0	0	1000
wavelet-H	0	0	1000
wavelet-H	0	0	1000
wavelet-H	0	0	1000
wavelet-H	0	0	1000
wavelet-H	0	0	1000
wavelet-H	0	0	1000
wavelet-H	0	0	1000
wavelet-H	0	0	1000
wavelet-H	0	0	1000
wavelet-H	0	0	1000
wavelet-H	0	0	1000
wavelet-H	0	0	1000
wavelet-H	0	0	1000
wavelet-H	0	0	1000
wavelet-H	0	0	1000
wavelet-H	0	0	1000
wavelet-H	0	0	1000
wavelet-H	0	0	1000
wavelet-H	0	0	1000
wavelet-H	0	0	1000
wavelet-H	0	0	1000
wavelet-H	0	0	1000
wavelet-H	0	0	1000
wavelet-H	0	0	1000
wavelet-H	0	0	1000
wavelet-H	0	0	1000
wavelet-H	0	0	1000
wavelet-H	0	0	1000
wavelet-H	0	0	1000
wavelet-H	0	0	1000
wavelet-H	0	0	1000
wavelet-H	0	0	1000
wavelet-H	0	0	1000
wavelet-H	0	0	1000
wavelet-H	0	0	1000
wavelet-H	0	0	1000
wavelet-H	0	0	1000
wavelet-H	0	0	1000
wavelet-H	0	0	1000
wavelet-H	0	0	1000
wavelet-H	0	0	1000
wavelet-H	0	0	1000
wavelet-H	0	0	1000
wavelet-H	0	0	1000
wavelet-H	0	0	1000
wavelet-H	0	0	1000
wavelet-H	0	0	1000

wavelet-H	0	0	1000
wavelet-H	0	0	1000
wavelet-H	0	0	1000
wavelet-H	0	0	1000
wavelet-H	0	0	1000
wavelet-H	0	0	1000
wavelet-H	0	0	1000
wavelet-H	0	0	1000
wavelet-H	0	0	1000
wavelet-H	0	0	1000
wavelet-H	0	0	1000
wavelet-H	0	0	1000
wavelet-H	0	0	1000
wavelet-H	0	0	1000
wavelet-H	0	0	1000
wavelet-H	0	0	1000
wavelet-H	0	0	1000
wavelet-H	0	0	1000
wavelet-H	0	0	1000
wavelet-H	0	0	1000
wavelet-H	0	0	1000
wavelet-H	0	0	1000
wavelet-H	0	0	1000
wavelet-H	0	0	1000
wavelet-H	0	0	1000
wavelet-H	0	0	1000
wavelet-H	0	0	1000
wavelet-H	0	0	1000
wavelet-H	0	0	1000
wavelet-H	0	0	1000
wavelet-H	0	0	1000
wavelet-H	0	0	1000
wavelet-H	0	0	1000
wavelet-H	0	0	1000
wavelet-H	0	0	1000
wavelet-H	0	0	1000
wavelet-H	0	0	1000
wavelet-H	0	0	1000
wavelet-H	0	0	1000
wavelet-H	0	0	1000
wavelet-H	0	0	1000
wavelet-H	0	0	1000
wavelet-H	0	0	1000
wavelet-H	0	0	1000
wavelet-H	0	0	1000
wavelet-H	0	0	1000
wavelet-H	0	0	1000
wavelet-H	0	0	1000
wavelet-H	0	0	1000
wavelet-H	0	0	1000
wavelet-H	0	0	1000
wavelet-H	0	0	1000
wavelet-H	0	0	1000
wavelet-H	0	0	1000

wavelet-H	0	0	1000
wavelet-H	0	0	1000
wavelet-H	0	0	1000
wavelet-H	0	0	1000
wavelet-H	0	0	1000
wavelet-H	0	0	1000
wavelet-H	0	0	1000
wavelet-H	0	0	1000
wavelet-H	0	0	1000
wavelet-H	0	0	1000
wavelet-H	0	0	1000
wavelet-H	0	0	1000
wavelet-H	0	0	1000
wavelet-H	0	0	1000
wavelet-H	0	0	1000
wavelet-H	0	0	1000
wavelet-H	0	0	1000
wavelet-H	0	0	1000
wavelet-H	0	0	1000
wavelet-H	0	0	1000
wavelet-H	0	0	1000
wavelet-H	0	0	1000
wavelet-H	0	0	1000
wavelet-H	0	0	1000
wavelet-H	0	0	1000
wavelet-H	0	0	1000
wavelet-H	0	0	1000
wavelet-H	0	0	1000
wavelet-H	0	0	1000
wavelet-H	0	0	1000
wavelet-H	0	0	1000
wavelet-H	0	0	1000
wavelet-H	0	0	1000
wavelet-H	0	0	1000
wavelet-H	0	0	1000
wavelet-H	0	0	1000
wavelet-H	0	0	1000
wavelet-H	0	0	1000
wavelet-H	0	0	1000
wavelet-H	0	0	1000
wavelet-H	0	0	1000
wavelet-H	0	0	1000
wavelet-H	0	0	1000
wavelet-H	0	0	1000
wavelet-H	0	0	1000
wavelet-H	0	0	1000
wavelet-H	0	0	1000
wavelet-H	0	0	1000
wavelet-H	0	0	1000
wavelet-H	0	0	1000
wavelet-H	0	0	1000
wavelet-H	0	0	1000
wavelet-H	0	0	1000
wavelet-H	0	0	1000

wavelet-H	0	0	1000
wavelet-H	0	0	1000
wavelet-H	0	0	1000
wavelet-H	0	0	1000
wavelet-H	0	0	1000
wavelet-H	0	0	1000
wavelet-H	0	0	1000
wavelet-H	0	0	1000
wavelet-H	0	0	1000
wavelet-H	0	0	1000
wavelet-H	0	0	1000
wavelet-H	0	0	1000
wavelet-H	0	0	1000
wavelet-H	0	0	1000
wavelet-H	0	0	1000
wavelet-H	0	0	1000
wavelet-H	0	0	1000
wavelet-H	0	0	1000
wavelet-H	0	0	1000
wavelet-H	0	0	1000
wavelet-H	0	0	1000
wavelet-H	0	0	1000
wavelet-H	0	0	1000
wavelet-H	0	0	1000
wavelet-H	0	0	1000
wavelet-H	0	0	1000
wavelet-H	0	0	1000
wavelet-H	0	0	1000
wavelet-H	0	0	1000
wavelet-H	0	0	1000
wavelet-H	0	0	1000
wavelet-H	0	0	1000
wavelet-H	0	0	1000
wavelet-H	0	0	1000
wavelet-H	0	0	1000
wavelet-H	0	0	1000
wavelet-H	0	0	1000
wavelet-H	0	0	1000
wavelet-H	0	0	1000
wavelet-H	0	0	1000
wavelet-H	0	0	1000
wavelet-H	0	0	1000
wavelet-H	0	0	1000
wavelet-H	0	0	1000
wavelet-H	0	0	1000
wavelet-H	0	0	1000
wavelet-H	0	0	1000
wavelet-H	0	0	1000
wavelet-H	0	0	1000
wavelet-H	0	0	1000
wavelet-H	0	0	1000
wavelet-H	0	0	1000
wavelet-H	0	0	1000
wavelet-H	0	0	1000

wavelet-H	0	0	1000
wavelet-H	0	0	1000
wavelet-H	0	0	1000
wavelet-H	0	0	1000
wavelet-H	0	0	1000
wavelet-H	0	0	1000
wavelet-H	0	0	1000
wavelet-H	0	0	1000
wavelet-H	0	0	1000
wavelet-H	0	0	1000
wavelet-H	0	0	1000
wavelet-H	0	0	1000
wavelet-H	0	0	1000
wavelet-H	0	0	1000
wavelet-H	0	0	1000
wavelet-H	0	0	1000
wavelet-H	0	0	1000
wavelet-H	0	0	1000
wavelet-H	0	0	1000
wavelet-H	0	0	1000
wavelet-H	0	0	1000
wavelet-H	0	0	1000
wavelet-H	0	0	1000
wavelet-H	0	0	1000
wavelet-H	0	0	1000
wavelet-H	0	0	1000
wavelet-H	0	0	1000
wavelet-H	0	0	1000
wavelet-H	0	0	1000
wavelet-H	0	0	1000
wavelet-H	0	0	1000
wavelet-H	0	0	1000
wavelet-H	0	0	1000
wavelet-H	0	0	1000
wavelet-H	0	0	1000
wavelet-H	0	0	1000
wavelet-H	0	0	1000
wavelet-H	0	0	1000
wavelet-H	0	0	1000
wavelet-H	0	0	1000
wavelet-H	0	0	1000
wavelet-H	0	0	1000
wavelet-H	0	0	1000
wavelet-H	0	0	1000
wavelet-H	0	0	1000
wavelet-H	0	0	1000
wavelet-H	0	0	1000
wavelet-H	0	0	1000
wavelet-H	0	0	1000
wavelet-H	0	0	1000
wavelet-H	0	0	1000
wavelet-H	0	0	1000
wavelet-H	0	0	1000
wavelet-H	0	0	1000

wavelet-H	0	0	1000
wavelet-H	0	0	1000
wavelet-H	0	0	1000
wavelet-H	0	0	1000
wavelet-H	0	0	1000
wavelet-H	0	0	1000
wavelet-H	0	0	1000
wavelet-H	0	0	1000
wavelet-H	0	0	1000
wavelet-H	0	0	1000
wavelet-H	0	0	1000
wavelet-H	0	0	1000
wavelet-H	0	0	1000
wavelet-H	0	0	1000
wavelet-H	0	0	1000
wavelet-H	0	0	1000
wavelet-H	0	0	1000
wavelet-H	0	0	1000
wavelet-H	0	0	1000
wavelet-H	0	0	1000
wavelet-H	0	0	1000
wavelet-L	0	0	1000
wavelet-L	0	0	1000
wavelet-L	0	0	1000
wavelet-L	0	0	1000
wavelet-L	0	0	1000
wavelet-L	0	0	1000
wavelet-L	0	0	1000
wavelet-L	0	0	1000
wavelet-L	0	0	1000
wavelet-L	0	0	1000
wavelet-L	0	0	1000
wavelet-L	0	0	1000
wavelet-L	0	0	1000
wavelet-L	0	0	1000
wavelet-L	0	0	1000
wavelet-L	0	0	1000
wavelet-L	0	0	1000
wavelet-L	0	0	1000
wavelet-L	0	0	1000
wavelet-L	0	0	1000
wavelet-L	0	0	1000
wavelet-L	0	0	1000
wavelet-L	0	0	1000
wavelet-L	0	0	1000
wavelet-L	0	0	1000
wavelet-L	0	0	1000
wavelet-L	0	0	1000
wavelet-L	0	0	1000
wavelet-L	0	0	1000
wavelet-L	0	0	1000
wavelet-L	0	0	1000
wavelet-L	0	0	1000
wavelet-L	0	0	1000

wavelet-I	0	0	1000
wavelet-I	0	0	1000
wavelet-I	0	0	1000
wavelet-I	0	0	1000
wavelet-I	0	0	1000
wavelet-I	0	0	1000
wavelet-I	0	0	1000
wavelet-I	0	0	1000
wavelet-I	0	0	1000
wavelet-I	0	0	1000
wavelet-I	0	0	1000
wavelet-I	0	0	1000
wavelet-I	0	0	1000
wavelet-I	0	0	1000
wavelet-I	0	0	1000
wavelet-I	0	0	1000
wavelet-I	0	0	1000
wavelet-I	0	0	1000
wavelet-I	0	0	1000
wavelet-I	0	0	1000
wavelet-I	0	0	1000
wavelet-I	0	0	1000
wavelet-I	0	0	1000
wavelet-I	0	0	1000
wavelet-I	0	0	1000
wavelet-I	0	0	1000
wavelet-I	0	0	1000
wavelet-I	0	0	1000
wavelet-I	0	0	1000
wavelet-I	0	0	1000
wavelet-I	0	0	1000
wavelet-I	0	0	1000
wavelet-I	0	0	1000
wavelet-I	0	0	1000
wavelet-I	0	0	1000
wavelet-I	0	0	1000
wavelet-I	0	0	1000
wavelet-I	0	0	1000
wavelet-I	0	0	1000
wavelet-I	0	0	1000
wavelet-I	0	0	1000
wavelet-I	0	0	1000
wavelet-I	0	0	1000
wavelet-I	0	0	1000
wavelet-I	0	0	1000
wavelet-I	0	0	1000
wavelet-I	0	0	1000
wavelet-I	0	0	1000
log-sigma	0	0	1000
log-sigma	0	0	1000
log-sigma	0	0	1000
log-sigma	0	0	1000
log-sigma	0	0	1000
log-sigma	0	0	1000

log-sigma	0	0	1000
log-sigma	0	0	1000
log-sigma	0	0	1000
log-sigma	0	0	1000
log-sigma	0	0	1000
log-sigma	0	0	1000
log-sigma	0	0	1000
log-sigma	0	0	1000
log-sigma	0	0	1000
log-sigma	0	0	1000
log-sigma	0	0	1000
log-sigma	0	0	1000
log-sigma	0	0	1000
log-sigma	0	0	1000
log-sigma	0	0	1000
log-sigma	0	0	1000
log-sigma	0	0	1000
log-sigma	0	0	1000
log-sigma	0	0	1000
log-sigma	0	0	1000
log-sigma	0	0	1000
log-sigma	0	0	1000
log-sigma	0	0	1000
log-sigma	0	0	1000
log-sigma	0	0	1000
log-sigma	0	0	1000
log-sigma	0	0	1000
log-sigma	0	0	1000
log-sigma	0	0	1000
log-sigma	0	0	1000
log-sigma	0	0	1000
log-sigma	0	0	1000
log-sigma	0	0	1000
log-sigma	0	0	1000
log-sigma	0	0	1000
log-sigma	0	0	1000
log-sigma	0	0	1000
log-sigma	0	0	1000
log-sigma	0	0	1000
log-sigma	0	0	1000
log-sigma	0	0	1000
log-sigma	0	0	1000
log-sigma	0	0	1000
log-sigma	0	0	1000
log-sigma	0	0	1000
log-sigma	0	0	1000
log-sigma	0	0	1000
log-sigma	0	0	1000
log-sigma	0	0	1000
log-sigma	0	0	1000
log-sigma	0	0	1000
log-sigma	0	0	1000
log-sigma	0	0	1000
log-sigma	0	0	1000

log-sigma	0	0	1000
log-sigma	0	0	1000
log-sigma	0	0	1000
log-sigma	0	0	1000
log-sigma	0	0	1000
log-sigma	0	0	1000
log-sigma	0	0	1000
log-sigma	0	0	1000
log-sigma	0	0	1000
log-sigma	0	0	1000
log-sigma	0	0	1000
log-sigma	0	0	1000
log-sigma	0	0	1000
log-sigma	0	0	1000
log-sigma	0	0	1000
log-sigma	0	0	1000
log-sigma	0	0	1000
log-sigma	0	0	1000
log-sigma	0	0	1000
log-sigma	0	0	1000
log-sigma	0	0	1000
log-sigma	0	0	1000
log-sigma	0	0	1000
log-sigma	0	0	1000
log-sigma	0	0	1000
log-sigma	0	0	1000
log-sigma	0	0	1000
log-sigma	0	0	1000
log-sigma	0	0	1000
log-sigma	0	0	1000
log-sigma	0	0	1000
log-sigma	0	0	1000
log-sigma	0	0	1000
log-sigma	0	0	1000
log-sigma	0	0	1000
log-sigma	0	0	1000
log-sigma	0	0	1000
log-sigma	0	0	1000
log-sigma	0	0	1000
log-sigma	0	0	1000
log-sigma	0	0	1000
log-sigma	0	0	1000
log-sigma	0	0	1000
log-sigma	0	0	1000
log-sigma	0	0	1000
log-sigma	0	0	1000
log-sigma	0	0	1000
log-sigma	0	0	1000
log-sigma	0	0	1000
log-sigma	0	0	1000
log-sigma	0	0	1000
log-sigma	0	0	1000
log-sigma	0	0	1000
log-sigma	0	0	1000

log-sigma	0	0	1000
log-sigma	0	0	1000
log-sigma	0	0	1000
log-sigma	0	0	1000
log-sigma	0	0	1000
log-sigma	0	0	1000
log-sigma	0	0	1000
log-sigma	0	0	1000
log-sigma	0	0	1000
log-sigma	0	0	1000
log-sigma	0	0	1000
log-sigma	0	0	1000
log-sigma	0	0	1000
log-sigma	0	0	1000
log-sigma	0	0	1000
log-sigma	0	0	1000
log-sigma	0	0	1000
log-sigma	0	0	1000
log-sigma	0	0	1000
log-sigma	0	0	1000
log-sigma	0	0	1000
log-sigma	0	0	1000
log-sigma	0	0	1000
log-sigma	0	0	1000
log-sigma	0	0	1000
log-sigma	0	0	1000
log-sigma	0	0	1000
log-sigma	0	0	1000
log-sigma	0	0	1000
log-sigma	0	0	1000
log-sigma	0	0	1000
log-sigma	0	0	1000
log-sigma	0	0	1000
log-sigma	0	0	1000
log-sigma	0	0	1000
log-sigma	0	0	1000
log-sigma	0	0	1000
log-sigma	0	0	1000
log-sigma	0	0	1000
log-sigma	0	0	1000
log-sigma	0	0	1000
log-sigma	0	0	1000
log-sigma	0	0	1000
log-sigma	0	0	1000
log-sigma	0	0	1000
log-sigma	0	0	1000
log-sigma	0	0	1000
log-sigma	0	0	1000
log-sigma	0	0	1000
log-sigma	0	0	1000
log-sigma	0	0	1000
log-sigma	0	0	1000
log-sigma	0	0	1000
log-sigma	-1.92E-05	8.91E-05	1000

wavelet-H	-2.69E-05	0.00028021	1000
wavelet-H	-4.03E-05	7.98E-05	1000
wavelet-L	-4.99E-05	2.31E-05	1000
original_	-5.38E-05	0.000243128	1000
wavelet-L	-6.15E-05	0.000148513	1000
wavelet-L	-8.84E-05	0.000178924	1000
wavelet-H	-9.99E-05	0.00052022	1000
wavelet-H	-0.000147928	0.00067799	1000
wavelet-H	-0.00025359	0.000749751	1000
log-sigma	-0.000315067	0.000427702	1000
wavelet-L	-0.000326593	0.000177432	1000
wavelet-H	-0.000368858	0.000163195	1000
wavelet-L	-0.000407281	0.00023938	1000
wavelet-H	-0.00043994	0.000286848	1000
wavelet-L	-0.00048989	0.000784191	1000
wavelet-L	-0.000509101	0.000111691	1000
log-sigma	-0.000511023	0.000505423	1000
wavelet-H	-0.000541761	0.000409775	1000
log-sigma	-0.000618606	0.000486719	1000
wavelet-H	-0.000770376	0.00177187	1000
wavelet-H	-0.000949042	0.0016185	1000
log-sigma	-0.00132174	0.00145866	1000
log-sigma	-0.00146583	0.000734382	1000
wavelet-H	-0.00195572	0.00166026	1000

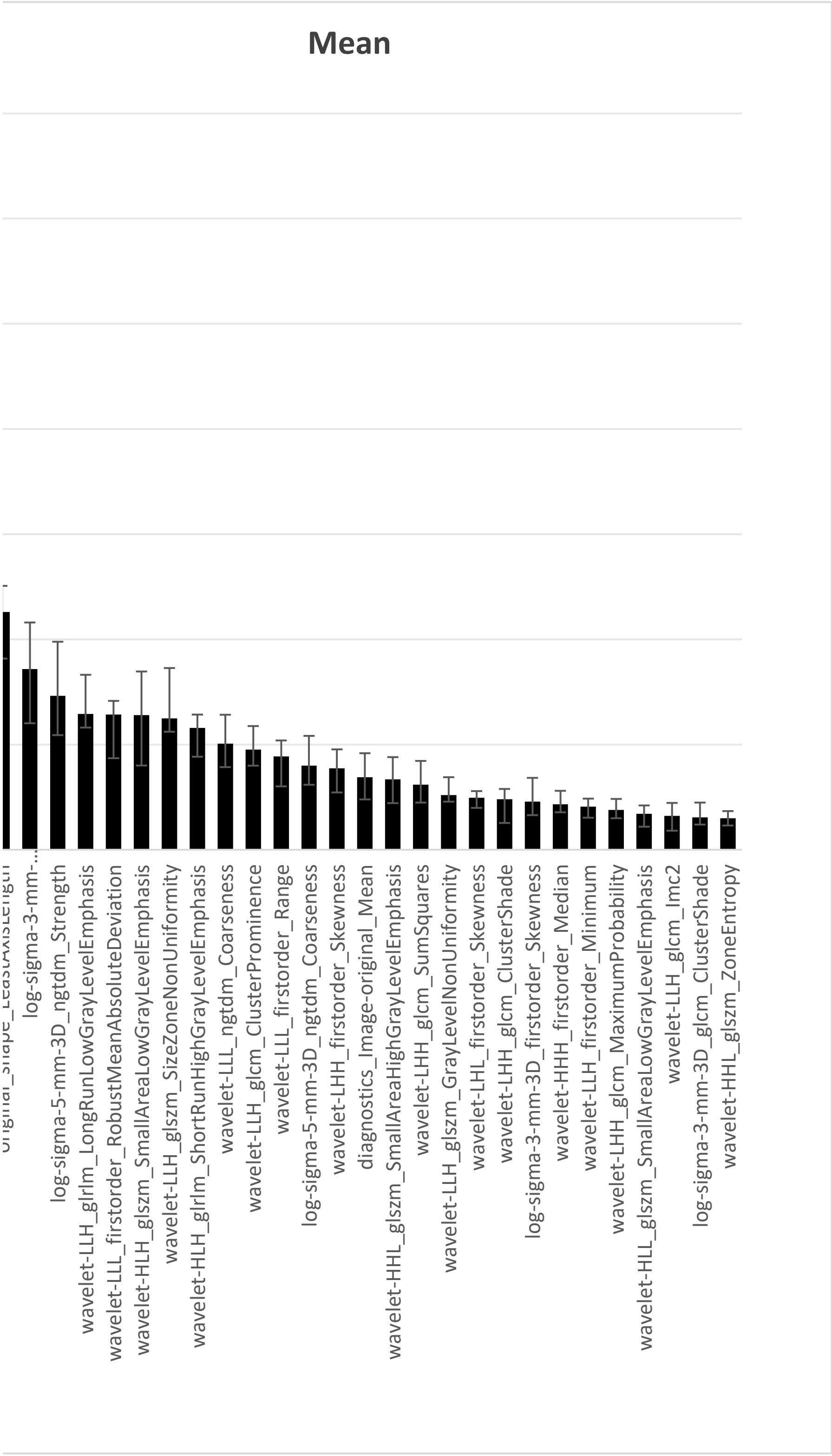
Mean
log-sigma-3-mm-...
log-sigma-5-mm-3D_ngtdm_Strength
wavelet-LLH_glrlm_LongRunLowGrayLevelEmphasis
wavelet-LLL_firstorder_RobustMeanAbsoluteDeviation
wavelet-HLH_glszm_SmallAreaLowGrayLevelEmphasis
wavelet-LLH_glszm_SizeZoneNonUniformity
wavelet-HLH_glrlm_ShortRunHighGrayLevelEmphasis
wavelet-LLL_ngtdm_Coarseness
wavelet-LLH_glcm_ClusterProminence
wavelet-LLL_firstorder_Range
log-sigma-5-mm-3D_ngtdm_Coarseness
wavelet-LHH_firstorder_Skewness
diagnostics_Image-original_Mean
wavelet-HHL_glszm_SmallAreaHighGrayLevelEmphasis
wavelet-LHH_glcm_SumSquares
wavelet-LLH_glszm_GrayLevelNonUniformity
wavelet-LHL_firstorder_Skewness
wavelet-LHH_glcm_ClusterShade
log-sigma-3-mm-3D_firstorder_Skewness
wavelet-HHH_firstorder_Median
wavelet-LLH_firstorder_Minimum
wavelet-LHH_glcm_MaximumProbability
wavelet-HLL_glszm_SmallAreaLowGrayLevelEmphasis
wavelet-LLH_glcm_Imc2
log-sigma-3-mm-3D_glcm_ClusterShade
wavelet-HHL_glszm_ZoneEntropy

Feature	Mean	Std	
MP	0.0624773	0.0101614	1000
HER2	0.0549253	0.00902772	1000
Arm	0.0494405	0.00858271	1000
ER	0.0298506	0.00977588	1000
menopausa	0.00570962	0.0030678	1000
Age_at_S(	0.00338504	0.00132229	1000
ethnicity	0.00210365	0.00117571	1000
Race	0	0	1000

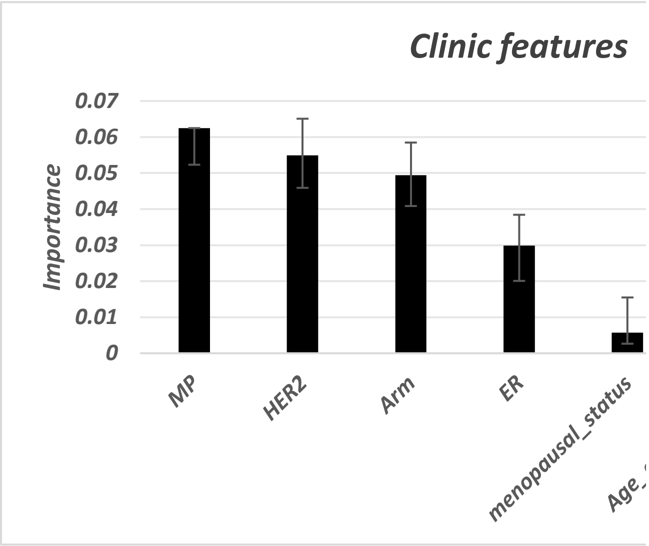

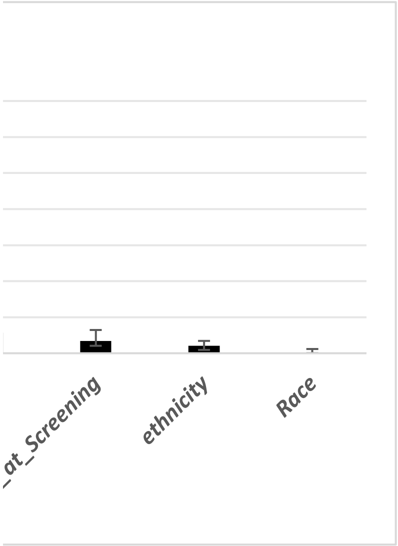

_at_Screening
ethnicity
Race
