# Supplementary material for: Multimodal diagnostic models and subtype analysis for neoadjuvant therapy in breast cancer
Source: Front Immunol. 2025 Mar 18;16:1559200. doi: 10.3389/fimmu.2025.1559200 (PMC11958217; doi:10.3389/fimmu.2025.1559200)
Supplement: Supplementary Figure 3 — Comparison of Four Breast Cancer Multimodal Subtypes. (A, B) Heatmap of protein expression profiles from the ISPY2 cohort across the four breast cancer multimodal subtypes, highlighting the top 20 most significantly different proteins. (C) Significantly different HALLMARKER scores. (D) Significantly different Progeny scores. (E) Significantly different xCell infiltration scores. (F) Significantly different CIBERSORT immune infiltration scores. (G) Significantly different transcription factor activity scores. (H) Significantly different MRI radiomic feature scores. Significance levels are indicated as follows: P<0.05 *, P<0.01 **, P<0.001 ***, P<0.0001 ****, NS (Not Significant). [file DataSheet3.pdf]

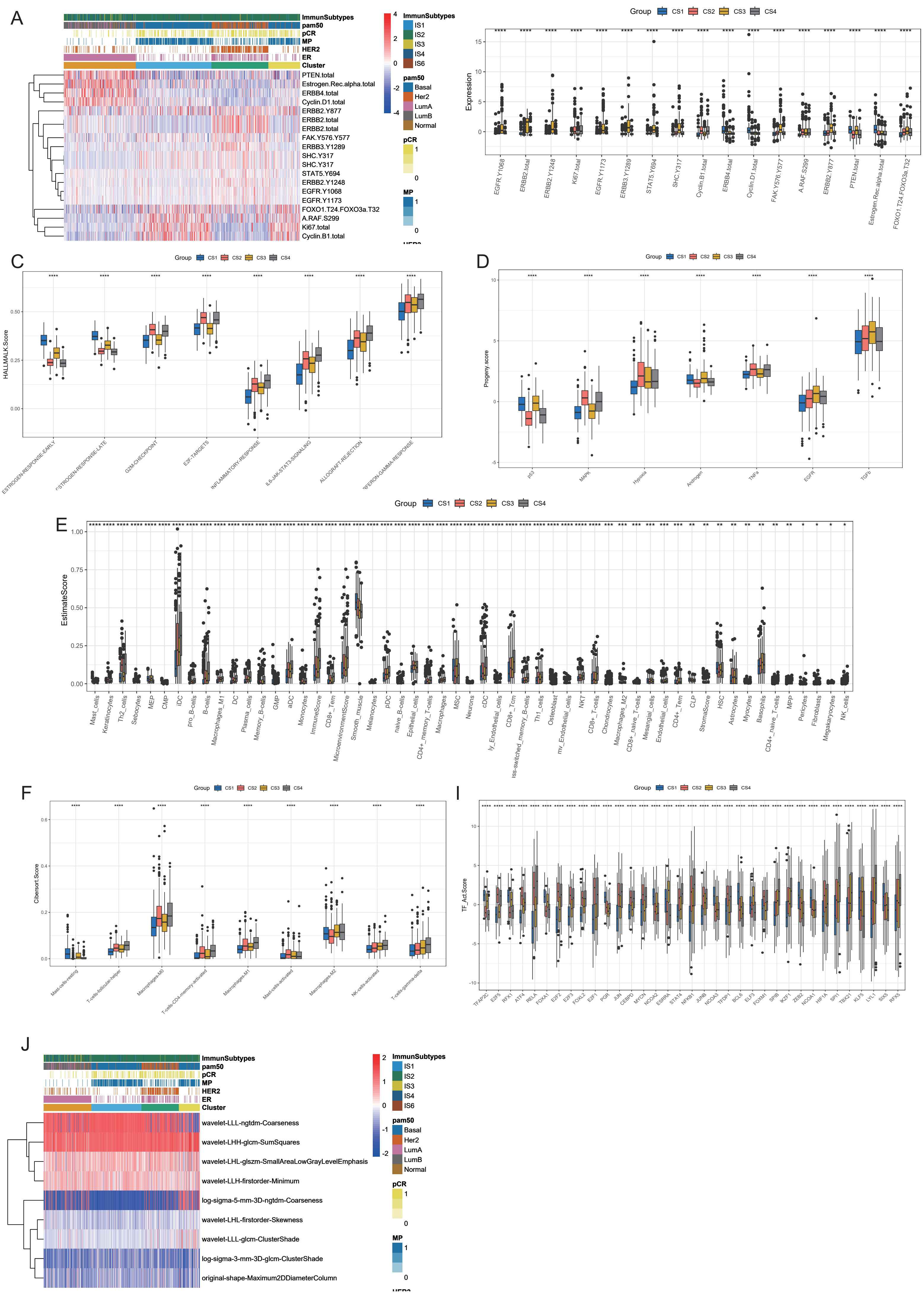
A
ImmunSubtypes
pam50
pCR
MP
HER2
ER
Cluster
PTEN.total
Estrogen.Rec.alpha.total
ERBB4.total
Cyclin.D1.total
ERBB2.Y877
ERBB2.total
ERBB2.total
FAK.Y576.Y577
ERBB3.Y1289
SHC.Y317
SHC.Y317
STAT5.Y694
ERBB2.Y1248
EGFR.Y1068
EGFR.Y1173
FOXO1.T24.FOXO3a.T32
A.RAF.S299
Ki67.total
Cyclin.B1.total
ImmunSubtypes
IS1
IS2
IS3
IS4
IS6
pam50
Basal
Her2
LumA
LumB
Normal
pCR
MP
Group
CS1
CS2
CS3
CS4
Expression
EGFR.Y1068
ERBB2.total
ERBB2.Y1248
Ki67.total
EGFR.Y1173
ERBB3.Y1289
STAT5.Y694
SHC.Y317
Cyclin.B1.total
ERBB4.total
Cyclin.D1.total
FAK.Y576.Y577
A.RAF.S299
ERBB2.Y877
PTEN.total
Estrogen.Rec.alpha.total
FOXO1.T24.FOXO3a.T32
C
HALLMALK.Score
ESTROGEN-RESPONSE-EARLY
ESTROGEN-RESPONSE-LATE
G2M-CHECKPOINT
E2F-TARGETS
INFLAMMATORY-RESPONSE
IL6-JAK-STAT3-SIGNALING
ALLOGRAFT-REJECTION
D
Progeny.score
p53
MAPK
Hypoxia
Androgen
TNFa
EGFR
TGFb
E
EstimateScore
Mast_cells
Keratinocytes
Th2_cells
Sebocytes
MEP
CMP
iDC
pro_B-cells
B-cells
Macrophages_M1
DC
Plasma_cells
Memory_B-cells
GMP
aDC
Monocytes
ImmuneScore
CD8+_Tem
MicroenvironmentScore
Smooth_muscle
Melanocytes
pDC
naive_B-cells
Epithelial_cells
CD4+_memory_T-cells
Macrophages
MSC
Neurons
cDC
CD8+_Tcm
Th1_cells
Osteoblast
NKT
CD8+_T-cells
Chondrocytes
Macrophages_M2
CD8+_naive_T-cells
Mesangial_cells
Endothelial_cells
CD4+_Tem
CLP
StromaScore
HSC
Astrocytes
Myocytes
Basophils
CD4+_naive_T-cells
MPP
Pericytes
Fibroblasts
Megakaryocytes
NK_cells
F
Cibersort.Score
Mast-cells-resting
T-cells-follicular-helper
Macrophages-M0
T-cells-CD4-memory-activated
Macrophages-M1
Mast-cells-activated
Macrophages-M2
NK-cells-activated
T-cells-gamma-delta
I
TF_Act.Score
TFAP2C
E2F5
RFX1
ATF4
RELA
FOXA1
E2F2
E2F3
FOXL2
E2F1
PGR
JUN
CEBPD
MYCN
NCOA2
ESRRA
STAT4
NFKB1
JUNB
NCOA3
TFDP1
BCL6
ELF3
FOXM1
SPIB
IKZF1
ZEB2
NCOA1
HIF1A
SPI1
TBX21
KLF5
LYL1
SIX5
RFX5
J
ImmunSubtypes
pam50
pCR
MP
HER2
ER
Cluster
wavelet-LLL-ngtdm-Coarseness
wavelet-LHH-glcm-SumSquares
wavelet-LHL-glszm-SmallAreaLowGrayLevelEmphasis
wavelet-LLH-firstorder-Minimum
log-sigma-5-mm-3D-ngtdm-Coarseness
wavelet-LHL-firstorder-Skewness
wavelet-LLL-glcm-ClusterShade
log-sigma-3-mm-3D-glcm-ClusterShade
original-shape-Maximum2DDiameterColumn
ImmunSubtypes
IS1
IS2
IS3
IS4
IS6
pam50
Basal
Her2
LumA
LumB
Normal
pCR
MP
